# From assistant to collaborator: A systematic review of the evolution of artificial intelligence in end-stage renal disease care and management

**DOI:** 10.1371/journal.pdig.0001635

**Published:** 2026-09-02

**Authors:** Mohan Wang, Caogen Hong, Zhengxing Huang, Fengmin Shao, Yue Gu

**Affiliations:** 1 College of Computer Science and Technology, Zhejiang University, Hangzhou, China; 2 Polytechnic Institute of Zhejiang University, Zhejiang University, Hangzhou, China; 3 Department of Nephrology, Henan Provincial People’s Hospital, Zhengzhou, China; Peng Cheng Laboratory, CHINA

## Abstract

We aimed to systematically analyze the historical evolution of artificial intelligence (AI) in end-stage renal disease (ESRD) management and propose a developmental framework to map its progression from assistive tools to cognitive collaborators. A systematic review was conducted following PRISMA 2020 guidelines, identifying 100 eligible studies from PubMed, IEEE Xplore, and Web of Science. A three-stage analytical framework was applied to chart the technological evolution of AI in ESRD care: (1) rule-based assistive tools, (2) data-driven learning systems, and (3) emerging large language model- and agent-based cognitive systems. A total of 100 studies were included, all focusing on patients with end-stage renal disease managed through hemodialysis, peritoneal dialysis, or kidney transplantation. Three primary application domains were identified: risk prediction (49.0%), diagnostic support (25.0%), and monitoring and management (26.0%). The analytical framework revealed a developmental progression from interpretable rule-based systems (Stage 1) to high-performing data-driven models (Stage 2), which achieve clinically relevant metrics (e.g., area under the receiver operating characteristic curve [AUC] 0.80–0.90) but often lack external validation. Emerging large language model- and agent-based systems (Stage 3) demonstrate notable versatility but introduce new challenges related to reliability, factual accuracy, and safety alignment. The proposed three-stage framework clarifies AI’s technological and functional evolution in ESRD care. This perspective highlights a critical need to bridge the gap between high-performance modeling and validated clinical utility. Future work should focus on robust external validation and the development of frameworks for the safe, reliable, and ethical deployment of next-generation cognitive AI agents.

## Author summary

People whose kidneys have failed completely depend on dialysis or a kidney transplant for the rest of their lives, and their care generates a constant stream of test results, treatment settings and monitoring data that clinical teams cannot realistically take in as a whole. Over the past two decades researchers have built computer systems to help. We read and analyzed one hundred studies published between 2005 and 2025 to trace how those systems have changed. We found three successive generations: early tools that simply followed rules written down by doctors; a much larger group of systems that learn patterns directly from patient records and now perform well in testing; and a small but rapidly growing group built on the technology behind conversational chatbots, which can handle a far wider range of tasks. We also found that very few of these systems have been tested in hospitals other than the one where they were built, and that none has yet been evaluated while actually guiding care. Our work suggests that the central challenge in this field is no longer building accurate systems, but showing that they help patients safely in everyday practice.

## 1. Introduction

End-stage renal disease (ESRD), as the terminal stage of chronic kidney disease, has emerged as a major global public health challenge [1,2]. ESRD patients require long-term dependence on renal replacement therapy to sustain life, involving complex treatment modalities including hemodialysis, peritoneal dialysis, and kidney transplantation [1]. The management challenges of this disease are manifested at multiple levels: enormous individual patient variability leads to limited effectiveness of standardized treatments, high and unpredictable complication risks, lack of precision in prognostic assessment, and difficulty in effectively integrating and utilizing the massive multidimensional data generated during treatment processes. Traditional medical decision-making primarily relies on physicians’ clinical experience and limited assessment tools, showing significant limitations in handling complex disease pattern recognition, individualized risk prediction, and continuous monitoring management [3]. More critically, with the continuous rise in global ESRD prevalence and increasing treatment complexity, the contradiction between existing medical resources and patient needs is becoming increasingly acute, urgently requiring more precise and efficient diagnostic and therapeutic management approaches [2].

The development of artificial intelligence (AI) technology provides a promising technical pathway to address these challenges. These technologies can integrate multi-source heterogeneous data including laboratory examinations, imaging studies, physiological monitoring, and treatment parameters, discovering disease patterns and risk factors that are difficult to identify through traditional methods, providing quantitative support for clinical decision-making based on evidence-based medicine [4]. Meanwhile, the continuous learning and adaptive capabilities of AI systems enable them to keep pace with rapidly developing medical knowledge, timely integrating the latest clinical guidelines and research evidence to provide up-to-date precision medical services for ESRD patients [5]. The automation and scalability of AI-based tools may also help streamline selected care processes and ease pressure on constrained medical resources, in principle enabling limited professional capacity to reach more patients—a potential benefit that motivates continued investigation but that requires rigorous clinical evaluation before it can be regarded as established [6].

However, existing reviews of AI in ESRD are limited in two important respects. First, most are scoped to a single treatment modality specifically hemodialysis [7,8], peritoneal dialysis [9,10], or kidney transplantation [11–14], and therefore do not capture the full landscape of AI applications across the ESRD care continuum. Second, existing reviews largely overlook the temporal dimension of technological development: they do not systematically examine how AI methodology has evolved across successive developmental stages, nor do they adequately address the characteristics and clinical potential of large language model- and agent-based systems, which have emerged as a distinct and rapidly expanding research direction.

This review addresses these gaps by conducting a systematic analysis grounded in a three-stage technological framework — rule-based assistive tools, data-driven learning systems, and emerging large language model- and agent-based cognitive systems — to characterize the complete AI evolution trajectory in end-stage renal disease (ESRD) care from 2005 to 2025. Throughout this review, the term “AI in ESRD” is used as a collective designation for all artificial intelligence applications in the context of ESRD care and management, encompassing rule-based, machine learning, deep learning, and large language model approaches applied across hemodialysis, peritoneal dialysis, and kidney transplantation. For clarity, the phrases “ESRD care,” “ESRD management,” “ESRD care and management,” and “ESRD diagnosis and treatment management” are used throughout as interchangeable umbrella terms for the full clinical continuum of patients with ESRD—risk prediction, diagnosis, treatment, and longitudinal monitoring—and are not intended to denote distinct sub-domains; where a specific clinical activity (for example, diagnostic support or monitoring and management) is meant, it is named explicitly. Specifically, the review examines: the landscape of AI application domains across hemodialysis, peritoneal dialysis, and kidney transplantation; the three-stage technological evolution from rule-based systems to cognitive AI; model performance and validation characteristics across stages; developmental patterns and barriers to clinical translation; and evidence-based directions for responsible future development.

## 2. Methods

### 2.1. Study design

**Fig 1:**
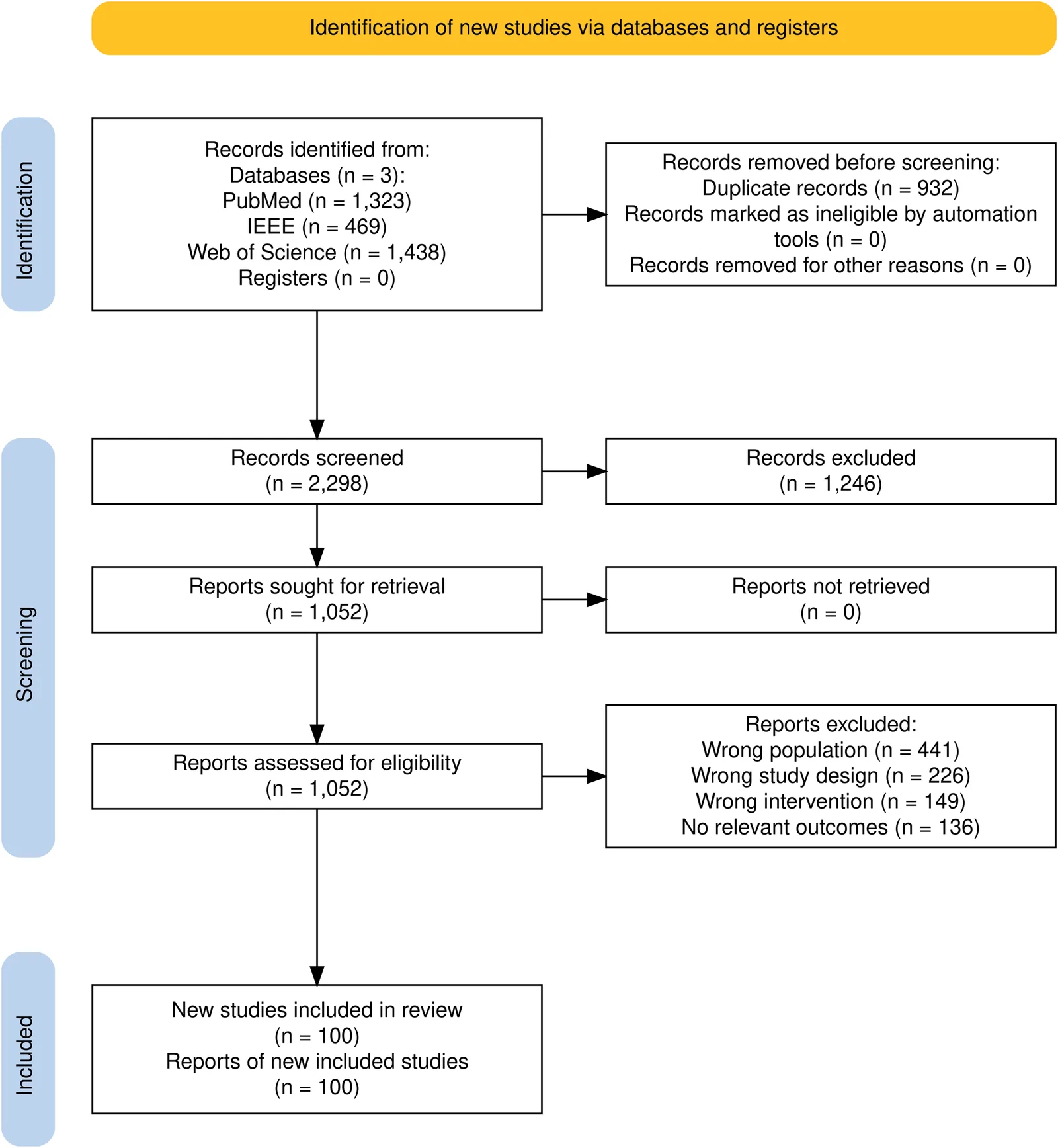
PRISMA 2020 flow diagram of study selection. Initial searches across PubMed (n = 1,323), IEEE Xplore (n = 469), and Web of Science (n = 1,438) yielded 3,230 records. After deduplication and title/abstract screening, 1,052 records were assessed for full-text eligibility; 100 studies were included in the systematic review corpus.

This study employed a review design, strictly adhering to the Preferred Reporting Items for Systematic Reviews and Meta-Analyses (PRISMA) 2020 [15] guidelines for literature search, screening, assessment, and reporting, as illustrated in Fig 1. The completed PRISMA 2020 checklist is provided in S1 File. This review innovatively constructed a three-dimensional analytical framework based on technological development stages, transcending the traditional review paradigm limited to single technical types or treatment modalities, and comprehensively evaluating the application trajectory of artificial intelligence in ESRD diagnosis and treatment management from the historical dimension of technological evolution.

This review was not registered in a prospective systematic review registry such as PROSPERO. To preserve methodological rigor in the absence of registration, the eligibility criteria, search strategy, data-extraction items, and the three-stage analytical framework were specified a priori, before title-and-abstract screening began, and were applied without subsequent modification.

### 2.2. Search Strategy

Literature search employed a comprehensive multi-database, multi-temporal strategy to ensure thoroughness and accuracy of retrieval. The search encompassed three major academic databases: PubMed, IEEE Xplore, and Web of Science. The search timeframe was set from January 2005 to June 2025. This period spans the principal phases of AI development in healthcare: the maturation of rule-based clinical decision support systems through the mid-2000s, the emergence of deep learning as a clinically viable methodology following the demonstration of its transformative potential in image recognition around 2012 [16], the subsequent large-scale adoption of machine learning in clinical research from approximately 2015 onward [17], and the emergence of large language model- and agent-based applications in ESRD care from 2023 onward, as reflected in the seven LLM- and agent-focused studies identified in this review [18–24].

The search strategy combined subject headings with free text terms, with core search terms including artificial intelligence-related terminology (“artificial intelligence”, “machine learning”, “deep learning”, “neural network”, “expert system”, “large language model”, “AI agent”) and end-stage renal disease-related terminology (“end-stage renal disease”, “ESRD”, “hemodialysis”, “peritoneal dialysis”, “kidney transplantation”, “renal replacement therapy”). The complete, database-specific search strings for all three databases are provided in S1 Text.

Grey literature and clinical trial registries (e.g., ClinicalTrials.gov) were not searched, as the scope of this review focused on peer-reviewed original research reporting AI methodology and quantifiable performance outcomes. This is acknowledged as a potential limitation. To ensure search completeness, additional relevant literature was supplemented through reference tracking, expert recommendations, and manual searches.

### 2.3. Inclusion and exclusion criteria

Inclusion criteria:

1. Study population: studies whose population clearly and predominantly comprised patients with established end-stage renal disease (ESRD)—that is, chronic, irreversible kidney failure requiring long-term renal replacement therapy—specifically patients receiving maintenance hemodialysis, maintenance peritoneal dialysis, or kidney transplantation. Studies were eligible only if renal replacement therapy was administered for ESRD rather than for a potentially reversible condition. Where a study reported a mixed dialysis population, it was included only if patients with ESRD constituted the analyzed cohort or were reported as a clearly separable subgroup;
2. Intervention measures: application of artificial intelligence technologies (including rule-based and knowledge-driven ESRD assistive tools, machine learning, deep learning, large language models, or agent technologies) for disease prediction, diagnostic assistance, monitoring and management, or treatment optimization;
3. Study types: original research articles, including cohort studies, cross-sectional studies, case-control studies, randomized controlled trials, and technical validation studies;
4. Language: peer-reviewed literature published in English. The restriction to English-language literature reflects the review team’s capacity for systematic and rigorous linguistic appraisal. While this criterion may exclude relevant studies published in other languages, it ensures consistency of evaluation across included studies. This limitation is explicitly acknowledged in the Limitations section.
5. Data completeness: providing clear research methods, sample information, technical details, and outcome data. Study quality was not applied as an independent eligibility criterion. All peer-reviewed original research articles satisfying inclusion criteria 1–4 were eligible for assessment, with methodological rigor evaluated post-hoc through the structured quality and bias assessment framework described in Section 2.5 (risk-of-bias assessment using PROBAST or QUADAS-2, or reporting-quality evaluation using DECIDE-AI, as appropriate to study design). Studies with unclear methods, incomplete reporting of sample characteristics, or absent outcome data were excluded under exclusion criterion 4 (data quality).

Exclusion criteria:

1. Research content: studies involving only early-stage chronic kidney disease (CKD stages 1–4) without involvement of end-stage renal disease; or studies in which dialysis was provided for acute kidney injury (AKI) or other potentially reversible indications rather than for established ESRD. Studies in which ESRD patients could not be distinguished from non-ESRD dialysis populations (e.g., undifferentiated AKI cohorts or pooled acute/chronic cohorts) were excluded;
2. Technology application: studies that only generally mentioned artificial intelligence concepts but did not specifically describe technological applications;
3. Literature types: data papers and retracted publications, editorial materials, book chapters and letters, reviews, and conference proceedings;
4. Data quality: literature with unclear research methods, incomplete data, or inadequate result reporting;
5. Duplicate publication: studies with overlapping data content or duplicate publications.

### 2.4. Literature screening and data extraction

Literature screening was conducted using a two-stage independent assessment approach. The first stage involved preliminary screening of titles and abstracts, and the second stage conducted detailed full-text evaluation of literature that passed the initial screening. Both stages were performed independently by two reviewers (M.W. and C.H.), each blinded to the other’s decisions. Disagreements at each stage were resolved through structured discussion, with unresolved cases adjudicated by a third reviewer (Z.H.). Data extraction was conducted in duplicate using a standardized extraction form covering study characteristics, AI technology type, stage classification, performance metrics, and clinical application context.

### 2.5. Quality and bias assessment

Given the methodological heterogeneity of the included studies, a tool-specific quality appraisal framework was applied, combining formal risk-of-bias assessment with structured reporting-quality evaluation as appropriate to each study design. Each study was independently appraised by two reviewers using the instrument most suited to its design. Prediction model studies were assessed for risk of bias using the Prediction Model Risk of Bias Assessment Tool (PROBAST) [25], which examines four domains: participants, predictors, outcome, and analysis. Diagnostic accuracy studies were assessed for risk of bias using QUADAS-2 [26], covering patient selection, index test, reference standard, and flow and timing. For studies evaluating advanced AI-assisted clinical decision support systems, we applied DECIDE-AI [27]. We note that DECIDE-AI is a reporting and evaluation guideline for the early-stage clinical evaluation of AI-driven decision support systems rather than a conventional risk-of-bias instrument. We selected it for this subset of studies because their AI-assisted decision-support applications are neither prediction-model nor diagnostic-accuracy studies and therefore fall outside the intended scope of PROBAST and QUADAS-2; in the absence of an established risk-of-bias instrument for such applications, DECIDE-AI was used as a structured framework to appraise reporting completeness across transparency, clinical representativeness, safety reporting, and human–AI integration. Ratings within each domain were assigned as Low, Unclear, or High risk of bias (PROBAST and QUADAS-2) or as Adequate, Partial, or Inadequate reporting (DECIDE-AI). Disagreements between the two primary reviewers were resolved through consensus discussion, with arbitration by a third reviewer where necessary.

Inter-rater reliability was assessed at two levels. First, agreement on the selection of the appropriate assessment tool across all 100 included studies was quantified using Cohen’s kappa; 94 of 99 study pairs with valid data from both raters agreed on tool assignment, yielding κ = 0.87, indicating almost perfect agreement. Of the 100 included studies, 74 were assessed with PROBAST, 18 with QUADAS-2, and 8 with DECIDE-AI. Second, domain-level rating agreement was calculated separately for each tool, reported as percentage of exact agreement alongside Cohen’s kappa for each assessment domain (S1 Table). Across PROBAST domains (74 studies, 296 domain-level observation pairs), observed agreement ranged from 47.3% (D1, Participants) to 86.5% (D4, Analysis); across QUADAS-2 domains (18 studies, 72 observation pairs), observed agreement ranged from 27.8% to 66.7%. For DECIDE-AI, the small number of studies assessed with this instrument (n = 8) precludes stable per-domain estimation; the pooled domain-level weighted kappa (quadratic weights) is therefore reported as the summary statistic for this tool (κ_w = 0.72, substantial agreement). All residual disagreements were resolved by consensus or third-reviewer arbitration prior to final synthesis.

The lower observed agreement for QUADAS-2 (27.8%–66.7%) relative to PROBAST warrants comment. Two factors contribute to it. First, this estimate is based on a small subset of 18 diagnostic accuracy studies (72 domain-level observation pairs), which makes domain-level agreement statistics inherently unstable and sensitive to a small number of discordant ratings. Second, several QUADAS-2 domains—particularly patient selection and flow and timing—required finer judgments about study conduct that were frequently reported incompletely in the primary studies, producing greater initial divergence between reviewers at the independent-rating stage. Crucially, these figures characterize the difficulty of independent first-pass appraisal and not the reliability of the final assessments. Final consensus was reached through a structured procedure: for every domain in which the two reviewers initially diverged, they jointly re-examined the primary article against the relevant QUADAS-2 signaling questions, discussed the basis for each judgment, and revised their ratings to a single agreed classification; where agreement could not be reached, a third reviewer (Z.H.) adjudicated the final rating before it was carried into the synthesis. The ratings reported in S1 Table and used throughout the manuscript therefore reflect adjudicated consensus judgments rather than single-reviewer scores, and the residual inter-rater divergence does not propagate into the reported assessment results. We further note that study quality was not used as an eligibility criterion and that the quality-appraisal results were not used to weight, rank, or exclude studies in any analysis (Section 2.3); the appraisal is reported descriptively to characterize the quality of the evidence base, so that any residual uncertainty in individual domain ratings does not affect the substantive findings or conclusions of this review. We nonetheless report the observed agreement transparently so that readers can appraise the subjectivity inherent in applying these instruments to a methodologically heterogeneous AI literature.

### 2.6. Analytical datasets

This review operated with two distinct analytical datasets, which are used for different purposes throughout the manuscript and should be distinguished clearly.

The first is the systematic review corpus, comprising the 100 peer-reviewed original research articles that met all eligibility criteria following full-text assessment. This corpus is the basis for all stage-level, domain-level, modality-level, and performance analyses reported in Sections 4 and 5, and for the quality and bias assessment reported in Section 2.5. All analyses pertaining to AI technology types, clinical application domains, model performance, and validation approaches are drawn exclusively from these 100 studies.

The second is the bibliometric corpus, comprising the 1,052 records assessed for full-text eligibility following title and abstract screening. This dataset, which corresponds directly to the full-text assessment stage of the PRISMA flow (Fig 1), was used to analyze annual publication trends and keyword trajectories, as reported in Sections 3.1 and 3.4. Geographic distribution, institutional profiles, and international collaboration patterns (Sections 3.2–3.3) were analyzed on the basis of the 100-study systematic review corpus, which provides precise affiliation data for each included study. Using the full-text-assessed records as the bibliometric corpus ensures that all analyzed publications are retrievable in their complete form and that the bibliometric findings are grounded in a well-defined, PRISMA-consistent set. Bibliometric analyses were conducted using custom Python scripts; full methods and outputs are provided in S2 Text.

### 2.7. Stage classification criteria

Each included study was assigned to one of three developmental stages based on the primary AI methodology employed, using the following pre-specified criteria.

Stage 1 (Rule-Based Assistive Tools): Studies were classified as Stage 1 if the AI component was implemented as an explicit, human-authored rule system in which decision logic was defined prior to and independently of the training data. This category encompasses clinical decision support systems based on if-then rule sets, knowledge-engineered expert systems, Bayesian network models with expert-specified priors, and fuzzy logic systems. A defining characteristic is that the system’s behavior is fully determined by the rule structure, with no parameters learned from data.

Stage 2 (Data-Driven Learning Systems): Studies were classified as Stage 2 if the AI component learned a decision function from a labelled training dataset using a supervised, semi-supervised, or unsupervised machine learning algorithm. This category includes classical machine learning algorithms (logistic regression, random forest, support vector machine, gradient boosting), deep learning architectures (convolutional neural networks, recurrent neural networks, transformers applied to structured or imaging data), and reinforcement learning systems operating on clinical monitoring data. Stage 2 classification required that model parameters were estimated from empirical data rather than hand-crafted by domain experts.

Stage 3 (Large Language Model- and Agent-Based Systems): Studies were classified as Stage 3 if the AI component was built upon a large language model (LLM) or multi-agent architecture. This includes direct applications of general-purpose LLMs (e.g., GPT-4, Claude) and domain-adapted biomedical language models (e.g., BioBERT, exKidneyBERT) to clinical tasks, systems employing retrieval-augmented generation or chain-of-thought prompting, and multi-agent frameworks capable of autonomous task planning, tool invocation, or coordinated decision-making. The defining feature is the use of a large-scale pre-trained language model as the primary reasoning component, distinguishing Stage 3 systems from Stage 2 models that process structured or imaging data without a natural-language reasoning backbone.

Where a study employed components from more than one stage (e.g., an LLM combined with a rule-based filter), classification was based on the primary AI methodology — defined as the component driving the core clinical inference reported as the study’s main outcome. Stage assignment was performed independently by two reviewers (M.W. and C.H.) using the criteria above, with disagreements resolved by consensus or third-reviewer adjudication (Z.H.). Stage assignment and assessment-tool selection were treated as two distinct classification steps and were therefore evaluated separately. Inter-rater agreement on stage assignment was computed independently across the 99 study pairs with valid data from both reviewers: the two reviewers initially agreed on the developmental stage for 96 of 99 studies, corresponding to Cohen’s κ = 0.90. This statistic is reported separately from, and should not be conflated with, the tool-selection agreement reported in Section 2.5 (κ = 0.87), as the two steps classify different study attributes—the developmental stage of the primary AI methodology versus the appraisal instrument appropriate to the study design.

### 2.8. Missing data handling

Missing data in this systematic review arose in two distinct contexts: (1) incomplete reporting within individual included studies, and (2) genuinely absent data elements in the extraction form.

For incomplete reporting within included studies (for example, absent confidence intervals, unreported sample sizes, or unspecified validation procedures), the corresponding fields in the data extraction form were recorded as “not reported” rather than imputed or inferred. These entries are reflected in Table 1 as “-” and are not carried forward into any quantitative computation. No imputation of performance metrics, sample sizes, or methodological characteristics was performed at any stage of data extraction or synthesis.

**Table 1:** Comprehensive comparison of representative studies across all AI development stages in ESRD management.

Citation	Domain Classification	Application Classification	Application Purpose	Best Method	Best Performance	Data Scale	Validation Method
**Rule-Based and Knowledge-Driven**							
Schaffer et al. [28]	Hemodialysis	Diagnostic Support	Treatment Decision Support	Rule	--	–	Internal validation (hold-out method)
Raghavan et al. [29]	Hemodialysis	Diagnostic Support	Treatment Decision Support	Rule	--	–	None (-)
Geddes et al. [30]	Hemodialysis	Risk Prediction	Survival Risk Prediction	Modified Charlson Score	PRE: 84.2%	2310	External validation (ambidirectional inception cohort study)
Will et al. [31]	Hemodialysis	Monitoring and Management	Anemia Management	Rule	--	1206	None (-)
Cornalba et al. [32]	Hemodialysis	Monitoring and Management	Clinical Risk Monitoring	Bayesian	--	47	None (-)
Miskulin et al. [33]	Hemodialysis	Monitoring and Management	Anemia Management	Rule	Healthcare staff time spent on anemia management reduced by nearly 50%	8941	None (-)
Chen et al. [34]	Hemodialysis	Monitoring and Management	Arteriovenous Fistula Stenosis	Fuzzy Logic	ACC: 85.71%	42	Internal validation (hold-out method)
Chanliau et al. [35]	Hemodialysis	Monitoring and Management	Arteriovenous Fistula Stenosis	Bayesian	SENS: 92%, SPEC: 75%	90	Internal validation (hold-out method)
Improta et al. [36]	Kidney Transplantation	Diagnostic Support	Treatment Decision Support	Fuzzy Logic	ACC: 93%, SENS: 90%, SPEC: 94%, PRE: 91%, RECALL: 90%, F1: 90%	855	None (-)
Niazkhani et al. [37]	Kidney Transplantation	Diagnostic Support	Treatment Decision Support	Rule	SENS: 100%, SPEC: 100%	100	Internal validation (-)
**Data-Driven and Learning-Based**							
**Peritoneal Dialysis**							
Takeuchi et al. [38]	Peritoneal Dialysis	Diagnostic Support	Rapid Peritonitis Diagnosis	Random Forest	AUC: 0.987	–	None (-)
Zhang et al. [39]	Peritoneal Dialysis	Diagnostic Support	Rapid Peritonitis Diagnosis	Random Forest	AUC: 0.993, SENS: 98.5%, SPEC: 92.6%	83	Internal validation (5-fold cross-validation)
Zhang et al. [40]	Peritoneal Dialysis	Risk Prediction	Cardiovascular Event Risk Prediction	Logistic Regression	C-index: 0.744	3054	Internal validation (bootstrapping)
Grunert et al. [41]	Peritoneal Dialysis	Diagnostic Support	Rapid Assessment of Patient Characteristics	PCA	ACC: 100.0%	20	–
Kong et al. [42]	Peritoneal Dialysis	Risk Prediction	Survival Risk Prediction	Ensemble Learning	ACC: 0.695, SPEC: 0.701, IBC: 0.174, AUROC: 0.757	23992	Internal validation (5-fold cross-validation)
Fung and Li [43]	Peritoneal Dialysis	Diagnostic Support	Rapid Peritonitis Diagnosis	–	–	166	None (-)
Tan et al. [44]	Peritoneal Dialysis	Diagnostic Support	Fluid Overload Diagnosis	CNN	AUC: 0.782	76	Internal validation (hold-out method)
Ma et al. [45]	Peritoneal Dialysis	Risk Prediction	Survival Risk Prediction	–	–	1363	Internal validation (10-fold cross-validation)
Dai et al. [46]	Peritoneal Dialysis	Risk Prediction	Peritonitis Risk Prediction	Logistic Regression	AUC: 0.882	376	Internal validation and external validation (bootstrap method)
Zang et al. [47]	Peritoneal Dialysis	Risk Prediction	Peritonitis Risk Prediction	Random Forest	ACC: 93.70%, SENS: 96.67%, SPEC: 86.49%, PRE: 91.43%, RECALL: 96.67%, F1: 0.9398, AUC: 0.916	508	Internal validation (5-fold cross-validation)
Sapsitthikul et al. [48]	Peritoneal Dialysis	Risk Prediction	Peritonitis Risk Prediction	–	–	546	External validation (ROC curve, PR curve)
Xu et al. [49]	Peritoneal Dialysis	Risk Prediction	Cardiovascular Event Risk Prediction	Transformer	AUC: 0.8767, ACC: 0.8610, SENS: 0.8345, PRE: 0.8796, F1: 0.8565	7539	Internal validation (5-fold cross-validation)
Yang et al. [50]	Peritoneal Dialysis	Risk Prediction	Prognosis Prediction	Gradient Boosting	AUC: 0.80, ACC: 0.78, F1: 0.57, PRC: 0.52, SENS: 0.71, SPEC: 0.75	824	Internal validation (hold-out method)
Liu et al. [51]	Peritoneal Dialysis	Risk Prediction	Cardiovascular Event Risk Prediction	XGBoost	AUC: 0.78, SENS: 93.33%, SPEC: 85.00%, F1: 0.88, PRE: 0.84, ACC: 0.87	293	Internal validation and external validation (5-fold cross-validation)
Lin et al. [52]	Peritoneal Dialysis	Risk Prediction	Cardiovascular Event Risk Prediction	Ensemble Learning	AUC: 0.789, C-index: 0.73	666	Internal validation (10-fold cross-validation)
Xu et al. [53]	Peritoneal Dialysis	Risk Prediction	Peritonitis Risk Prediction	Logistic Regression	AUC: 0.76	371	Internal validation (hold-out method)
Yan et al. [54]	Peritoneal Dialysis	Risk Prediction	Cardiovascular Event Risk Prediction	Random Forest	C-index: 0.725, AUC: 0.836	318	Internal validation (hold-out method)
Arriero-País et al. [55]	Peritoneal Dialysis	Risk Prediction	Cardiovascular Event Risk Prediction	Extra Tree	F1: 0.63, ACC: 0.64, RECALL: 0.65	151	Internal validation and external validation (leave-one-out cross-validation)
Xu et al. [56]	Peritoneal Dialysis	Risk Prediction	Heart Failure Risk Prediction	XGBoost	AUC: 0.83	606	Internal validation and external validation (hold-out method)
**Hemodialysis**							
Thakur et al. [57]	Hemodialysis	Risk Prediction	Clinical Event Occurrence Prediction	k-nearest neighbor (KNN)	AUC: 90.16%, PRE: 96.21%, RECALL: 88.47%	109	Internal validation (stratified k-fold cross-validation)
Wang et al. [58]	Hemodialysis	Risk Prediction	Survival Risk Prediction	CNN	F1: 0.977	1311	–
Ota et al. [59]	Hemodialysis	Monitoring and Management	Arteriovenous Fistula Stenosis	CNN	ACC: 82%, SENS: 66%, SPEC: 87%, AUC: 0.83	20	–
Sheng et al. [60]	Hemodialysis	Risk Prediction	Survival Risk Prediction	XGBoost	AUC: 0.83, ACC: 84.52%, F1: 0.75	11176	Internal validation and external validation (10-fold cross-validation)
Chaudhuri et al. [61]	Hemodialysis	Monitoring and Management	Real-time Blood Volume Monitoring	XGBoost	RECALL: 0.94, PRE: 0.33, SPEC: 0.52, AUC: 0.89	616	Internal validation (hold-out method)
Kim et al. [62]	Hemodialysis	Monitoring and Management	Dry Weight Change Monitoring	XGBoost	ACC: 83.26%	1672	Internal validation (hold-out method)
Lee et al. [63]	Hemodialysis	Monitoring and Management	Hypotension Monitoring	RNN	AUC: 0.94	9292	Internal validation (hold-out method)
Liu et al. [64]	Hemodialysis	Risk Prediction	Clinical Event Occurrence Prediction	multilayer perceptron (MLP)	AUC: 0.83, F1: 0.53, SENS: 0.53, SPEC: 0.96	–	Internal validation (four-fold cross-validation)
Ohara et al. [65]	Hemodialysis	Monitoring and Management	Anemia Management	–	–	227	Internal validation and external validation (Leave one patient out cross-validation)
Yang et al. [66]	Hemodialysis	Risk Prediction	Survival Risk Prediction	RNN	C-index: 0.7680, IBS: 0.1302	1232	Internal validation (hold-out method)
Yang et al. [67]	Hemodialysis	Risk Prediction	Survival Risk Prediction	Cox	C-index: 0.7409	829	Internal validation (-)
Yang et al. [68]	Hemodialysis	Monitoring and Management	Hypotension Monitoring	BSWEGWO_KELM	–	178	Internal validation (10-fold cross-validation)
Yang et al. [69]	Hemodialysis	Risk Prediction	Intradialytic Hypotension Risk Prediction	BSCWJAYA_KELM	–	178	External validation (20-fold cross-validation)
Yang et al. [70]	Hemodialysis	Monitoring and Management	Dry Weight Change Monitoring	Q-Network	–	750	Internal validation (hold-out method)
Zhang et al. [71]	Hemodialysis	Monitoring and Management	Arteriovenous Fistula Stenosis	CNN	AUC: 0.96	1425	Internal validation (hold-out method)
Guinsburg et al. [72]	Hemodialysis	Risk Prediction	COVID Survival Risk Prediction	XGBoost	AUC: 0.78, ACC: 70%	21624	Internal validation (hold-out method, grid search and 5-fold cross validation)
Inoue et al. [73]	Hemodialysis	Monitoring and Management	Dry Weight Change Monitoring	Random Forest	AUC: 0.74, ACC: 0.618, PRE: 0.055, RECALL: 0.751, F1: 0.102	314	Internal validation (hold-out method)
Li et al. [74]	Hemodialysis	Risk Prediction	Cerebral Hemorrhage Risk Prediction	XGBoost	AUC: 0.979, ACC: 0.939, PRE: 0.949, SENS: 0.932, SPEC: 0.952, F1: 0.938	393	Internal validation (hold-out method)
Liao et al. [75]	Hemodialysis	Diagnostic Support	Sarcopenia Identification	Ensemble Learning	SENS: 77.50%, SPEC: 83.13%, F1: 77.32%, AUC: 87.40%	242	Internal validation (stratified shuffle split, SMOTE)
Lee et al. [76]	Hemodialysis	Monitoring and Management	Hypotension Monitoring	CNN	ACC: 0.93, RECALL: 0.55, PRE: 0.41, F1: 0.72, AUC: 0.90, SPEC: 0.95	2007	Internal validation (hold-out method)
Zhang et al. [77]	Hemodialysis	Monitoring and Management	Hypotension Monitoring	XGBoost	AUC: 0.887, SENS: 0.734, SPEC: 0.780, PRE: 0.391	693	Internal validation (hold-out method)
Yang et al. [78]	Hemodialysis	Risk Prediction	Hypoalbuminemia Prediction	LSTM	AUC: 98%, ACC: 95%	1567	Internal validation (k-fold cross-validation, k = 5)
Julkaew et al. [79]	Hemodialysis	Monitoring and Management	Vascular Access Quality Monitoring	CNN	ACC: 0.9213, SENS: 0.8431, SPEC: 0.9614, PRE: 0.8762, F1: 0.8364	398	Internal validation (10-fold cross-validation)
Liu et al. [80]	Hemodialysis	Monitoring and Management	Dialysis Adequacy Monitoring	Random Forest	AUC: 0.873, ACC: 0.846, SENS: 0.601, SPEC: 0.932, PRE: 0.754	406	Internal validation (10-fold nested cross-validation)
Rahimi et al. [81]	Hemodialysis	Risk Prediction	Prognosis Prediction	Gradient Boosting	ACC: 94.8%, SPEC: 93.5%, SENS: 94%, F1: 89.2%, ROC: 92%	980	Internal validation (10-fold cross-validation)
Zhang et al. [82]	Hemodialysis	Monitoring and Management	Arteriovenous Fistula Stenosis	LSTM	AUC: 99%, PRE: 99%, Recall: 97%, F1: 98%, ACC: 97%	800	Internal validation (hold-out method)
Yang et al. [83]	Hemodialysis	Monitoring and Management	Hypotension Monitoring	XGBoost	ACC: 0.858, SENS: 0.858, SPEC: 0.858, AUC: 0.936	412	External validation (using new datasets)
Okubo et al. [84]	Hemodialysis	Risk Prediction	Survival Risk Prediction	Cox	AUC: 0.82, SENS: 78.2%, SPEC: 75.1%	454	Internal validation (10-fold cross-validation, bootstrapping)
Shah et al. [85]	Hemodialysis	Monitoring and Management	Arteriovenous Fistula Stenosis	Bootstrap Forest	ACC: 0.84, SENS: 0.86, SPEC: 0.83, PRE: 0.70, F1: 0.77, AUC: 0.94	366	Internal validation (10-fold cross-validation, 10,000-fold bootstrap)
Shu et al. [86]	Hemodialysis	Monitoring and Management	Arteriovenous Fistula Stenosis	XGBoost	F1: 0.811, ACC: 0.773, PRE: 0.840, RECALL: 0.785, AUC: 0.829, SPEC: 0.754	974	Internal validation (hold-out method, 5-fold cross-validation)
Su et al. [87]	Hemodialysis	Risk Prediction	Survival Risk Prediction	Autoencoder	F1: 0.71, PRE: 0.81	4228	Internal validation (5-fold cross-validation)
**Kidney Transplantation**							
Rashidi Khazaee et al. [88]	Kidney Transplantation	Risk Prediction	Post-operative Transplant Kidney Function Value Prediction	MLP	MSE: 99, MAE: 7.1	675	Internal validation (hold-out method)
Marsh et al. [89]	Kidney Transplantation	Diagnostic Support	Glomerulosclerosis Rate Assessment	CNN	IOU: 0.59, R2: 0.828, RMSE: 0.079	17	Internal validation (6-fold cross-validation)
Mark et al. [90]	Kidney Transplantation	Risk Prediction	Transplant Kidney Survival Factor Identification	Ensemble Learning	C-index: 0.724, IBS: 0.061	163199	Internal validation (10-fold cross-validation)
Peng et al. [91]	Kidney Transplantation	Risk Prediction	COVID Pneumonia Risk Assessment	Support Vector Machine	AUC: 0.940, SENS: 81.7%, SPEC: 92.0%, PRE: 83.6%	146	External validation (k-fold cross-validation, k = 5)
Konieczny et al. [92]	Kidney Transplantation	Risk Prediction	Delayed Graft Function Risk Prediction	Random Forest	ACC: 0.9375, Recall: 0.9375, AUC: 0.91	157	Internal validation (10-fold cross-validation)
Naqvi et al. [93]	Kidney Transplantation	Risk Prediction	Post-operative Transplant Kidney Survival Prediction	Adaptive Boosting	PRE: 86%, Recall: 76%, F1: 81%, AUC: 81%	277316	Internal validation (10-fold stratified cross-validation)
Raynaud et al. [94]	Kidney Transplantation	Risk Prediction	Post-operative Transplant Kidney Survival Prediction	Bayesian	AUC: 0.857	13608	Internal validation and external validation (bootstrap method)
Salvi et al. [95]	Kidney Transplantation	Diagnostic Support	Glomerulosclerosis Rate Assessment	CNN	PRE: 0.9562, RECALL: 0.9535, SENS: 97.02%	83	Internal validation (hold-out method)
Van Loon et al. [96]	Kidney Transplantation	Risk Prediction	Post-operative Transplant Kidney Function Value Prediction	RNN	SENS: 86.1%	933	Internal validation and external validation (5-fold cross-validation)
Farris et al. [97]	Kidney Transplantation	Diagnostic Support	Glomerulosclerosis Rate Assessment	CNN	F1: 0.87, SENS: 93.0%, PRE: 81.0%	449	External validation (-)
Hermsen et al. [98]	Kidney Transplantation	Diagnostic Support	Kidney Transplant Biopsy Lesion Assessment	CNN	ACC: 0.78, RECALL: 0.90	73	External validation (-)
Paquette et al. [99]	Kidney Transplantation	Risk Prediction	Pre-operative Transplant Kidney Survival Prediction	RNN	C-index: 0.659, IBS: 0.15220	180141	Internal validation (5-fold cross-validation)
Vaulet et al. [100]	Kidney Transplantation	Diagnostic Support	Kidney Transplant Biopsy Lesion Assessment	Ensemble Learning	AUC: 0.72	2054	External validation (-)
Yi et al. [101]	Kidney Transplantation	Risk Prediction	Post-operative Transplant Kidney Survival Prediction	CNN	ACC: 90%	404	Internal validation and external validation (10-fold cross-validation)
Kers et al. [102]	Kidney Transplantation	Diagnostic Support	Kidney Transplant Biopsy Lesion Assessment	CNN	AUROC: 0.80	1948	Internal validation and external validation (three times cross-validation)
Zhi et al. [103]	Kidney Transplantation	Diagnostic Support	Rejection Detection	CNN	AUC: 0.745, ACC: 0.667, F1: 0.633, Precision: 0.608, Recall: 0.667	252	Internal validation (5-fold cross-validation)
Jacq et al. [104]	Kidney Transplantation	Diagnostic Support	Kidney Transplant Biopsy Lesion Assessment	CNN	ACC: 81%, RECALL: 71%, F1: 76%, ROC: 0.92, Dice: 0.84	423	External validation (hold-out method)
Nemati et al. [105]	Kidney Transplantation	Risk Prediction	Post-operative Transplant Kidney Survival Prediction	Gradient Boosting	C-index: 0.637, AUC: 0.658	106372	Internal validation (hold-out method, Wilcoxon signed-rank test, Bonferroni correction)
Quinino et al. [106]	Kidney Transplantation	Risk Prediction	Pre-operative Transplant Kidney Survival Prediction	XGBoost	AUC: 0.78, SENS: 0.64, SPEC: 0.78, PRE: 0.44, F1: 0.53	859	Internal validation (hold-out method)
Truchot et al. [107]	Kidney Transplantation	Risk Prediction	Post-operative Transplant Kidney Survival Prediction	Cox	C-index: 0.808, IBS: 0.0543	8422	Internal validation and external validation (hold-out method)
Ali et al. [108]	Kidney Transplantation	Risk Prediction	Pre-operative Transplant Kidney Survival Prediction	XGBoost	C-index: 0.74, AUC: 0.76, IBS: 0.14	29713	Internal validation (hold-out method, 10-fold cross-validation)
Ali et al. [109]	Kidney Transplantation	Risk Prediction	Pre-operative Transplant Kidney Survival Prediction	Cox	AUC: 0.68, IBS: 0.09	66914	External validation (5-fold cross-validation, hold-out method)
Jo et al. [110]	Kidney Transplantation	Risk Prediction	Post-operative Transplant Kidney Survival Prediction	CNN	DICE: 0.97	1074	Internal validation (-)
Kim et al. [111]	Kidney Transplantation	Risk Prediction	Post-operative Transplant Kidney Survival Prediction	XGBoost	AUROC: 0.828, AUPRC: 0.576, F1: 0.74	4077	Internal validation (hold-out cross-validation)
Lee et al. [112]	Kidney Transplantation	Diagnostic Support	Rejection Detection	PCA	ACC: 98.82%	–	Internal validation (10-fold cross-validation)
Liu et al. [113]	Kidney Transplantation	Risk Prediction	Delayed Graft Function Risk Prediction	Random Forest	ROC: 0.905	140	Internal validation (hold-out method)
Niu et al. [114]	Kidney Transplantation	Diagnostic Support	Central Biomarker Identification for Renal Fibrosis	XGBoost	AUC: 0.866	236	Internal validation and external validation (-)
Ye et al. [115]	Kidney Transplantation	Diagnostic Support	Rejection Detection	CNN	AUC: 0.798, ACC: 0.71, PRE: 0.71, SENS: 0.91, F1: 0.80	302	Internal validation (hold-out method)
Shehata et al. [116]	Kidney Transplantation	Diagnostic Support	Rejection Detection	Ensemble Learning	ACC: 94.0%	100	Internal validation (Leave-one-subject-out cross-validation)
Yoo et al. [117]	Kidney Transplantation	Risk Prediction	Pre-operative Transplant Kidney Survival Prediction	Ensemble Learning	AUC: 0.833, AUROC: 0.880, ACC: 0.786	14032	Internal validation and external validation (3-times repeated 10-folds cross-validation, 1000 times bootstrapping)
Aksoy et al. [118]	Kidney Transplantation	Risk Prediction	Transplant Kidney Survival Factor Identification	Logistic Regression	ACC: 96.5%, F1: 91.9%	465	Internal validation (hold-out method)
Ali et al. [119]	Kidney Transplantation	Risk Prediction	Pre-operative Transplant Kidney Survival Prediction	XGBoost	AUC: 0.75, C-index: 0.72, IBS: 0.09	12661	Internal validation (hold-out method)
**Large Language Model- and Agent-Based Systems**							
Wang et al. [120]	Hemodialysis	Monitoring and Management	Nutritional Management	GPT-4	ACC: 72%	–	None (-)
Jin et al. [18]	Peritoneal Dialysis	Monitoring and Management	Nutritional Management	GPT-4	–	35	Prospective pilot RCT (serum prealbumin endpoint)
Mankowski et al. [121]	Kidney Transplantation	Diagnostic Support	Treatment Decision Support	GPT-4V	ACC: 83.33%	–	None (-)
Han et al. [122]	Kidney Transplantation	Diagnostic Support	Treatment Decision Support	ChatGPT	ACC: 95.5%	–	None (-)
Xu et al. [19]	Kidney Transplantation	Monitoring and Management	Patient Education	ChatGPT	ACC: 92%	27	None (-)
Lee et al. [20]	Kidney Transplantation	Monitoring and Management	Patient Education	ChatGPT	–	565	None (-)
Yang et al. [123]	Kidney Transplantation	Diagnostic Support	Treatment Decision Support	BERT (exKidneyBERT)	ACC: 0.710, RECALL: 0.712, F1: 0.708	–	Internal validation (hold-out method)

A dash (–) indicates the item was not reported in the original study; “None (–)” indicates that no formal validation procedure was reported.

For data elements that were genuinely absent across a substantial proportion of studies — most notably calibration statistics (e.g., Brier scores, calibration plots), prospective validation outcomes, and fairness or equity metrics — the absence itself is treated as a substantive finding. The systematic under-reporting of these elements across the included literature is explicitly discussed in the Discussion (Section 8.1) as a structural limitation of the current evidence base.

The narrative synthesis presented in Sections 4–7 is therefore based exclusively on data directly extracted from the original publications. Given the extent of outcome heterogeneity and missing performance data across included studies, formal meta-analysis was not undertaken; instead, performance statistics are reported as ranges and medians derived from the subset of studies providing each metric (see Section 9.4 for a full discussion of this limitation).

## 3. Results

The systematic search of three databases (PubMed, n = 1,323; IEEE Xplore, n = 469; Web of Science, n = 1,438) yielded a total of 3,230 records. After removal of 932 duplicate records, 2,298 unique records were screened by title and abstract, of which 1,246 were excluded. The remaining 1,052 reports were sought for full-text retrieval (all 1,052 were successfully retrieved) and assessed for eligibility. Of these, 952 were excluded (wrong population, n = 441; wrong study design, n = 226; wrong intervention, n = 149; no relevant outcomes, n = 136), leaving 100 studies included in the final review. The complete PRISMA flow is illustrated in Fig 1, and all studies excluded at the full-text stage with their primary reasons for exclusion are listed in S1 Data.

All 100 included studies focused directly on ESRD patients undergoing hemodialysis (n = 40), peritoneal dialysis (n = 21), or kidney transplantation (n = 39). All domain-specific, modality-specific, and stage-level analyses presented in Sections 4 and 5 are therefore based on this single, homogeneous corpus of 100 ESRD-focused studies.

### 3.1. Annual publication trends

**Fig 2:**
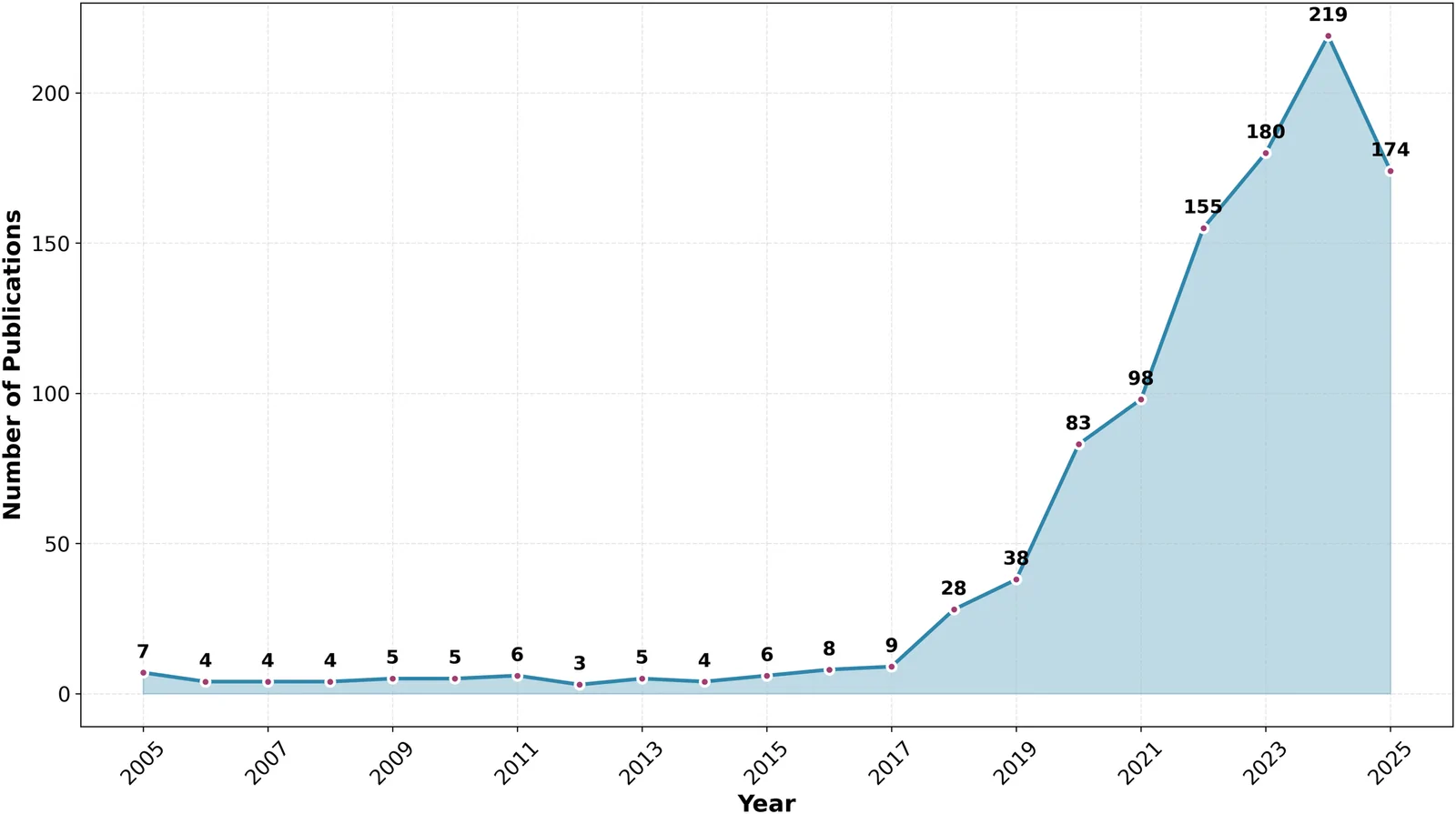
Annual publication trends in AI and ESRD research, 2005–2025 (bibliometric corpus; n = 1,052 records). Three developmental phases are delineated, corresponding to the Stage 1 (rule-based), Stage 2 (data-driven), and Stage 3 (LLM/agent-based) technological trajectory.

Analysis of annual publication output across the 1,052-record bibliometric corpus reveals three distinct phases (Fig 2). The first phase (2005–2017) was one of modest, gradual growth, with annual output rising from 7 publications in 2005 to 9 in 2017 (n = 70 total; 6.7% of the corpus). The second phase (2018–2021) witnessed a pronounced and sustained increase in annual volume, from 28 publications in 2018 to 98 in 2021 (n = 247; 23.5%), a trajectory that coincides with the broad adoption of machine learning and deep learning methods across clinical research domains. The third phase (2022–2025) is tocharacterized by high and sustained annual output, ranging from 155 to 219 publications per year (n = 728; 69.2%), with the research focus progressively shifting from algorithm development and technical validation toward clinical implementation and, most recently, integration of large language models. These three phases align with the Stage 1, Stage 2, and Stage 3 technological trajectory described in Section 5, providing bibliometric corroboration of the developmental arc identified through systematic review.

### 3.2. Geographic and collaboration landscape

**Fig 3:**
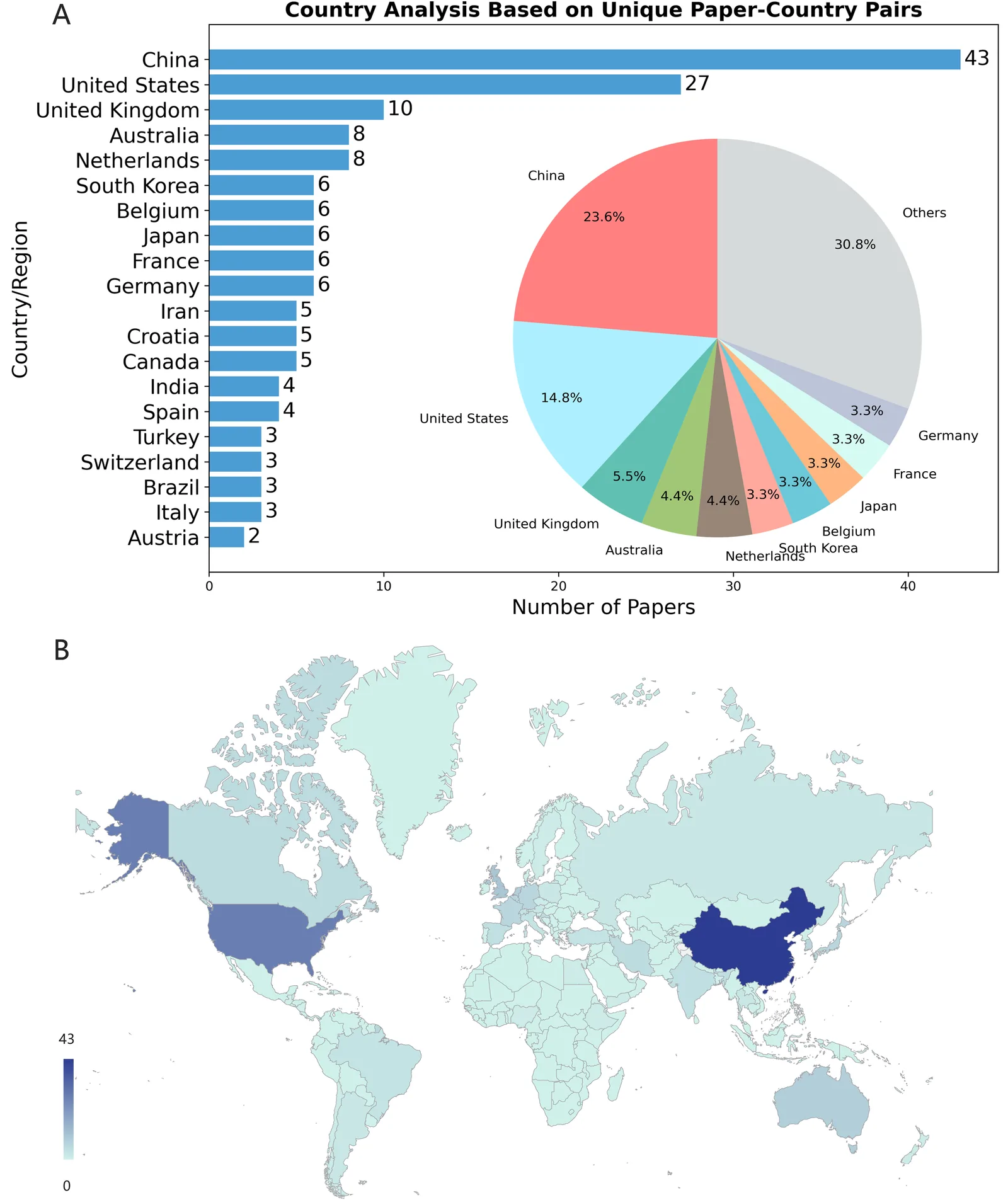
Geographic distribution of AI in ESRD research (systematic review corpus; n = 100 studies). (a) Study count by country affiliation (paper-level counting); (b) Geospatial distribution of contributing countries. Map data: Natural Earth (naturalearthdata.com; public domain).

**Fig 4:**
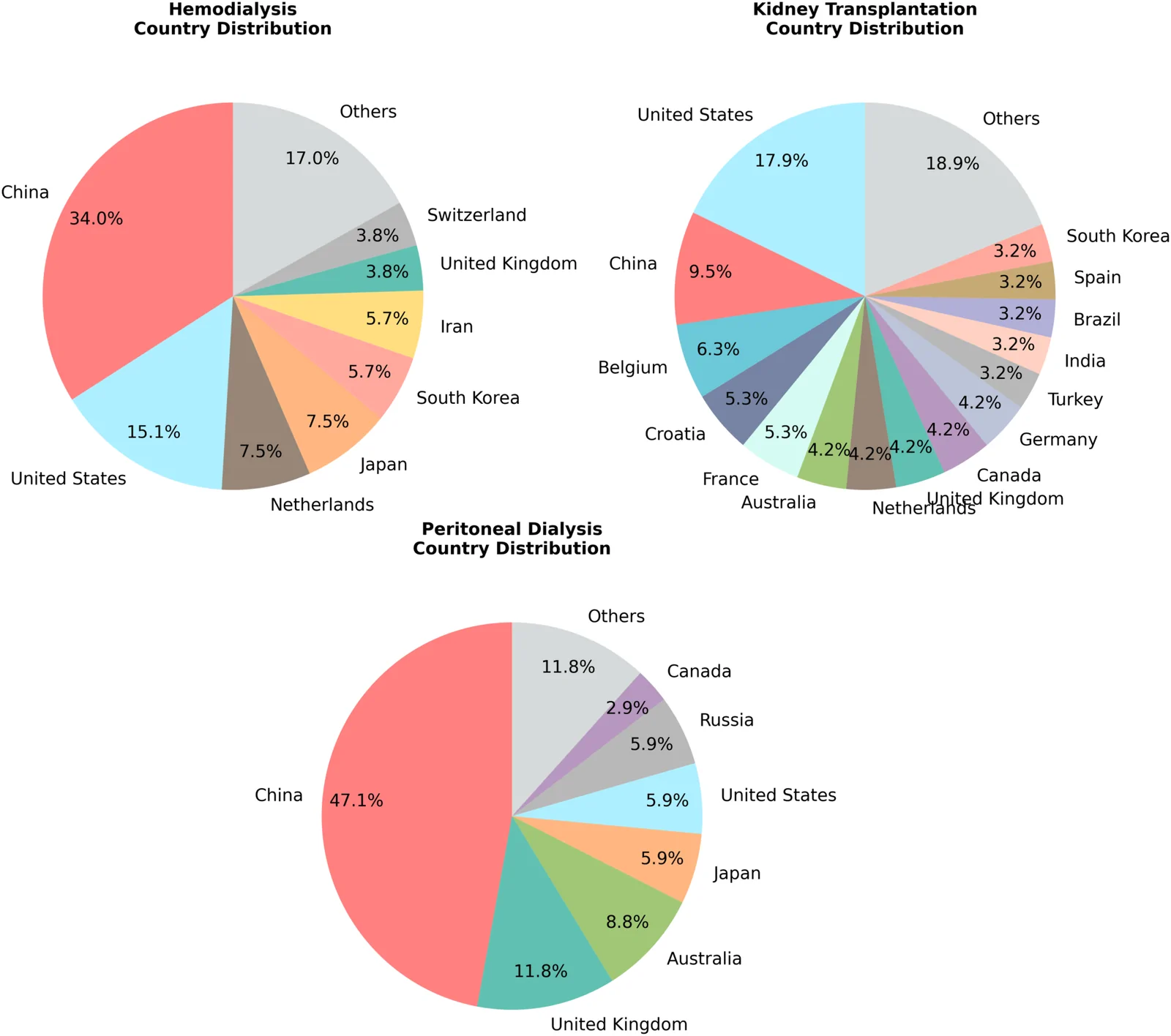
Country distribution across ESRD treatment modalities (systematic review corpus; n = 100 studies). Counts reflect paper-country pairs per modality.

**Fig 5:**
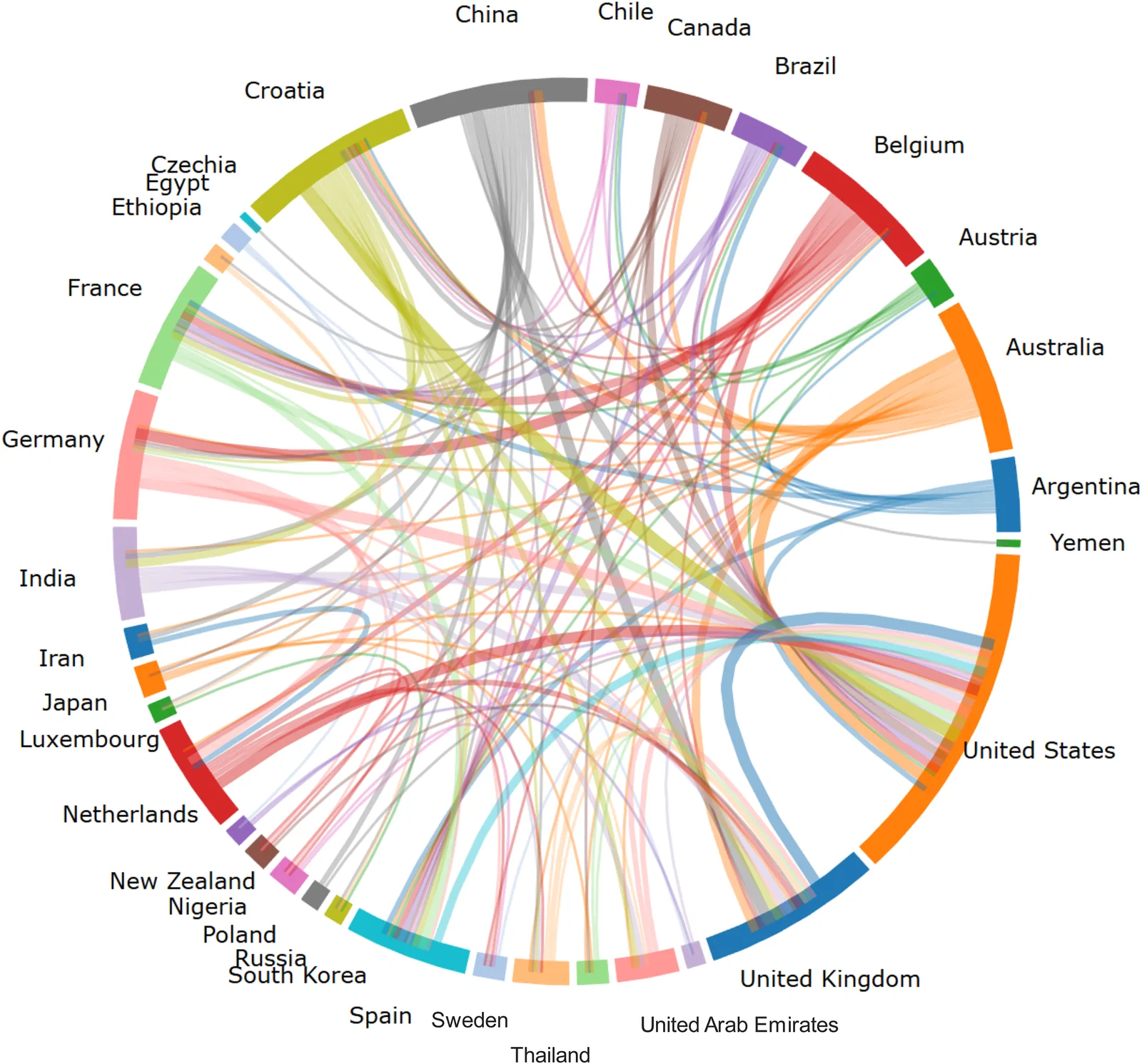
International co-authorship network (systematic review corpus; n = 100 studies). Node size reflects total co-authorship links; edge width reflects shared papers per country pair.

Country affiliation was counted at the paper level (one count per country per paper). Across the 100-study systematic review corpus, 35 countries contributed a total of 182 paper-country pairs (Fig 3), with China (43 studies; 23.6%) and the United States (27; 14.8%) serving as dual centers of global output among the top 10 producers, collectively accounting for approximately 69.2% of all paper-country pairs. Regional specialization by treatment modality is evident (Fig 4): China dominates hemodialysis (33.96% of modality pairs) and peritoneal dialysis (47.06%), while the United States leads kidney transplantation (17.89%) with substantial European co-authorship. The international collaboration network (Fig 5), constructed from 131 co-authorship edges spanning 33 countries, identifies the United States as the primary hub (collaboration weight = 56). This pronounced geographic concentration has direct implications for model generalizability and external validation, discussed in Section 6; full geographic and collaboration data are provided in S2 Text.

### 3.3. Institutional and author profiles

**Fig 6:**
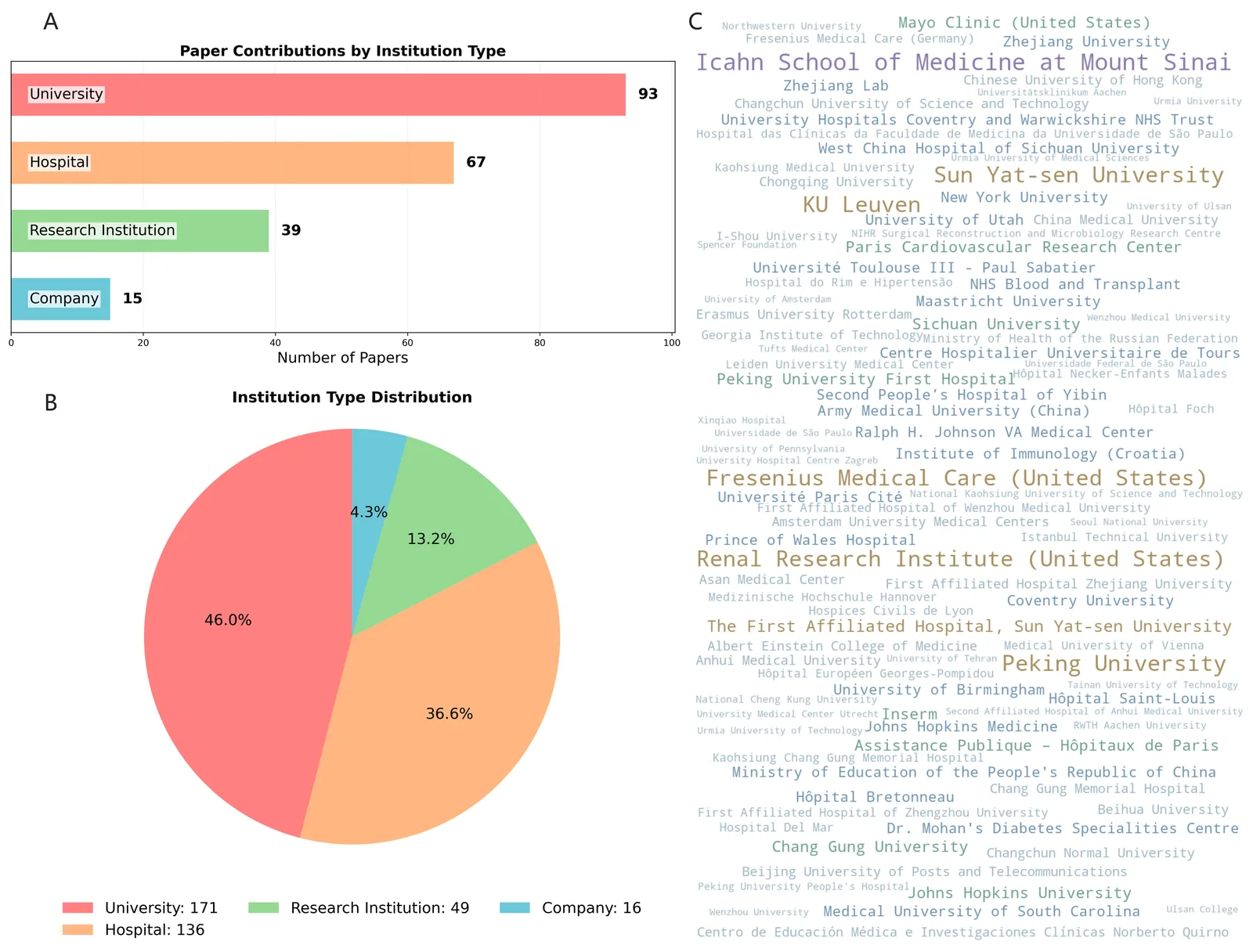
Institutional distribution in AI and ESRD research (systematic review corpus; n = 100 studies). (a) Study count by institution type; (b) Proportion of the corpus with ≥1 author from each institution type; (c) Word cloud of contributing institutions (font size proportional to study count; color indicates institution type). Full institutional and author analyses are provided in S2 Text.

Institutional and author analyses are provided in full in S2 Text. Briefly, 372 distinct institutions contributed across the 100 studies (universities, 45.97%; hospitals, 36.56%; research institutions, 13.17%; industry, 4.30%), with a pronounced core-periphery authorship structure dominated by groups in North America and Europe; researchers from the Southern Hemisphere and Latin America remain substantially under-represented — an important consideration for future efforts to improve data diversity and model equity (Fig 6).

### 3.4. Keyword analysis — evidence for the three-stage framework

**Fig 7:**
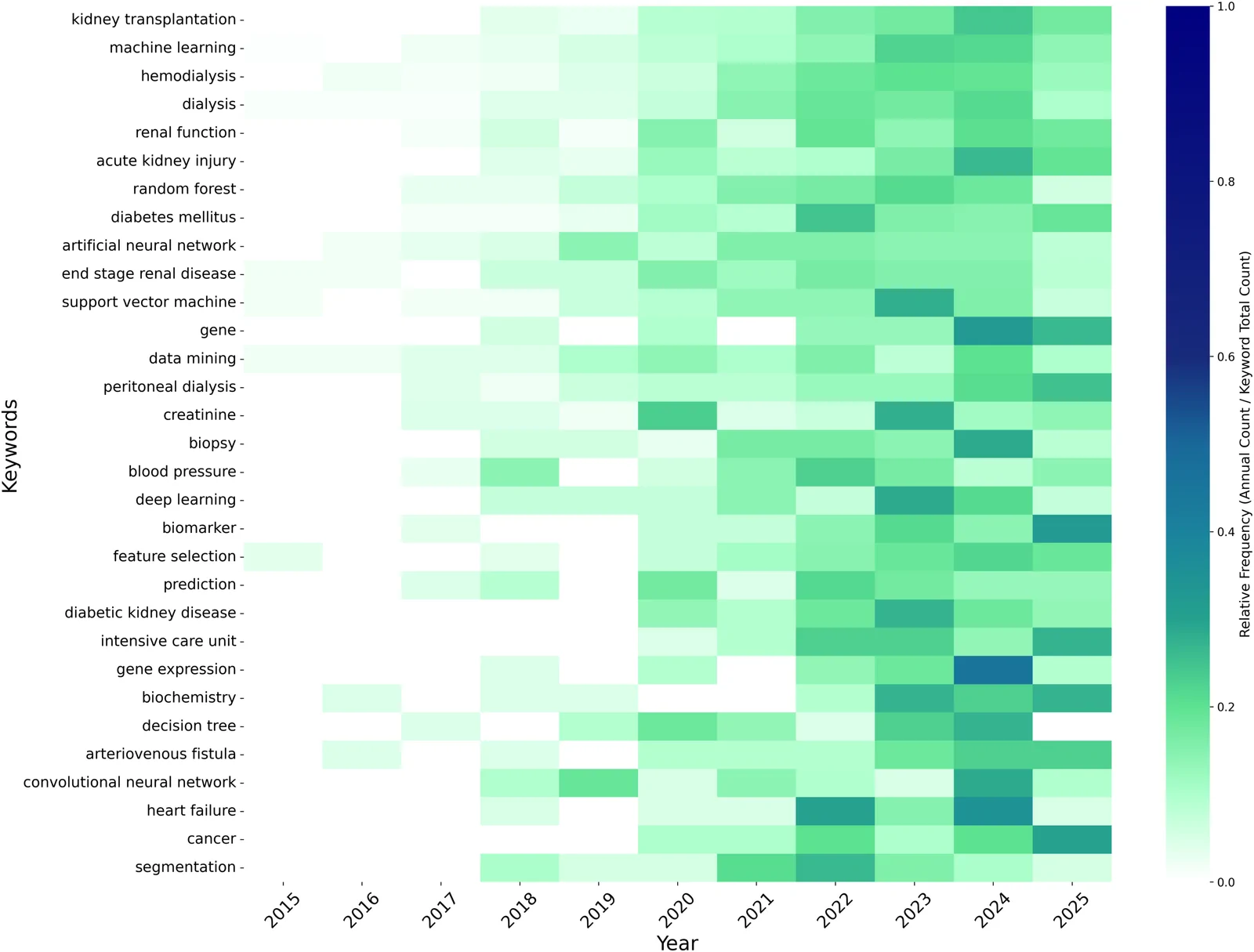
Keyword frequency heatmap (bibliometric corpus; n = 1,052 records; 2015–2025). Top 31 high-frequency keywords; color intensity reflects annual relative frequency (proportion of publications per year carrying each keyword).

Keyword frequency analysis of the 1,052-record bibliometric corpus (2015–2025) provides independent bibliometric corroboration of the three-stage technological trajectory identified through systematic review. The keyword time series heatmap is shown in Fig 7. The top 31 high-frequency keywords were extracted from 1,052 records and their annual relative frequencies (proportion of publications in each year carrying the keyword) were visualised as a heatmap. In the technological dimension, “machine learning” is the dominant technical identifier overall (n = 241 records), with annual relative frequency rising from 0.4% in 2015 to a peak of 22.4% in 2023 before settling at 21.6% in 2024 and 13.7% in 2025, an expansion that directly tracks the Stage 2 surge in data-driven learning. “Random forest” (n = 71) and “support vector machine” (n = 57) exhibit parallel growth trajectories through 2021–2024, consistent with the consolidation of classical machine learning algorithms in ESRD clinical research. “Deep learning” (n = 28) emerged from 2018 onward and reached a relative frequency peak of 28.6% in 2023, with “convolutional neural network” (n = 21) also peaking at 28.6% in 2024, corroborating the progressive adoption of neural architecture approaches that underpins the later Stage 2 research wave. “Artificial neural network” (n = 63) maintains a broader, more distributed trajectory across the full study period, reflecting its longer historical usage. In the clinical application dimension, “kidney transplantation” is the single most frequent keyword in the corpus (n = 334), with annual relative frequency reaching 24.6% in 2024, positioning this modality as the dominant clinical domain. “Hemodialysis” (n = 203) reached a relative frequency of 19.2% in 2024, while “peritoneal dialysis” (n = 48) has risen steadily to 25.0% of annual publications by 2025, consistent with growing interest in home dialysis and remote monitoring. Emerging topical keywords, including “biomarker” (n = 28, reaching 32.1% relative frequency by 2025) and “gene” (n = 53, 32.1% in 2024), suggest progressive enrichment of omics-driven approaches. These keyword trajectories collectively confirm that the annual literature landscape mirrors the Stage 1-to-Stage 3 developmental progression outlined in the analytical framework, providing convergent bibliometric validity for the three-stage review structure.

## 4. Application domains of artificial intelligence in end-stage renal disease patient diagnosis and treatment management

**Fig 8:**
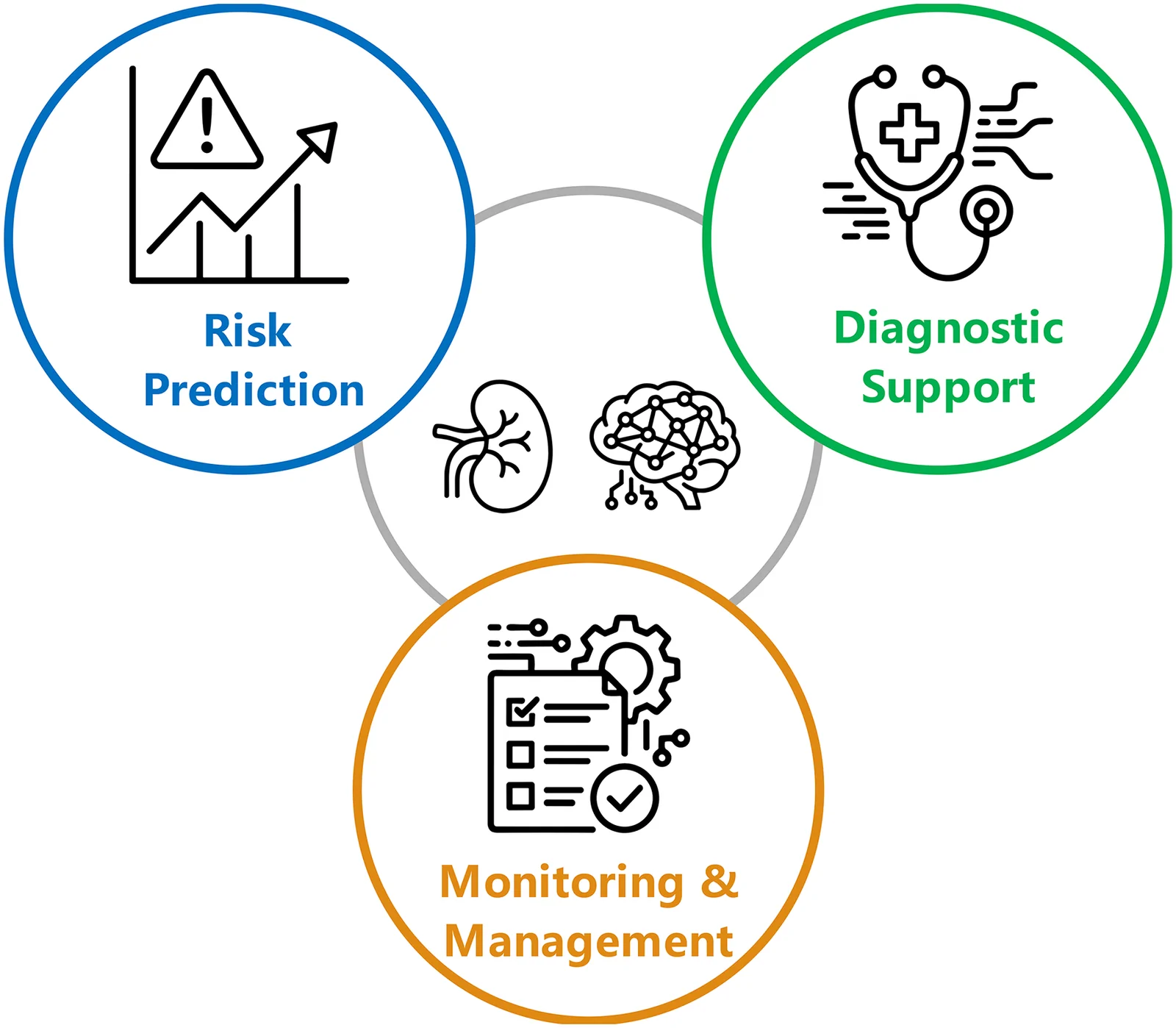
Overview of AI application domains in ESRD care (systematic review corpus; n = 100 studies). Three domains: risk prediction (49.0%), diagnostic support (25.0%), and monitoring and management (26.0%), spanning hemodialysis (n = 40), peritoneal dialysis (n = 21), and kidney transplantation (n = 39).

The diagnosis and treatment management of end-stage renal disease (ESRD) involves multiple renal replacement therapy modalities such as dialysis and kidney transplantation, generating complex multidimensional clinical data accompanied by highly individualized disease trajectories. Traditional medical decision-making based on clinical experience and guidelines has inherent limitations in addressing this complexity, failing to fully utilize the predictive information and diagnostic features embedded within massive datasets. Artificial intelligence (AI) technology, through advanced data processing and pattern recognition capabilities, provides transformative solutions for ESRD patient management. Based on systematic literature analysis, AI applications in the ESRD field can be summarized into three synergistic core domains: risk prediction, diagnostic support, and monitoring and management, as illustrated in Fig 8. These three application domains form an integrated whole, constructing an AI-driven comprehensive precision management framework for ESRD patients throughout the entire disease continuum.

### 4.1. Risk prediction

Risk prediction integrates multidimensional clinical data through machine learning algorithms to achieve precise assessment of patient survival prognosis, complication occurrence, and treatment outcomes. Based on the characteristics of different treatment modalities, AI risk prediction technologies demonstrate distinct application features and technical advantages in the fields of hemodialysis, peritoneal dialysis, and kidney transplantation.

#### 4.1.1. Risk prediction in hemodialysis patients.

As the primary renal replacement therapy modality, hemodialysis has the most mature risk prediction research development in the AI in ESRD field. Machine learning technologies demonstrate significant advantages in survival risk prediction, with attention mechanism-based bidirectional gated recurrent unit (Bi-GRU) networks achieving precise survival prediction through analysis of longitudinal blood pressure records [66], while XGBoost models also perform well in mortality prediction [60]. These deep learning models showed higher predictive performance than traditional scoring systems in the reported comparisons and also captured dynamic characteristics of patient condition changes through temporal analysis.

In the field of complication prediction, AI technology provides important tools for hemodialysis risk management. XGBoost models showed strong performance in cerebral hemorrhage risk prediction through integration of multicenter retrospective data [74], while the BSCWJAYA__KELM model based on boosted machine learning successfully predicts intradialytic hypotension using serum nutritional markers, providing important evidence for clinical preventive interventions [69].

Risk prediction under special clinical circumstances also represents an important research direction. During the COVID-19 pandemic, XGBoost models performed well in predicting multi-timepoint mortality rates in hemodialysis patients following infection [72], providing crucial support for clinical decision-making during the pandemic.

#### 4.1.2. Risk prediction in peritoneal dialysis patients.

As the primary form of home dialysis, peritoneal dialysis risk prediction focuses more on long-term prognosis and complications related to home management, possessing unique clinical value and technical challenges.

Cardiovascular event prediction is the core of risk management in peritoneal dialysis patients. The CVDformer model based on Transformer architecture achieved notable improvements in predicting short-term all-cause mortality and cardiovascular death [49], effectively integrating multidimensional clinical features through attention mechanisms to achieve precise cardiovascular risk assessment. Random forest models also showed strong performance in cardiovascular event prediction [54].

Peritonitis, as a specific complication of peritoneal dialysis, has risk prediction that is crucial for improving dialysis technique survival rates. Random forest models achieved strong performance in predicting peritonitis-related technique failure [47], and machine learning algorithms have been successfully applied to optimize home visit arrangements, guiding peritonitis prevention strategies through generation of risk scores [48].

In hospitalization risk prediction, ensemble learning models based on stacked generalization provide effective tools for extended hospitalization risk assessment [42], contributing to rational allocation of medical resources and optimization of patient management strategies. Blood pressure variability association analysis with clinical outcomes represents an emerging research direction, with ensemble learning models demonstrating good potential in predicting major adverse cardiovascular events and mortality risk through analysis of visit-to-visit blood pressure variability [52].

#### 4.1.3. Risk prediction in kidney transplant patients.

As the optimal treatment modality for ESRD, kidney transplantation risk prediction involves multiple complex aspects including graft survival, delayed graft function, and rejection reactions, with AI technology demonstrating clinical value and technical potential in this field.

In the core field of graft survival prediction, multiple machine learning methods demonstrate strong performance. The L-TOP prediction tool developed by Ali et al. employs deep Cox mixed models, providing an intelligent solution for living donor kidney transplantation outcome prediction [109]. Quinino et al. reported notable improvements in immediate function prediction for deceased donor kidney transplantation using XGBoost algorithms [106], while Yoo et al.’s machine learning-driven virtual biopsy system achieved precise prediction of non-invasive histological lesions through ensemble learning algorithms [117]. In the reported comparisons, these prediction tools exceeded traditional Kidney Donor Profile Index (KDPI) scores in accuracy but also identify key prognostic influencing factors, providing crucial support for optimizing donor allocation and improving patient outcomes.

Bayesian joint models achieved notable advances in dynamically predicting kidney survival in transplant recipients, demonstrating strong performance in multicohort international studies through integration of deep phenotypic data [94]. The advantage of this dynamic prediction method lies in its ability to continuously update risk assessments based on patients’ real-time postoperative monitoring data, providing a crucial tool for precision medicine.

Delayed graft function (DGF) prediction has important value for perioperative management. Random forest classifiers showed strong performance in adult kidney transplant DGF risk prediction [92], while in pediatric kidney transplantation, integrated machine learning models achieve high-precision DGF prediction through comprehensive donor and recipient characteristics [113].

In post-transplant complication prediction, support vector machine models perform well in pneumonia risk prediction for kidney transplant recipients [91], providing important evidence for early postoperative management and preventive interventions.

Human leukocyte antigen (HLA) matching prediction analysis represents a frontier direction in kidney transplantation risk prediction. Through designing multiple biologically relevant feature representation methods to integrate HLA information, gradient boosting models demonstrate good potential in kidney transplant survival time prediction [105].

### 4.2. Diagnostic support

Diagnostic support can enhances the accuracy, efficiency, and standardization of clinical diagnosis through deep learning and machine learning technologies. Based on the characteristics and diagnostic requirements of different treatment modalities, AI diagnostic support technologies demonstrate distinct application advantages in the fields of hemodialysis, peritoneal dialysis, and kidney transplantation.

#### 4.2.1. Diagnostic support in hemodialysis.

Diagnostic support for hemodialysis patients primarily focuses on complication identification, nutritional status assessment, and treatment decision support, with AI technology applications achieving significant improvements in efficiency and precision in these areas.

In the field of fluid overload diagnosis, the artificial intelligence-assisted lung ultrasound (LUS) assessment system developed by Tan et al. employs convolutional neural network (CNN) technology to successfully achieve accurate identification of fluid overload in hemodialysis and peritoneal dialysis patients [44], providing crucial evidence for precise adjustment of dialysis parameters.

In nutritional status assessment, sarcopenia identification receives particular attention. The gender-specific machine learning identification tool developed by Liao et al. showed strong performance in sarcopenia screening for maintenance hemodialysis patients [75], providing precise tools for individualized nutritional management.

#### 4.2.2. Diagnostic support in peritoneal dialysis.

As the primary form of home dialysis, peritoneal dialysis diagnostic support focuses more on rapid diagnosis of peritonitis and rapid assessment of patient characteristics, which is of significant importance for improving home treatment quality.

In the field of rapid peritonitis diagnosis, AI technology has achieved notable progress. Zhang et al. utilized machine learning algorithms to analyse cellular and soluble parameters during peritonitis episodes, successfully defining pathogen-specific local immune fingerprints through random forest methods [39], while Takeuchi et al. developed an “immune fingerprint” system based on immune biomarkers that can precisely predict major pathogen types [38]. These innovative diagnostic tools can shorten diagnosis time and improve treatment timeliness.

In rapid patient characteristic assessment, the innovative method developed by Grunert et al. combining Fourier transform infrared spectroscopy (FTIR) with machine learning achieves rapid and precise assessment of peritoneal dialysis patient characteristics through principal component analysis (PCA) technology [41], providing important support for the development of individualized treatment protocols.

#### 4.2.3. Diagnostic support in kidney transplantation.

As the optimal treatment modality for ESRD, kidney transplantation diagnostic support involves multiple complex aspects including pre-transplant evaluation, post-transplant monitoring, and complication identification. AI technology applications in kidney transplantation diagnostic support demonstrate clinical value and technical potential.

In post-transplant pathological diagnosis, deep learning technology has achieved notable progress. The fully convolutional neural network developed by Marsh et al. performs well in identifying glomerulosclerosis in transplant kidney frozen sections [89]. The RENTAG algorithm designed by Salvi et al. combines deep learning with cellular structure detection to achieve automated quantitative assessment of glomerulosclerosis and tubular atrophy in pre-transplant biopsy samples [95].

Early detection of post-transplant rejection is a key component of transplant management. The computer-aided diagnostic system based on deep learning developed by Shehata et al. utilizes diffusion-weighted magnetic resonance imaging (DW-MRI) data to achieve early detection of acute rejection in kidney transplantation [116]. The automated kidney transplant rejection classification system developed by Ye et al. using multiple instance learning and deep learning technologies performs well in whole slide histopathological image analysis [115].

Multiparametric magnetic resonance imaging combined with deep learning demonstrates unique advantages in transplant diagnosis. The deep hybrid neural network RtNet designed by Zhi et al. utilizes multiparametric magnetic resonance imaging (mpMRI) and clinical biomarkers [103].

Biomarker identification and immune function assessment are also important research directions. Niu et al., through RNA sequencing and advanced machine learning technology, used XGBoost models to identify central biomarkers of post-kidney transplant renal fibrosis [114]. Lee et al. used machine learning-assisted surface-enhanced Raman spectroscopy technology to achieve diagnosis and classification of rejection reactions in kidney transplant recipients through PCA methods [112].

In transplant biopsy lesion assessment, multiple studies demonstrate the advantages of AI technology. Hermsen et al. used convolutional neural networks for quantitative assessment of chronic and inflammatory lesions in kidney transplant biopsy samples [98]. Jacq et al. developed tools using deep learning for automated assessment of total interstitial inflammation and capillaritis in kidney biopsies [104]. Kers et al. used convolutional neural networks for classification of digital pathology images from kidney transplant biopsies [102].

### 4.3. Monitoring and management

Monitoring and management through intelligent algorithms achieve continuous monitoring of patient treatment processes and individualized management optimization, providing real-time, precise data support for clinical decision-making. Based on the characteristics and monitoring requirements of different treatment modalities, AI monitoring and management technologies demonstrate distinct application features and technical advantages in the fields of hemodialysis, peritoneal dialysis, and kidney transplantation.

#### 4.3.1. Monitoring and management in hemodialysis.

In the field of hemodialysis, arteriovenous fistula (AVF), as the lifeline of hemodialysis, requires real-time monitoring of its functional status, which is crucial for patient safety. Expert systems based on Bayesian models perform well in early detection of arteriovenous fistula stenosis [35]. Deep learning technology demonstrates revolutionary potential in vascular access monitoring, with the DeepVAQ model based on convolutional neural networks utilizing photoplethysmography (PPG) sensor data [79]. Acoustic analysis technology has also made important progress in arteriovenous fistula monitoring, with deep learning-based bruit analysis systems achieving good performance in assessing the degree of arteriovenous fistula stenosis [59]. Deep learning models fusing convolutional block attention modules (CBAM) and long short-term memory networks (LSTM) perform even better in arteriovenous fistula blood flow sound signal classification [82]. Cloud computing-based deep learning systems also demonstrate potential in arteriovenous access aneurysm classification [71], while XGBoost models combined with SHapley Additive exPlanations (SHAP) value interpretation achieve strong performance in arteriovenous fistula stenosis risk prediction [86].

Intelligent assessment of dialysis adequacy is a key component in ensuring treatment effectiveness. Random forest models achieve strong performance in predicting dialysis adequacy through analysis of dialysis-related variables [80]. Intelligent management of dry weight is the core component of hemodialysis optimization. Machine learning methods combined with bioelectrical impedance spectroscopy (BIS) data perform well in predicting clinically appropriate dry weight, with XGBoost models achieving 83.26% accuracy [62], and random forest classifiers also making important progress in predicting dry weight changes [73]. Reinforcement learning technology demonstrates unique advantages in dry weight management, with reinforcement learning systems based on double deep Q-networks achieving precise setting and dynamic adjustment of dry weight by learning patients’ individualized response patterns [70].

Intradialytic hypotension, as a common complication, has important clinical value for real-time monitoring and prevention. Recurrent neural network (RNN) models achieve strong performance in real-time prediction of intradialytic hypotension [63], and deep learning models based on pre-dialysis features also perform well in hypotension prediction [76]. XGBoost models combined with cloud computing infrastructure achieve good performance in real-time prediction of intradialytic hypotension [77], and personalized prediction systems achieve strong performance in hypotension prediction by integrating risk factors from previous and current dialysis sessions [83]. The optimized machine learning framework BSWEGWO__KELM based on chronic kidney disease-mineral and bone disorder (CKD-MBD) indicators also demonstrates good potential in intradialytic hypotension prediction [68].

Anemia management is an important component of hemodialysis patient monitoring. Computerized decision support systems (CDS) play important roles in adjusting erythropoietin (EPO) dosage [33], and rule-based clinical decision support systems achieve treatment optimization and standardization in renal anemia management [31]. The artificial intelligence-supported anemia control system (AISACS) utilizes deep learning technology to train experienced physicians’ medication decision data, achieving prevention and management of anemia in maintenance hemodialysis patients [65]. Bayesian network-influence diagram normalized decision support systems fusing ontological knowledge demonstrate important value in dynamic monitoring of clinical and operational risks in hemodialysis patients [32], and rule decision systems based on fuzzy Petri nets (FPNs) combined with Burg autoregressive methods achieve 85.71% accuracy in arteriovenous fistula stenosis assessment [34].

#### 4.3.2. Monitoring and management in peritoneal dialysis.

In the field of peritoneal dialysis, nutritional management and guidance are important components of monitoring and management. Recipe generation tools based on generative pre-trained transformers (GPT) demonstrate potential in nutritional management for peritoneal dialysis patients [18]. These tools can generate individualized nutritional plans based on patients’ nutritional needs and dietary restrictions, improving patients’ nutritional status. Large language model technology has also made important progress in nutritional advice for dialysis patients. ChatGPT shows promising application prospects in providing personalized nutritional advice for hemodialysis patients. Although there is still room for improvement in nutritional analysis accuracy, it performs well in recipe scoring and cooking instructions [120]. Research on the application of large language models in personalized nutritional guidance for chronic kidney disease patients indicates that models like GPT-4 can effectively integrate nutritional knowledge to provide scientific dietary advice for patients [124].

#### 4.3.3. Monitoring and management in kidney transplantation.

In the field of kidney transplantation, patient education and information services are core components of monitoring and management. Large language models such as ChatGPT demonstrate important value in kidney transplant patient education [19]. This technology can provide timely and accurate medical information to patients, improving patients’ health literacy and self-management capabilities. Research on different racial and educational groups’ perception of ChatGPT’s kidney transplantation information quality reveals equity issues in AI technology’s health information dissemination [20]. In intelligent medical documentation management, the Claude 3.5-Sonnet model performs well in generating discharge summaries for patients with renal insufficiency [21]. This study is cited as contextual evidence from the broader renal AI literature rather than as part of the ESRD-specific review corpus, because its population was not restricted to patients with ESRD.

An overview of AI applications across different ESRD treatment modalities is shown in Table 2.

**Table 2:** Overview of AI applications in different ESRD treatment modalities.

Application Domain	Hemodialysis	Peritoneal Dialysis	Kidney Transplantation
Risk Prediction	Survival risk prediction, complication prevention, special situation risk assessment	Cardiovascular event prediction, peritonitis risk assessment, hospitalization risk prediction	Graft survival prediction, delayed graft function prediction, complication prediction
Diagnostic Support	Fluid overload identification, sarcopenia detection, nutritional status assessment	Rapid peritonitis diagnosis, rapid patient characteristic assessment	Automated pathological diagnosis, rejection detection, biomarker identification
Monitoring and Management	Arteriovenous fistula monitoring, dialysis adequacy assessment, dry weight management, hypotension prevention	Individualized nutritional management guidance	Patient education information services, intelligent medical documentation management

Abbreviations: AI, artificial intelligence; ESRD, end-stage renal disease.

### 4.4. Comparative performance across application domains and treatment modalities

The substantial methodological heterogeneity across the 100 included studies, spanning rule-based systems, diverse machine learning algorithms, and large language models applied to three distinct clinical application domains and three treatment modalities—precludes the construction of a single unified performance comparison in which all studies report the same outcome metric. As is standard in systematic reviews of heterogeneous machine learning literature, each study selected its primary performance metric based on the clinical task and outcome type. For binary classification tasks (risk prediction and diagnostic support), the area under the receiver operating characteristic curve (AUC) is the predominant metric; for survival analysis, Harrell’s C-index is the standard; for segmentation and quantification tasks, metrics such as Dice coefficient, IoU, RMSE, and R² are reported; and for LLM applications, task completion accuracy is typically used. No single metric can validly be superimposed across this range of study designs without misrepresenting the original findings. The metric is referred to consistently as AUC throughout both the main text and all tables. Where individual primary studies originally reported this quantity as AUROC or ROC, it has been standardized to AUC, as these notations denote the same quantity (the area under the receiver operating characteristic curve).

Table 3 presents a representative best-performing model for each application domain × treatment modality combination identified within the 100-study corpus, using the primary performance metric reported by each study. For cells in which multiple studies reported AUC, the study with the highest externally validated AUC was selected; where external validation was unavailable, the highest internally validated AUC was used. For cells in which no AUC was reported, the primary metric of the best available study is presented and annotated accordingly. The number of studies contributing to each cell is indicated in parentheses; all underlying studies are listed in full in Table 1. This table should be interpreted as an illustrative summary of performance levels achievable within each domain, not as a meta-analytic pooled estimate.

**Table 3:** Representative best-performing models by application domain and treatment modality (100-study systematic review corpus).

Application Domain	Treatment Modality	Studies in Cell (n)	Representative Best-Performing Study	AI Method	Primary Performance Metric	Additional Reported Metrics	Sample Size	Validation Approach
Risk Prediction	Hemodialysis	14	Li et al. (2023) [74]	XGBoost	AUC: 0.979	ACC: 0.939, SENS: 0.932, SPEC: 0.952, PRE: 0.949, F1: 0.938	393	Internal validation
Risk Prediction	Peritoneal Dialysis	15	Zang et al. (2024) [47]	Random Forest	AUC: 0.916	ACC: 93.7%, SENS: 96.7%, SPEC: 86.5%, PRE: 91.4%, F1: 0.940	508	Internal validation
Risk Prediction	Kidney Transplantation	20	Peng et al. (2020) [91]	Support Vector Machine	AUC: 0.940	SENS: 81.7%, SPEC: 92.0%, PRE: 83.6%	146	External validation
Diagnostic Support	Hemodialysis	3^a^	Liao et al. (2023) [75]	Ensemble Learning	AUC: 0.874	SENS: 77.5%, SPEC: 83.1%, F1: 77.3%	242	Internal validation
Diagnostic Support	Peritoneal Dialysis	5	Zhang et al. (2017) [39]	Random Forest	AUC: 0.993	SENS: 98.5%, SPEC: 92.6%	83	Internal validation (5-fold)
Diagnostic Support	Kidney Transplantation	17	Jacq et al. (2023) [104]	CNN	AUC: 0.92	ACC: 81%, RECALL: 71%, F1: 76%, Dice: 0.84	423	External validation
Monitoring and Management	Hemodialysis	23	Lee et al. (2021) [63]	RNN	AUC: 0.94	—	9,292	Internal validation
Monitoring and Management	Peritoneal Dialysis	1^b^	Jin et al. (2024) [18]	GPT-4	N/A^c^	Prealbumin improvement (p = 0.001, prospective RCT)	35	Prospective clinical trial
Monitoring and Management	Kidney Transplantation	2^b^	Xu et al. (2025) [19]	ChatGPT (GPT-4)	ACC: 92%	—	27	Not formally validated

a The Hemodialysis × Diagnostic Support cell contains only 3 studies (2 rule-based without quantitative performance data; 1 learning-based). Liao et al. (2023) is the sole study in this cell with a reported AUC. ^b^ Cells marked with ^b^ contain very few studies (Peritoneal Dialysis × Monitoring: n = 1; Kidney Transplantation × Monitoring: n = 2), both of which are LLM-based (Stage 3). These cells are not directly comparable to Stage 2 learning-based cells on quantitative metrics; no conventional classification performance metrics were reported. ^c^ Jin et al. (2024) conducted a prospective randomised controlled trial evaluating GPT-4-generated personalized nutritional plans for peritoneal dialysis patients. The primary outcome was a significant improvement in serum prealbumin at 12 weeks (p = 0.001), rather than a classification accuracy metric. This reflects the shift in evaluation paradigm for LLM-based interventions from retrospective algorithmic benchmarking toward prospective clinical outcome measurement.

N/A denotes not applicable: the study did not report a conventional classification performance metric (e.g., AUC) because its primary outcome was a prospective clinical endpoint rather than an algorithmic classification metric (see footnote c). A dash (–) denotes a value not reported in the original study.

Abbreviations: AI, artificial intelligence; AUC, area under the receiver operating characteristic curve; ACC, accuracy; SENS, sensitivity; SPEC, specificity; PRE, precision; RECALL, recall; F1, F1 score; Dice, Dice similarity coefficient; RCT, randomized controlled trial; CNN, convolutional neural network; RNN, recurrent neural network; XGBoost, extreme gradient boosting; GPT, generative pre-trained transformer.

Across all three application domains, Stage 2 learning-based models consistently achieved AUC values in the 0.87–0.99 range, with the strongest performance observed in peritoneal dialysis diagnostic support (Zhang et al. AUC 0.993 for pathogen-specific peritonitis diagnosis) and hemodialysis risk prediction (Li et al., AUC 0.979 for cerebral hemorrhage prediction). Kidney transplantation risk prediction, which is characterized by greater patient and donor heterogeneity and longer outcome horizons, yielded somewhat more modest but clinically useful AUC values (0.75–0.94 across the 20 studies in this cell), with Peng et al.’s externally validated support vector machine (SVM) model (AUC 0.940) representing the strongest externally validated result. Diagnostic support studies in kidney transplantation relied predominantly on deep learning applied to histopathology images, with Jacq et al.’s CNN achieving AUC 0.92 on an independent external test set. The monitoring and management domain, covering real-time clinical decision support, vascular access management, and dry weight optimization, achieved the highest AUC among hemodialysis monitoring models (Lee et al. RNN, AUC 0.94 for intradialytic hypotension prediction, n = 9,292). The two monitoring cells with the fewest studies (peritoneal dialysis, n = 1; kidney transplantation, n = 2) are dominated by Stage 3 LLM-based applications that use evaluation frameworks incompatible with AUC-based comparison, underscoring the emerging nature of LLM deployment in these modality-specific monitoring contexts.

## 5. Evolutionary analysis of AI technologies in ESRD patient diagnosis and treatment management: from tools to cognitive systems

**Fig 9:**
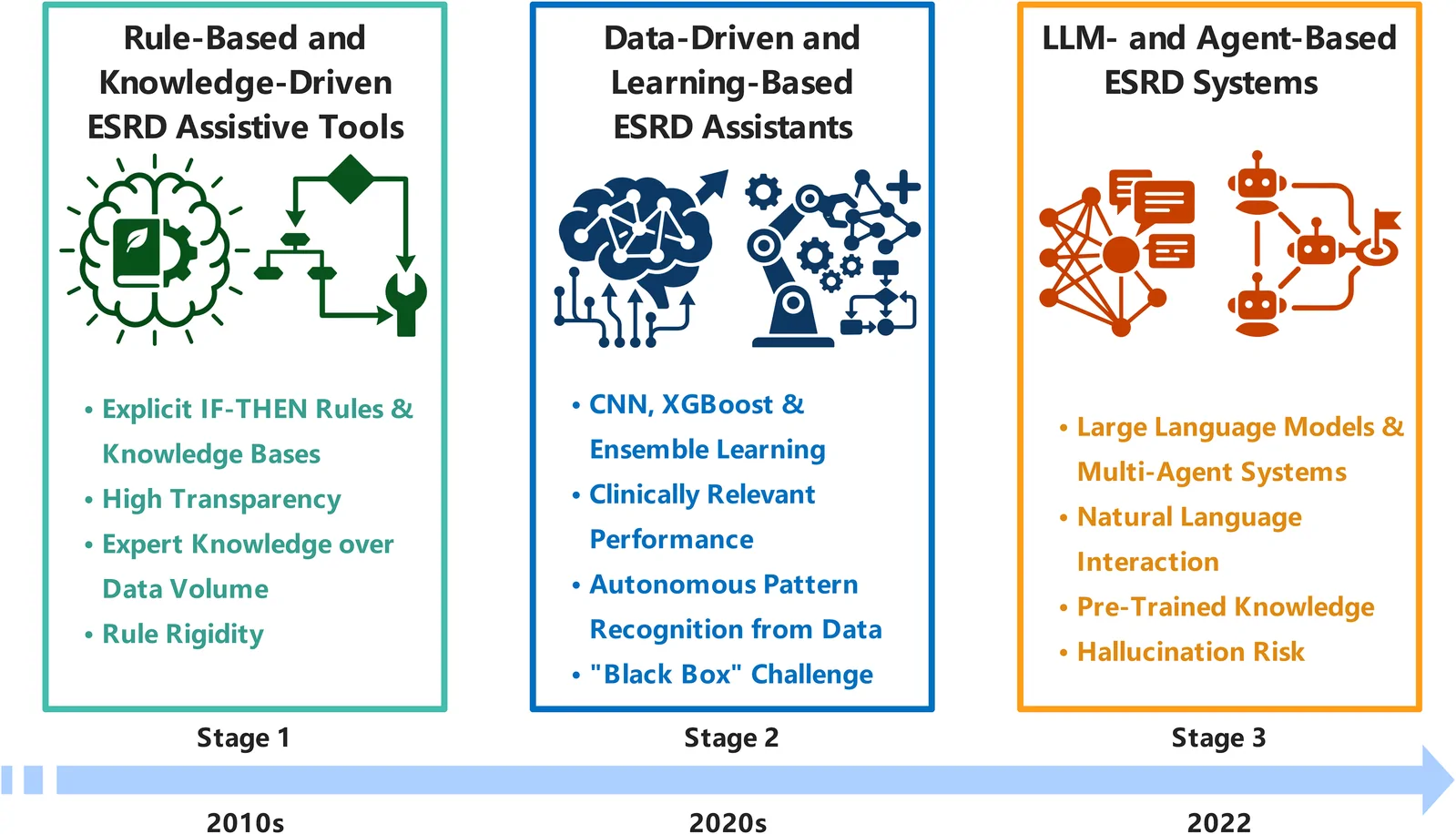
Three-stage developmental framework for AI in ESRD care (systematic review corpus; n = 100 studies). Stage 1 (1980s–2010s): rule-based and knowledge-driven assistive tools (n = 10); high interpretability but limited scalability. Stage 2 (2010s–2020s): data-driven learning systems (n = 83); AUC 0.80–0.90 with reduced interpretability. Stage 3 (2022–present): an emerging research direction encompassing LLM- and agent-based cognitive systems (n = 7); broad task versatility but limited formal validation in ESRD-specific populations; findings are preliminary. Stage assignment criteria (Section 2.7); inter-rater agreement on stage assignment κ = 0.90 (Section 2.7).

With the rapid advancement of artificial intelligence technology, its application in ESRD diagnosis and treatment management has undergone profound transformation across three distinct evolutionary stages. All analyses in this section are grounded in the 100 ESRD-focused studies identified through the PRISMA screening process described in Section 3. This section systematically examines each stage across six critical dimensions—literature contributions, sample characteristics, methodological approaches, performance metrics, validation strategies, and interpretability features—enabling direct comparison of research characteristics across developmental stages and providing insight into the intrinsic patterns driving AI advancement toward clinical translation in ESRD care, as illustrated in Fig 9. The complete stage-wise bibliography is summarized in Table 1.

### 5.1. Stage 1: rule-based and knowledge-driven assistive tools

#### 5.1.1. Literature overview and clinical applications.

The foundational era of AI in ESRD management emerged from the broader development of artificial intelligence, formally initiated at the Dartmouth College conference in 1956 [125]. Expert systems, introduced by Edward Feigenbaum in 1965 [126], became the mainstream AI technology in healthcare during the 1980s-2010s period [127]. These systems utilized explicit rules and structured knowledge bases, employing “if-then” logical rules and symbolic reasoning to simulate nephrology specialists’ decision-making processes [128]. Early healthcare AI applications from the 1970s, including landmark systems such as Stanford’s MYCIN for infection diagnosis [129], Pittsburgh’s INTERNIST-1 for symptom-based diagnosis [130], and Massachusetts’s DXplain for diagnostic support [131], established the foundation for clinical decision support. In ESRD, representative applications included Raghavan et al.’s rule engine for dialysis treatment decisions [29], computerized anemia management systems [31], and monitoring systems for complications such as arteriovenous fistula stenosis [34]. While demonstrating practical value in standardized scenarios, these systems showed limitations when addressing the complexity and individual variability inherent in ESRD patient management [132].

#### 5.1.2. Sample characteristics and scale analysis.

Rule-based systems typically operated on relatively modest datasets, reflecting their reliance on expert knowledge rather than large-scale data learning. Analysis of representative studies reveals sample sizes ranging from 42 patients (Chen et al.’s fuzzy logic system) [34] to 8,941 patients (Miskulin et al.’s anemia management system) [33], with most studies involving fewer than 1,000 patients. The European Renal Association–European Dialysis and Transplant Association (ERA-EDTA) study by Geddes et al. represented an exception with 2,310 patients across 793 dialysis centers in 37 European countries [30], demonstrating the potential for large-scale rule-based applications. These modest sample requirements aligned with the knowledge-engineering approach that prioritized domain expertise over data volume.

#### 5.1.3. Methodological approaches.

Rule-based systems in ESRD employed diverse knowledge representation and reasoning methodologies, as summarized in Table 4. The predominant approaches included classical rule-based systems, Bayesian networks, and fuzzy logic systems. Classical rule-based systems [28,29,31,33,37] provided transparent “if-then” decision pathways, while Bayesian networks [32,35] enabled probabilistic reasoning under uncertainty. Fuzzy logic systems [34,36] addressed the inherent imprecision in medical decision-making, particularly valuable in complex scenarios such as vascular access assessment.

**Table 4:** Methodological distribution in rule-based and knowledge-driven systems.

Method Category	Representative Applications	Key Advantages
Rule-based Systems	Treatment decision support, anemia management	High transparency, explicit reasoning paths
Bayesian Networks	Vascular access monitoring, risk assessment	Probabilistic reasoning, uncertainty handling
Fuzzy Logic	Stenosis detection, transplant decisions	Handles imprecision, continuous reasoning
Modified Clinical Scores	Survival prediction	Clinical acceptability, validated metrics

Note: This table contains no abbreviated terms; all method categories and descriptors are given in full.

#### 5.1.4. Performance assessment.

Rule-based systems demonstrated stable but limited performance characteristics. Quantitative performance ranged from 84.2% precision for survival prediction (Geddes et al.) [30] to 100% sensitivity and specificity for treatment decision support (Niazkhani et al.) [37]. The fuzzy logic system by Improta et al. achieved balanced performance with 93% accuracy, 90% sensitivity, and 94% specificity [36]. However, many systems lacked comprehensive performance evaluation, with several studies reporting only qualitative outcomes or process improvements rather than predictive accuracy metrics.

#### 5.1.5. Validation strategies.

Validation approaches in rule-based systems were predominantly limited. Among the 10 Stage 1 studies included in this review, 5 (50.0%) employed no formal validation protocol; 4 relied on basic internal validation via hold-out methods or historical comparisons; and one study, the ERA-EDTA cohort by Geddes et al., undertook rigorous external validation across multiple European dialysis centers [30]. This limited validation landscape reflects the knowledge-engineering paradigm’s emphasis on domain expert consensus over empirical performance evaluation, a characteristic limitation of early AI systems in clinical settings.

#### 5.1.6. Interpretability characteristics.

Rule-based systems exhibited high interpretability, with all systems providing transparent decision pathways. The explicit “if-then” rule structures enabled clinicians to understand, challenge, and modify system recommendations. Bayesian networks enhanced interpretability through visualization of causal relationships and probabilistic dependencies. Fuzzy logic systems provided intuitive reasoning through linguistic variables and membership functions. This high interpretability represented a critical strength, establishing clinical trust and regulatory compliance, though at the cost of performance limitations in complex scenarios.

### 5.2. Stage 2: data-driven and learning-based assistants

#### 5.2.1. Literature overview and technological paradigm.

The second stage marked a fundamental paradigm shift from human-coded rules to data-driven pattern recognition. Beginning in the early 21st century, AI experienced a resurgence driven by advances in deep learning, transforming systems from passive tools to intelligent assistants that augmented human expertise [133]. Machine learning enabled autonomous pattern recognition from massive datasets, contrasting sharply with manual rule design approaches. Deep learning architectures utilized artificial neural networks mimicking brain connectivity, with backpropagation algorithms improving training efficiency [134]. This evolution was facilitated by exponential growth in computational power, particularly graphics processing unit (GPU) acceleration, proliferation of electronic health records, cloud computing infrastructure, and open-source libraries [135]. In ESRD, these advances enabled autonomous learning of disease and treatment patterns from clinical data, providing unprecedented possibilities for imaging diagnosis, prognostic assessment, and personalized treatment [136]. Applications spanned all three treatment modalities, with peritoneal dialysis focusing on cardiovascular risk prediction and peritonitis detection, hemodialysis emphasizing real-time monitoring and adverse event prediction, and kidney transplantation advancing in prognostic modeling and pathological diagnosis.

#### 5.2.2. Sample scale analysis.

Learning-based methods demonstrated larger sample requirements, reflecting their data-dependent nature. Sample sizes exhibited remarkable heterogeneity spanning five orders of magnitude (17–277,316 cases), with a mean of 12,508 cases (median 641 cases). Risk prediction studies required the largest datasets (mean 20,714 cases), while diagnostic support studies operated with smaller, more curated samples (mean 408 cases). This substantial increase in data requirements compared to rule-based systems highlighted the fundamental shift toward empirical learning paradigms.

#### 5.2.3. Methodological landscape.

**Fig 10:**
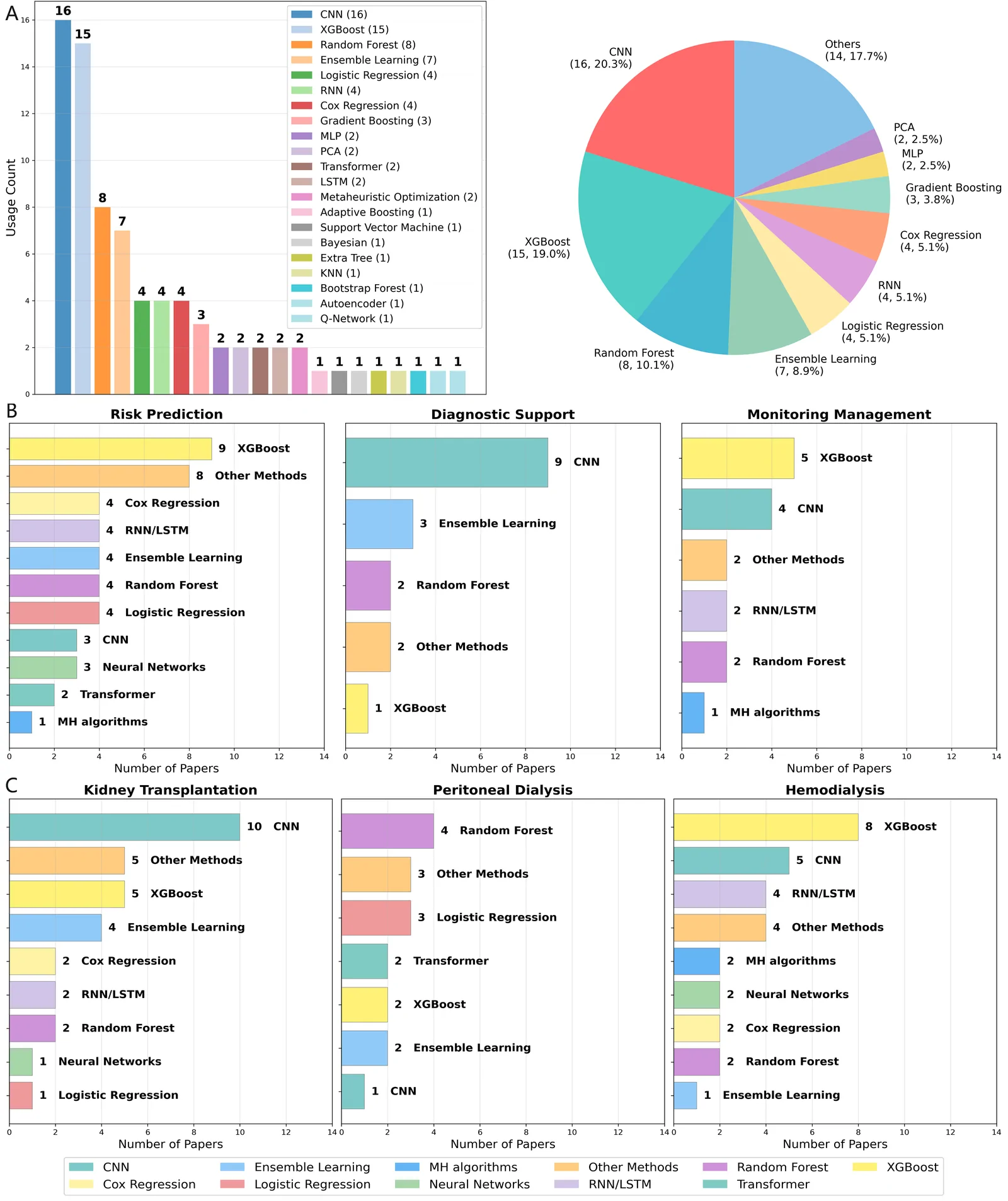
AI method distribution in data-driven and learning-based studies (Stage 2; n = 83, systematic review corpus). (a) Overall distribution; (b) by application domain; (c) by treatment modality.

Data-driven approaches demonstrated remarkable methodological diversity, as shown in Fig 10-a. CNN emerged as the most frequent method with 16 applications (20.3%), followed closely by XGBoost with 15 applications (19.0%). This distribution reflected ESRD clinical practice’s dual demands for high performance and relative interpretability. The methodological landscape varied across application domains (Fig 10-b): risk prediction was dominated by XGBoost (19.6%) and ensemble methods (8.7%), diagnostic support featured CNN dominance (52.9%), while monitoring and management showed balanced distribution between XGBoost (31.2%) and CNN (25.0%).

Treatment modality analysis revealed distinct technological preferences (Fig 10-c): kidney transplantation favored deep learning with CNN comprising 31.2% of studies, peritoneal dialysis demonstrated balanced distribution with random forest leading at 23.5%, and hemodialysis showed strong XGBoost preference at 26.7%. This differentiation reflected the alignment between clinical needs and technological capabilities across treatment contexts.

#### 5.2.4. Performance characteristics.

**Fig 11:**
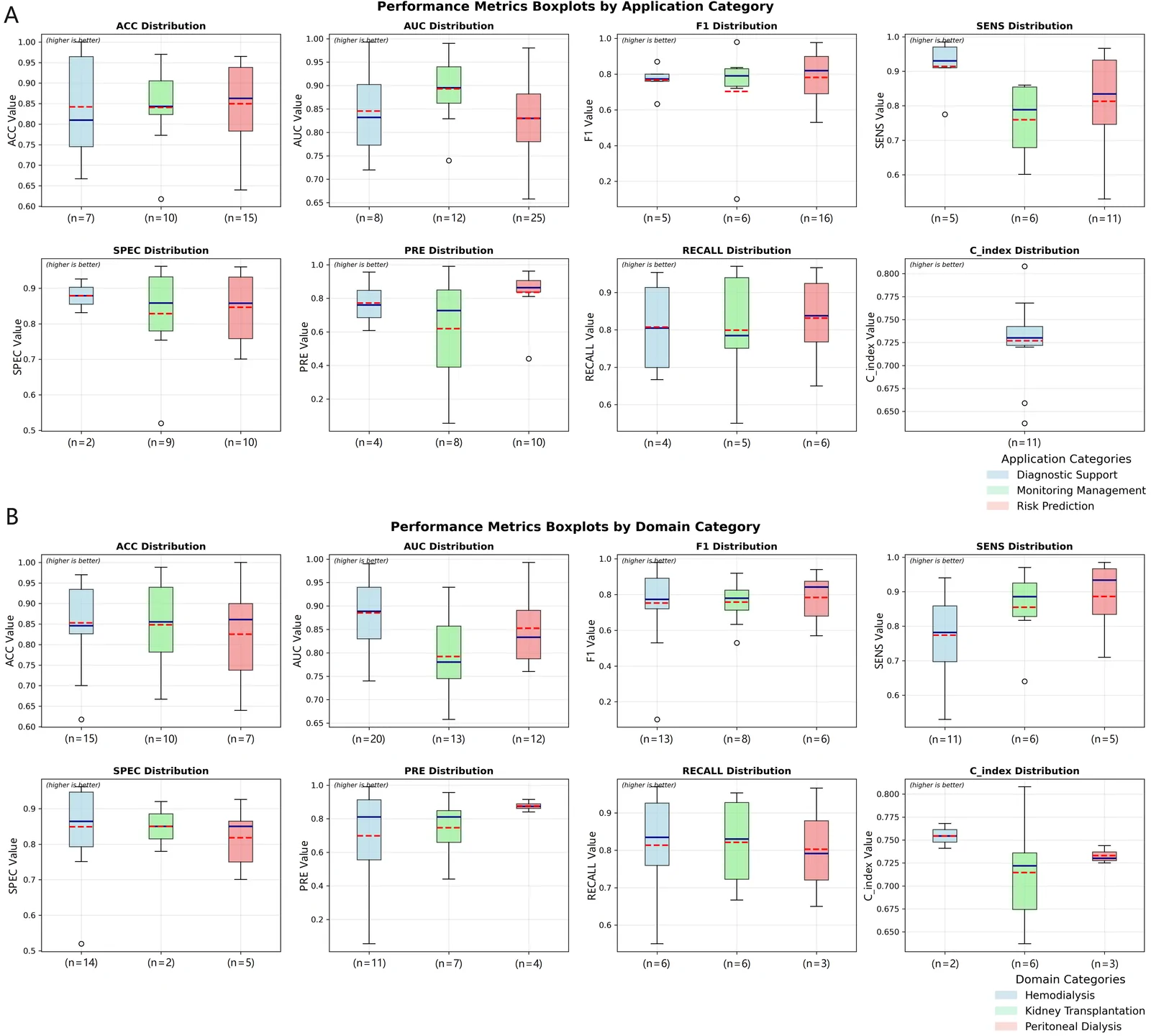
Performance characteristics of data-driven and learning-based AI models (Stage 2; n = 83, systematic review corpus). (a) By application domain; (b) by treatment modality.

Learning-based methods achieved substantial performance improvements over rule-based systems, with most studies demonstrating AUC values in the 0.80-0.90 range. Performance analysis across application domains revealed distinct patterns (Fig 11-a): risk prediction showed the most stable performance (AUC 0.830 ± 0.082, accuracy 0.850 ± 0.106), monitoring and management achieved strong AUC performance (0.893 ± 0.069) with greater precision variability, and diagnostic support demonstrated outstanding sensitivity (0.914 ± 0.083).

Cross-modality analysis (Fig 11-b) revealed treatment-specific performance characteristics: hemodialysis benefited from standardized procedures and rich monitoring data (AUC 0.885 ± 0.070), kidney transplantation performed well in survival analysis indicators (C-index 0.715 ± 0.061), and peritoneal dialysis demonstrated strong sensitivity (0.886 ± 0.114). The consistent pattern of higher sensitivity than specificity across all domains reflected the medical AI principle of “better to misdiagnose than to miss a diagnosis.”

#### 5.2.5. Validation maturity.

Learning-based studies demonstrated enhanced validation rigor, with 97.6% (81/83) employing at least one formal validation protocol. Internal validation, comprising cross-validation (k-fold and 10-fold designs), hold-out splitting, and leave-one-out approaches, was the predominant strategy (86.7%; 72/83). For the purposes of this review, external validation was defined as model evaluation on a dataset that was collected independently of the training data. This encompasses three configurations: (a) a geographically or institutionally distinct cohort not involved in model development; (b) a publicly available benchmark dataset applied after model training was complete; or (c) a prospective or temporally separate dataset from a different time period at the same or a different site. Temporal splits within the same institutional dataset, where training and test subsets are drawn from the same source under the same protocol, were classified as internal validation. On this basis, external validation was employed in 26.5% of studies (22/83), either as a standalone strategy or in combination with internal methods. Bootstrap resampling was documented in 7.2% of studies (6/83). Notably, no study among this cohort reported prospective clinical validation as part of the primary evaluation design, highlighting a persistent gap between computational performance and real-world clinical deployment readiness. This evolution toward rigorous validation protocols reflects the growing methodological sophistication of the machine learning paradigm and the field’s increasing emphasis on model generalizability.

#### 5.2.6. Interpretability evolution.

Learning-based methods experienced significant interpretability challenges due to model complexity. Post-hoc explanation became the predominant approach, with SHapley Additive exPlanations (SHAP) emerging as the dominant interpretability framework in 45.0% (37/83) of studies providing explanations. Local Interpretable Model-agnostic Explanations (LIME) and feature importance rankings supplemented interpretability efforts. However, 38.8% (32/83) of studies provided no interpretability analysis, highlighting the “black box” challenge. Advanced deep learning models faced particular interpretability constraints, leading to the development of attention mechanisms and visualization techniques for CNN-based systems.

### 5.3. Stage 3: Large language model- and agent-based systems — an emerging research direction

#### 5.3.1. Literature overview and cognitive intelligence transition.

The third stage is best characterized as an emerging research direction rather than an established phase comparable to Stage 1 or Stage 2. Unlike rule-based and learning-based systems, which are represented by 10 and 83 ESRD studies respectively, only 7 of the 100 included ESRD studies employed large language model- or agent-based approaches—and most of these (five of the seven) lacked a formal validation protocol. The characterization of Stage 3 as a distinct stage in this review reflects a directional trajectory supported by the broader AI literature rather than a body of ESRD-specific evidence of the size or maturity seen in earlier stages. Findings presented in Sections 5.3.2–5.3.6 should accordingly be interpreted as preliminary observations on an active research frontier, not as settled conclusions.

To avoid ambiguity, a clear distinction is maintained throughout this section between two categories of evidence. The first comprises the seven ESRD-specific studies that form part of the 100-study review corpus (Table 1); all seven apply general-purpose or domain-adapted large language models to patients with ESRD undergoing hemodialysis, peritoneal dialysis, or kidney transplantation. The second comprises a small number of multi-agent and retrieval-augmented systems drawn from the broader biomedical artificial intelligence literature (for example, ColaCare, MDAgents, MMedAgent, and MedGen), which were not developed in ESRD-specific populations and are therefore not part of the review corpus. These broader systems are cited solely to contextualize the methodological trajectory of cognitive AI; they are not counted among the 100 included studies and are not used to support any ESRD-specific quantitative claim.

Beginning around 2020, large language models such as GPT-3 demonstrated natural language processing capabilities with potential relevance to clinical tasks [137]. Built upon Transformer architectures with self-attention mechanisms [138], these models can process unstructured clinical text, navigate complex medical terminology, and support multi-turn dialogue. Representative biomedical language models include BioBERT [139], ClinicalBERT [140], BioMedLM [141], GatorTron [142], and Med-PaLM 2 [143], each pre-trained on large-scale biomedical corpora. Agent systems represent a further development, extending language model capabilities with persistent memory, autonomous task planning, external tool use, and retrieval-augmented generation [144–147]—features that could, in principle, support continuous patient monitoring and multi-step clinical decision support [148]. However, in the ESRD context, direct empirical evidence for these capabilities remains limited at the time of this review.

#### 5.3.2. Sample requirements and data paradigm.

The seven ESRD-specific Stage 3 studies operated with diverse sample paradigms. These large language model applications generally required minimal task-specific training data (mean 209 cases), instead leveraging pre-trained knowledge from massive text corpora, and several conversational applications relied on small evaluation sets focused on response quality rather than training volume. In the broader biomedical AI literature, agent systems show more varied data requirements; for example, the ColaCare framework was evaluated on large-scale electronic health record (EHR) data (the MIMIC-IV dataset) [22], although that system was not developed in an ESRD-specific population and is cited here only for context. Taken together, these patterns reflect a paradigm shift from data-centric toward knowledge-centric learning.

#### 5.3.3. Advanced methodological approaches.

All seven ESRD-specific Stage 3 studies employed a general-purpose or domain-adapted large language model as the primary AI component: six applied general-purpose conversational or multimodal models [18–20,120–122] (ChatGPT or GPT-4/GPT-4V) and one applied a domain-adapted biomedical language model (exKidneyBERT [123]). None of the seven ESRD-specific studies implemented a multi-agent or retrieval-augmented generation architecture; to date such designs have been reported only in the broader biomedical AI literature, which we cite for methodological context rather than as ESRD-specific evidence. This distribution should be interpreted with caution given the small study count and is reported only as a descriptive characterization of the current ESRD-specific literature. Table 5 summarizes the principal methodological categories of large language model- and agent-based systems represented in the wider biomedical AI literature that contextualizes these ESRD studies.

**Table 5:** Methodological categories in large language model and agent-based systems.

Method Category	Representative Models	Key Capabilities
Large Language Models	ChatGPT, GPT-4, Claude, exKidneyBERT	Natural language interaction, reasoning
Multi-Agent Systems	ColaCare, MDAgents, MMedAgent	Collaborative intelligence, autonomous action
Enhanced LLMs (RAG/CoT)	GPT-4 + RAG, Chain-of-Thought systems	Knowledge retrieval, structured reasoning
Specialized Medical Models	DeepSeek-r1, OncoLLM combinations	Domain-specific optimization

Abbreviations: LLM, large language model; RAG, retrieval-augmented generation; CoT, chain-of-thought; GPT, generative pre-trained transformer; BERT, bidirectional encoder representations from transformers. The entries in the “Representative Models” column (e.g., ChatGPT, Claude, exKidneyBERT, ColaCare, MDAgents, MMedAgent, DeepSeek-r1, OncoLLM) are proper names of specific models or systems rather than abbreviations.

The representative models listed are drawn from the broader biomedical AI literature and are included to illustrate methodological categories. Not all were developed or evaluated in ESRD-specific populations; multi-agent and retrieval-augmented systems are not represented among the seven ESRD-specific Stage 3 studies in the review corpus (Table 1).

#### 5.3.4. Performance and versatility assessment.

The seven ESRD-specific Stage 3 studies showed broad task versatility alongside variable, task-dependent performance. Among ESRD applications, ChatGPT reached 92% accuracy in kidney transplant patient education [19], GPT-4V reached 83.33% in transplant clinical case assessment [121], and ChatGPT reached 95.5% in transplant treatment decision support [122]. For methodological context, systems from the broader biomedical AI literature have reported strong results on general medical tasks, for example, the ColaCare multi-agent framework reported an area under the receiver operating characteristic curve (AUC) of 88.80 ± 1.31 for mortality prediction on general EHR data [22], and the MDAgents framework achieved the best performance on 7 of 10 general medical benchmark tasks [23], although these systems were not developed or evaluated in ESRD-specific populations. Across both ESRD-specific and contextual evidence, the recurring theme is a shift from task-specific optimization toward general-purpose adaptability; in the ESRD setting, however, this remains an early and as yet largely unvalidated direction.

#### 5.3.5. Validation approaches and real-world integration.

Advanced systems employed diverse validation methodologies reflecting their novel characteristics. Traditional validation metrics became insufficient for conversational and autonomous systems. New evaluation frameworks emerged focusing on response quality, clinical utility, and real-world applicability. Prospective clinical validation became more prominent, exemplified by Jin et al.’s clinical trial demonstrating significant prealbumin improvement (p = 0.001) [18]. However, five of the seven ESRD-specific Stage 3 studies reported no formal validation procedure—one (exKidneyBERT [123]) used internal hold-out validation, and one (Jin et al. [18]) was evaluated in a prospective pilot randomized controlled trial—reflecting the early developmental stage and methodological challenges in evaluating cognitive AI systems.

#### 5.3.6. Interpretability renaissance.

Stage 3 systems demonstrated a renaissance in interpretability through natural language explanations and structured reasoning. Large language models provided intuitive explanations through conversational interfaces, though suffering from potential “hallucination” issues. Agent systems enhanced interpretability through explicit reasoning chains, tool invocation transparency, and decision audit trails. Chain-of-thought reasoning and structured prompting revealed intermediate reasoning steps, providing more transparent decision processes. This evolution toward “moderate interpretability-excellent performance” represented a potential resolution to the long-standing interpretability-performance trade-off.

### 5.4. Cross-stage comparative analysis and developmental patterns

**Fig 12:**
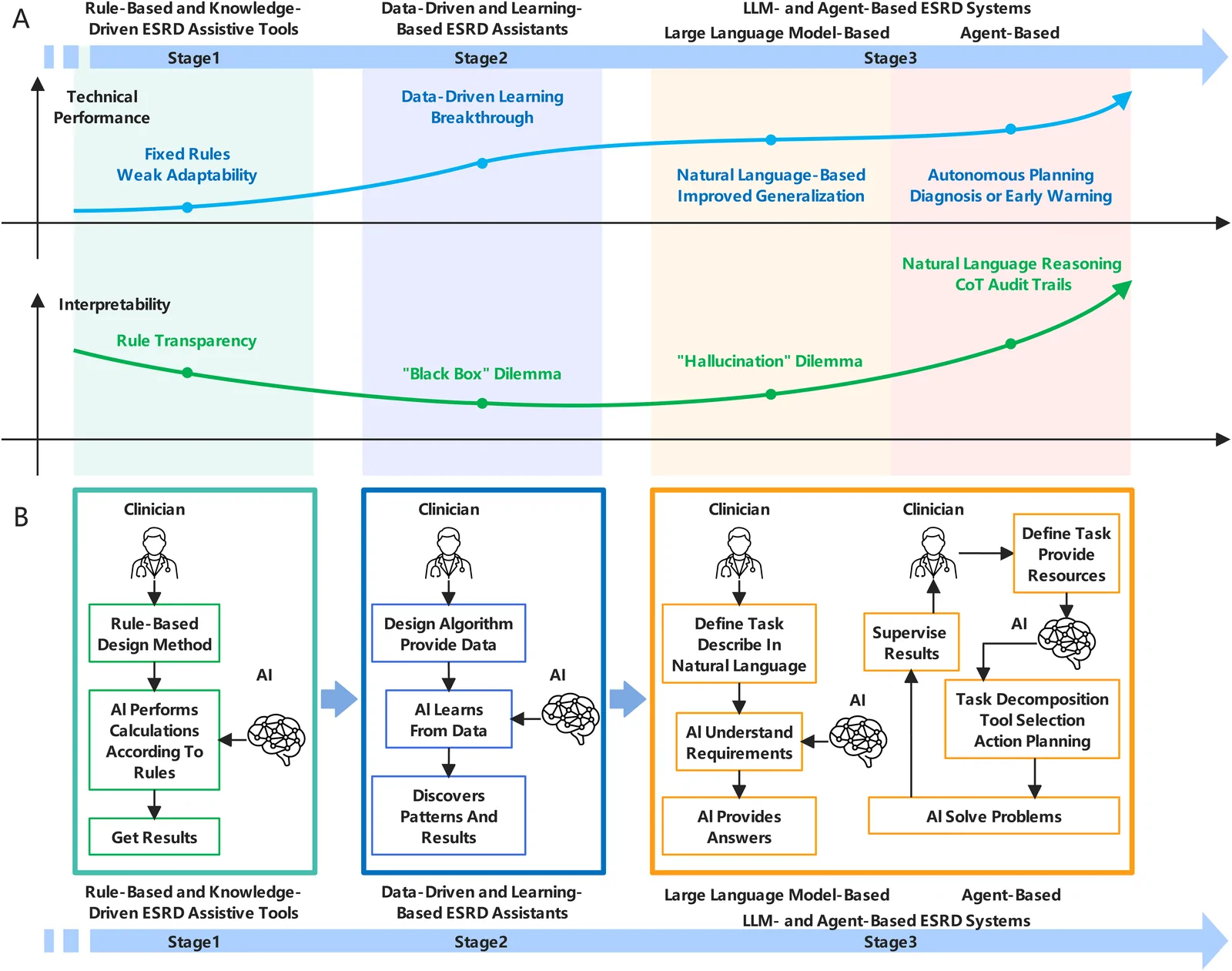
Evolution of AI development patterns in ESRD care (systematic review corpus; n = 100 studies). (A) Performance and interpretability trajectories: Stage 1 (n = 10) rule-based baselines (accuracy ~84%); Stage 2 (n = 83) AUC 0.80–0.94 with “black box” challenge addressed by SHAP/LIME; Stage 3 (n = 7) preliminary evidence for broad task versatility (task accuracy ~71–95% across the seven ESRD-specific studies; dotted projection denotes limited validation). (B) Human–machine relationship evolution from tool-assistive (Stage 1) to intelligent assistant (Stage 2) to emerging cognitive partnership (Stage 3). Dashed border denotes Stage 3 as an emerging research direction.

The application trajectory of artificial intelligence in ESRD patient management exemplifies the paradigmatic evolution from rule-based assistive tools to cognitive collaborative partners. Through comprehensive cross-stage analysis, four fundamental developmental patterns emerge that define the technological progression and clinical integration pathways in this field, as illustrated in Fig 12.

#### 5.4.1. Staged performance development: from stable baselines to adaptive performance.

The three-stage evolution demonstrates distinct performance development trajectories reflecting fundamental shifts in technological capabilities and clinical utility.

**Stage 1: Stable but Limited Performance Foundation.** Early rule-based systems established consistent performance baselines within specific domains, exemplified by Geddes et al.’s modified Charlson score achieving 84.2% accuracy in survival prediction [30]. Representative systems included Raghavan et al.’s treatment decision support frameworks [29] and Miskulin et al.’s anemia management tools [33]. While these systems demonstrated reliability in standardized scenarios, rule rigidity constrained adaptation to complex clinical variations and atypical patient presentations.

**Stage 2: Improved Performance through Data-Driven Learning.** Machine learning approaches achieved substantial performance enhancement, with AUC values consistently reaching 0.80-0.90 across ESRD applications. Notable achievements include Ali et al.’s XGBoost model for transplant survival prediction (C-index 0.74, AUC 0.76) [108], Zhang et al.’s random forest system for rapid peritonitis diagnosis (sensitivity 98.5%, AUC 0.993) [39], and Lee et al.’s RNN-based hypotension monitoring (AUC 0.94) [63]. This fundamental transformation from rule simulation to empirical pattern learning enabled improved modeling of complex nonlinear clinical relationships.

**Stage 3: Early Evidence for Versatility, with Important Caveats.** The seven ESRD-specific Stage 3 studies point to potential for broad adaptability: large language models supported patient education, nutritional guidance, pathology-report interpretation, and transplant decision support across hemodialysis, peritoneal dialysis, and kidney transplantation, with reported task accuracies generally in the 71–95% range [18–20,120–123]. In the wider biomedical AI literature, related approaches illustrate where this direction may lead, for instance, Gaber et al.’s retrieval-augmented workflow reached 65.75% exact-match accuracy in general clinical decision support [24], and Liu et al.’s MedGen multi-agent architecture provided interpretable decision support through multi-source knowledge fusion [149]—though neither was developed in an ESRD population. These results are indicative rather than conclusive, given the small number of ESRD-specific studies, the absence of any formal validation procedure in five of the seven, and the limited prospective, real-world evaluation (only one prospective pilot trial). Stage 3 performance should therefore be understood as promising early evidence for a research direction that has not yet reached the validation maturity of Stage 2.

#### 5.4.2. Interpretability evolution: the dynamic transparency-performance trade-off.

The interpretability trajectory across developmental stages reveals a complex evolutionary path balancing technological advancement with clinical transparency requirements.

**Inherent Transparency in Rule-Based Systems.** Early AI systems possessed intrinsic interpretability through explicit “if-then” logical structures. Clinicians could directly review and modify rule libraries, ensuring alignment with clinical guidelines while maintaining complete transparency in decision-making processes [28,36]. However, this transparency came at the cost of limited capability in handling complex atypical cases [32,33].

**Post-Hoc Explanation Era.** Learning-based methods introduced the “black box” challenge, necessitating post-hoc explanation frameworks. SHAP and LIME methodologies enabled retrospective decomposition of predictions into feature-level contributions [92,118], while attention mechanisms and visualization techniques addressed CNN-based system transparency. Despite notable progress, explanation capabilities remained constrained by the fundamental trade-off between model complexity and interpretability depth [90].

**Natural Language Interpretability Renaissance.** Large language models introduced intuitive explanation capabilities through conversational interfaces, yet maintained fundamental opacity in decision mechanisms. Suppadungsuk et al. documented significant “hallucination” issues in nephrology literature searches [150], highlighting the superficial nature of language-based interpretability. However, agent systems demonstrate promising solutions through structured reasoning processes, chain-of-thought methodologies, and explicit decision audit trails [151,152].

The evolutionary progression follows a clear trajectory from “high interpretability-low performance” through “low interpretability-high performance” to the current “moderate interpretability-excellent performance” paradigm explored by advanced agent architectures [144].

#### 5.4.3. Human-machine relationship transformation: from tools to cognitive partners.

The evolution of human-machine relationships in ESRD management reflects fundamental shifts in AI autonomy and clinical integration patterns.

**Tool-Assistive Relationships.** Stage 1 systems functioned as passive tools with complete human dominance. AI behavior was entirely driven by explicit rules, requiring continuous human supervision and manual rule adjustment [28,31]. The interaction paradigm was unidirectional and instructional, with AI systems lacking learning capabilities or autonomous adaptation mechanisms.

**Intelligent Assistant Collaboration.** Stage 2 marked transformation toward bidirectional collaborative relationships. AI systems gained autonomous pattern recognition capabilities, achieving or exceeding human expert performance in specific tasks [90,99]. However, “black box” characteristics necessitated human interpretation and validation of AI outputs, establishing a collaborative framework where AI discovered patterns while humans provided clinical context and decision validation.

**Cognitive Partnership Evolution.** Stage 3 represents progression toward sophisticated collaborative partnerships. Large language models enable deep semantic communication through natural language interfaces, allowing clinicians to specify task parameters and request detailed decision rationale [20,122]. Agent systems further elevate collaboration through autonomous task decomposition, multi-tool coordination, and comprehensive decision documentation [145]. This “human-in-the-loop” paradigm maintains clinical oversight while maximizing AI autonomous efficiency.

This technological evolution manifests through three fundamental paradigmatic shifts: supervisory control transitioning from complete human oversight to adaptive authority delegation, communicative modalities evolving from command-driven interfaces to dynamic conversational partnerships, and cognitive architectures advancing from computational adjuncts to autonomous decision-making entities capable of independent clinical reasoning.

#### 5.4.4. Technological complexity and adaptation trajectories.

Cross-stage analysis reveals progressive enhancement in task complexity handling, methodological sophistication, and autonomous adaptation capabilities. Early rule-based systems required extensive expert manual input with limited flexibility, while machine learning methods achieved automation improvements despite strong data dependencies. Current agent systems demonstrate advanced intelligent interaction with reduced requirements for manual annotation and feature engineering, suggesting future clinical implementations may achieve enhanced utility with decreased human intervention requirements while maintaining safety and efficacy standards.

#### 5.4.5. Data paradigm evolution and sample requirements.

**Fig 13:**
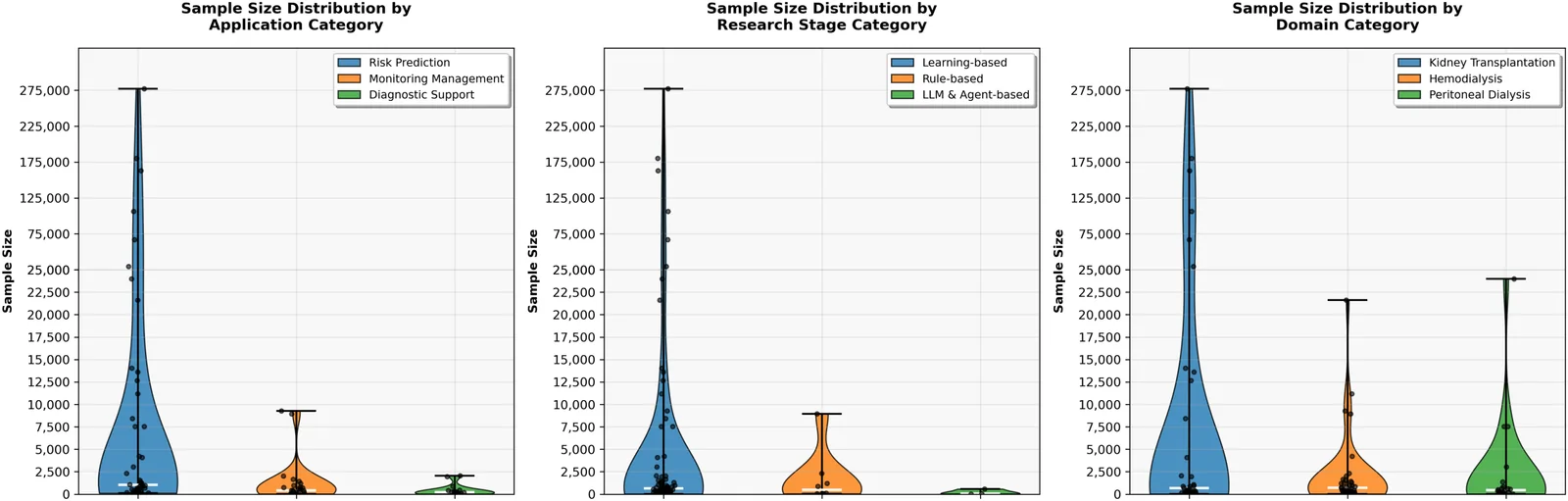
Sample size distribution by AI developmental stage (systematic review corpus; n = 100 studies). Box plots show range, IQR, and median for Stage 1 (rule-based; n = 10), Stage 2 (data-driven; n = 83), and Stage 3 (LLM/agent; n = 7). Log-scale x-axis.

The three-stage progression demonstrates fundamental shifts in data utilization paradigms and sample size dependencies. Rule-based systems operated with modest, precisely curated datasets (range: 42–8,941 patients, mean: 1,699 patients), prioritizing expert knowledge over empirical learning. Learning-based methods demanded substantially larger samples spanning five orders of magnitude (17–277,316 cases, mean: 12,508 cases), reflecting their dependency on training data volume for pattern recognition. Advanced cognitive systems paradoxically demonstrated reduced traditional sample requirements (mean: 209 cases), leveraging pre-trained knowledge from massive text corpora rather than task-specific datasets. The distribution characteristics of sample sizes are shown in Fig 13.

This evolution validates the progression from knowledge-engineering through data-intensive learning to knowledge-transfer paradigms, suggesting future ESRD AI systems may achieve enhanced clinical utility with reduced data collection burdens while maintaining high performance through advanced pre-training methodologies.

#### 5.4.6. Application mode selection and clinical integration patterns.

Cross-stage technological evolution reveals distinct patterns in application mode selection and clinical integration strategies. Rule-based systems emphasized standardized, protocol-driven applications with high reliability in routine scenarios but limited adaptability. Learning-based systems enabled sophisticated pattern recognition and predictive modeling, achieving breakthrough performance in complex clinical scenarios while requiring extensive validation protocols. Advanced cognitive systems demonstrate notable versatility, transitioning from specialized single-task optimization toward comprehensive multi-task platforms capable of autonomous reasoning and proactive clinical management.

The progression from reactive response to proactive prevention represents a fundamental paradigm shift. Early systems responded to explicit clinical queries, learning-based systems enabled predictive risk stratification, while agent systems approach autonomous health monitoring and intervention recommendation. This evolution suggests that future ESRD management will transition from physician-initiated AI consultation toward AI-driven continuous patient surveillance and proactive clinical support.

#### 5.4.7. Analysis of technological evolution pathways and application distribution characteristics.

**Fig 14:**
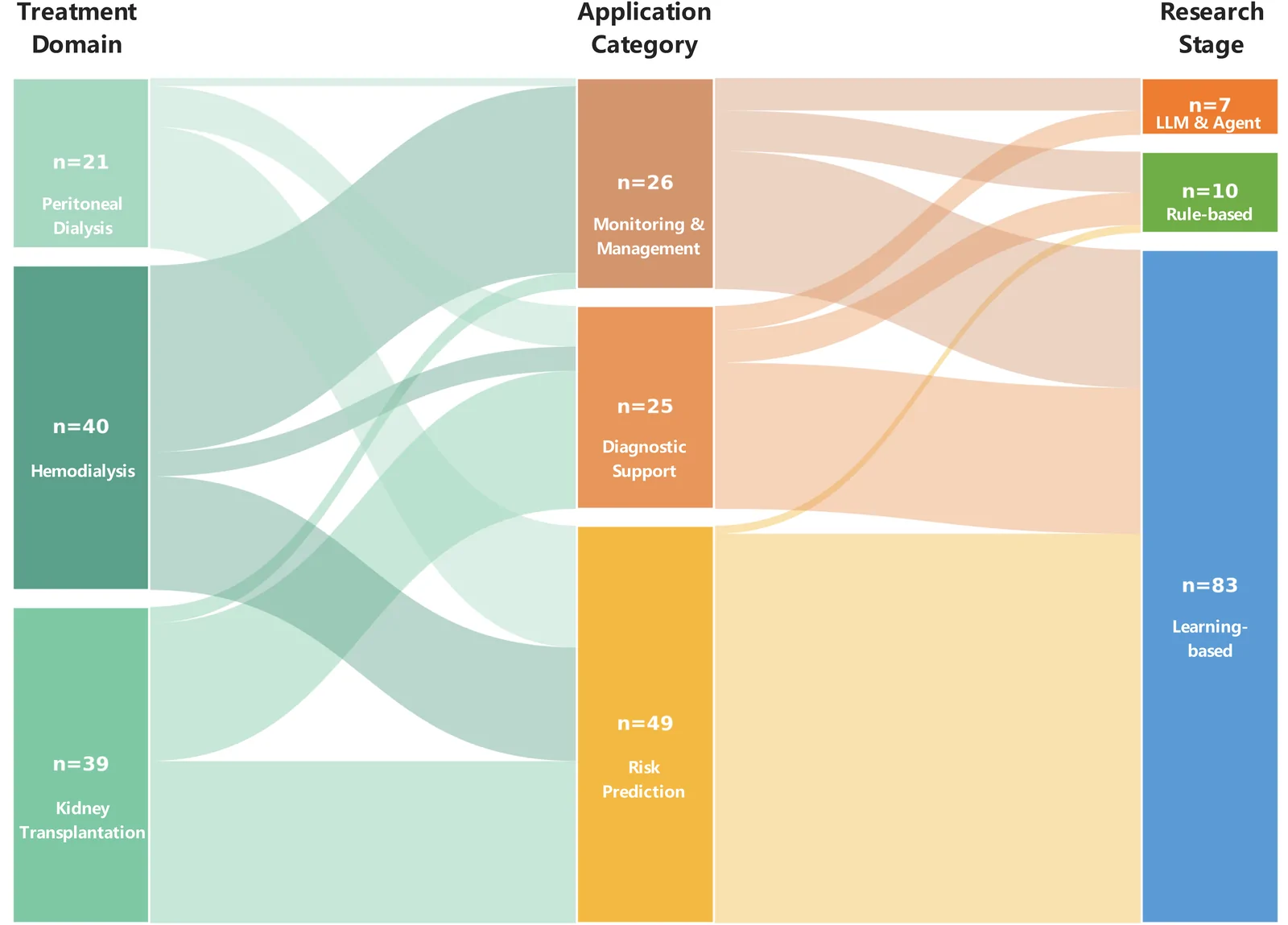
AI method, application domain, and treatment modality distributions (systematic review corpus; n = 100 studies). Sankey diagram; flow widths proportional to study count within each pathway.

The following synthesis characterizes the technological landscape and application distribution observed across all 100 included ESRD studies. The Sankey diagram illustrating the three-dimensional relationships among technical methods, application domains, and treatment modalities is shown in Fig 14.

Technological stratification across the 100 ESRD-focused studies reveals a clear developmental gradient. Learning-based methods (Stage 2) predominate (83.0%; 83/100), reflecting the maturity of supervised machine learning in clinical ESRD research. Rule-based systems (Stage 1) account for 10.0% (10/100), retaining clinical relevance in scenarios demanding transparent, protocol-driven decision support. Large language model- and agent-based approaches (Stage 3) represent 7.0% (7/100) of the corpus—a proportion that, while modest, accurately reflects the nascent but rapidly expanding state of cognitive AI research in ESRD at the time of this review.

Application domains cluster into three primary categories: risk prediction (49.0%; 49/100), monitoring and management (26.0%; 26/100), and diagnostic support (25.0%; 25/100). Treatment modalities exhibit specialized distribution patterns: hemodialysis studies emphasize real-time monitoring and management (57.5%; 23/40); kidney transplantation research prioritizes prognostic assessment (51.3% risk prediction [20/39], 43.6% diagnostic support [17/39]); and peritoneal dialysis investigations focus predominantly on risk stratification (71.4%; 15/21), commensurate with the self-management demands of home-based care.

Technology–application convergence reveals domain-specific optimization. Risk prediction demonstrates near-universal adoption of learning-based methods (98.0%; 48/49), reflecting their greater capacity for pattern recognition in complex prognostic modeling. Diagnostic support exhibits greater methodological diversity, corresponding to the heterogeneity of imaging and biomarker tasks involved. Monitoring applications are increasingly integrating multi-modal data streams, with early-stage LLM applications beginning to augment patient education and clinical documentation workflows.

Taken together, these patterns chart a trajectory from data-driven precision medicine toward comprehensive cognitive assistance, pointing to a future in which ESRD management will be characterized by tightly integrated, personalized human–AI collaborative systems.

## 6. Challenges facing AI in ESRD patient diagnosis, treatment, and management

With the continuous advancement of artificial intelligence technology, its application in end-stage renal disease (ESRD) diagnosis, treatment, and management exhibits distinct generational evolution characteristics, with core challenges at each stage undergoing profound transformations. From early rule-based expert systems to machine learning and deep learning approaches, and subsequently to large language models and intelligent agent systems, the AI challenges in the ESRD domain have experienced a fundamental shift from “knowledge engineering” to “data quality” and ultimately to “reliable execution”. The dominant challenges across different AI development stages are shown in Table 6.

**Table 6:** Dominant challenges and representative evidence across different AI development stages.

Stage	Dominant Challenges	Representative Evidence
Rule-based	Incomplete knowledge coverage; limited thresholds/specificity; workflow integration and alert fatigue	[35],[33,36,37]
Learning-based	Data quality/bias/single-center; external generalizability and calibration; overfitting; unproven clinical utility	[94],[88,99,108,118,119]
LLM-based	Knowledge temporality and hallucination; ethics/privacy/compliance; integration standards and fairness; deployment barriers	[124],[19–21,122,153,154]
Agent-based	Tool/action reliability and auditability; real-time efficiency; alignment/safety boundaries; unstructured data challenges; clinical-grade validation gaps	[23],[144,151,152,155,156]

Abbreviations: AI, artificial intelligence; LLM, large language model. Bracketed numbers in the “Representative Evidence” column are reference citations.

### 6.1. Challenges in rule-based systems: the dilemma of knowledge coverage and process integration

Early rule-based expert systems in ESRD management face the primary challenge of incomplete knowledge coverage and rapid escalation of system complexity. Due to the diversity and complexity of renal disease etiologies, rule-based systems struggle to encompass all possible clinical scenarios, resulting in a coexisting dilemma of omissions and false positives [36]. The limitations of threshold setting further exacerbate this problem, particularly in critical applications such as vascular access risk identification, where systems maintain persistently high false-positive rates while failing to achieve early warning capabilities [35].

More critically, workflow integration challenges emerge as a significant concern. Many rule-based systems generate unexpected adverse outcomes due to insufficient embedding within authentic clinical processes, such as the absence of critical components in iron management protocols, which may introduce clinical risks [33]. The widespread prevalence of alert fatigue further undermines the clinical impact of these systems, as healthcare providers develop desensitization to frequent false alerts, paradoxically reducing sensitivity to genuine risks [37]. In the context of complex dialysis operations and constrained human resources, rule-based systems struggle to dynamically absorb frontline clinical experience and adapt to process changes, limiting their practical value [29].

### 6.2. Machine learning era challenges: the gap between data quality and clinical translation

Entering the machine learning and deep learning era, the focus of challenges shifts markedly toward data quality and external generalizability of models. The limitations of retrospective studies, single-center data bias, small sample sizes, and variable heterogeneity and imbalance directly constrain model generalization capabilities [99,118]. Differences in data collection standards across countries and healthcare systems present enormous challenges for building globally applicable predictive models, with existing models requiring significant improvement in discrimination and calibration [108,119].

Overfitting problems are particularly pronounced in dynamic prediction tasks, where complex models such as artificial neural networks tend to become overly dependent on training data, while quantification of prediction uncertainty remains challenging [88]. More critically, most models lack adequate real-world clinical validation, with their clinical utility yet to be conclusively demonstrated. Insufficient consideration of emerging risk factors such as donor genetic characteristics and patient adherence, along with limited predictive capability for intervention effects, further restricts the clinical application value of these models [94]. Resource investment and implementation cost considerations, including the complexity of variable selection, temporal model drift, and ongoing maintenance requirements, also constitute significant constraints on model deployment [99].

### 6.3. Large language model challenges: new requirements for knowledge temporality and ethical compliance

The application of large language models in ESRD management introduces new dimensions of challenges. Knowledge temporality and factual accuracy emerge as primary concerns, with outdated information, generalized responses, or hallucinated outputs generated by models posing direct threats to clinical decision-making reliability [19,122,153]. Privacy protection and data security issues involving medical documentation and patient interactions demand stricter compliance controls, with blurred responsibility boundaries further exacerbating these challenges [21,124].

Fairness issues exposed during clinical integration cannot be overlooked. Differences in AI information quality perception among populations with varying racial and educational backgrounds highlight potential risks of digital divides and health equity [20,122]. Deployment modality choices create conflicts between the convenience of cloud-based APIs and institutional privacy protection requirements, with high technical barriers for localized deployment and difficulties in knowledge updates and clinical guideline integration further complicating the implementation process [154].

### 6.4. Intelligent agent system challenges: from “speaking” to “acting” with reliability assurance

Intelligent agent systems represent the new frontier of AI applications in ESRD management but also introduce unprecedented challenges. Tool invocation and action execution reliability become core concerns, with multi-tool coordination and complex execution chain fault tolerance requiring the establishment of comprehensive self-correction mechanisms and reward constraint systems to prevent medical hallucinations and misuse risks [23]. In time-sensitive scenarios such as emergency departments, systems may exhibit severe overestimation and resource misallocation, emphasizing the importance of real-time control and precise calibration [155].

Maintaining alignment and safety boundaries becomes increasingly complex during multi-step reasoning and execution processes. Risks of prompt injection attacks and privilege escalation require implementation of strict protective measures, including task decomposition, privilege control, and comprehensive audit mechanisms [156]. Information extraction and matching of clinical trial inclusion and exclusion criteria from unstructured electronic health records continue to face technical challenges, while requiring balance between privacy protection and system operability [151].

Current intelligent agent system evaluations are largely limited to benchmark testing and synthetic scenarios, with significant gaps remaining from real-world patient-physician interactions and prospective multi-center trials [23]. Limitations of generalist medical models and multi-agent systems in knowledge updating, individualized treatment, and explainability require thorough validation and correction in real-world applications [144,152].

## 7. Evolution of future prospects for AI in ESRD patient diagnosis and treatment management

The future development of artificial intelligence in end-stage renal disease diagnosis and treatment management demonstrates a clear evolutionary trajectory, progressing from the early basic goal of “connecting systems to clinical practice” to the advanced objective of “enabling systems to act safely.” This evolutionary process reflects the fundamental transformation of AI technology from assistive tools to intelligent decision-making partners, with future prospects at each developmental stage embodying different technological priorities and application value orientations. Future prospects and development directions of different AI development stages are shown in Table 7.

**Table 7:** Future prospects and development directions of different AI development stages.

Stage	Main Development Directions	Representative Evidence
Rule-based	Integrated automation and machine learning fusion; expanded variable coverage; individualized treatment model integration; process-oriented continuous monitoring; interdisciplinary expert network establishment	[35],[28,32,33,36,37]
Learning-based	Multicenter validation and real-world applications; large-scale consortium data construction; external validation and policy implementation; integrated learning model deployment; HLA characterization and clinical data fusion	[105],[88,90,93,99,108,114,118,119,150–156]
LLM-based	Specialized deployment and multimodal integration; factuality enhancement and engineering deployment; prompt optimization and retrieval-augmented generation; ethical legal framework establishment; patient experience and equity concerns	[18–20,120,122,124,153,154]
Agent-based	Closed-loop execution and auditable safety; multi-tool multimodal agent construction; real-time performance and efficiency optimization; alignment safety and regulatory frameworks; real-world validation and continuous learning	[22],[144,151,155–158]

Abbreviations: AI, artificial intelligence; LLM, large language model; HLA, human leukocyte antigen. Bracketed numbers in the “Representative Evidence” column are reference citations

### 7.1. Prospects for rule-based systems: integrated automation and machine learning fusion

Future development of rule-based expert systems primarily focuses on enhancing system integration capabilities and fusion with emerging technologies. Expanding input variable coverage and combining with machine learning and deep learning methods has become a key pathway for enhancing system performance and usability [36]. In critical applications such as vascular access management, future development will evolve toward process-oriented and continuous monitoring, achieving deep integration of systematization and automation through incorporation of continuous access monitoring mechanisms with weekly population reviews [35].

Integration of individualized treatment models represents an important direction for rule-based system development. In anemia management, introducing more complex individualized models and iron management mechanisms can improve treatment outcomes and patient prognosis [33]. Sustainable transformation from evidence-based medicine to clinical practice requires in-depth research on system usability, alert appropriateness, and clinical compliance to ensure long-term application sustainability [37]. Establishment of interdisciplinary expert networks and implementation of standardized risk management strategies will drive deep integration of rule-based systems in routine clinical practice [28,32].

### 7.2. Machine learning era prospects: multicenter validation and real-world applications

Future development of machine learning methods focuses on constructing more robust and generalizable predictive models. Large-scale consortium data construction has become a key foundation, enabling training of more robust post-transplant event prediction models through regional or national registry databases and cross-center collaborative networks such as NephroCAGE [118]. External validation and policy-level implementation will promote greater roles for AI tools in optimizing organ matching and improving transplant success rates, facilitating broader system adoption and practice updates [108,119].

Model implementability and scalability have improved with advances in computational power and tools. Deployment of integrated learning models in practice has become more convenient, allowing fine-tuned submodel customization based on patient characteristics and task requirements [90]. While maintaining fair allocation principles, constructing practical prognostic tools using routine clinical variables to maximize donor-recipient matching benefits reflects the balance between technological advancement and ethical considerations in AI applications [93]. Feature dimension expansion, particularly deep fusion of HLA characterization with clinical data, and construction of biomarker discovery pathways toward clinical trials and drug development will lay foundations for precision medicine applications in ESRD management [105,114].

### 7.3. Large language model prospect: specialized deployment and multi- modal integration

Future development of large language models focuses on factuality enhancement and optimization of engineering deployment pathways. Through application of prompt optimization, specialized fine-tuning, and retrieval-augmented generation (RAG) technologies, medical factual accuracy can be improved while developing toward privatized deployment and no-code deployment directions [120,153,154]. Multimodal capability integration will incorporate document parsing, laboratory reports, medical imaging, and wearable device data into unified workflows, forming composite scenario applications for nutritional management and follow-up monitoring [18,124].

Establishment of ethical legal frameworks and practice standards is crucial for LLM applications in high-risk medical procedures. In critical decision-making scenarios such as kidney transplantation, establishing prudent governance mechanisms and transparent, safe application boundaries is necessary [19,122]. Focus on equity and patient experience reflects human-centered development concepts, promoting patient-centered interaction design and improvement of explanation mechanisms through attention to differences in AI information quality perception among different populations [20].

### 7.4. Agent system prospects: closed-loop execution and auditable safety

Agent systems represent the most cutting-edge development direction for AI applications in ESRD management, with the core objective of achieving a fundamental leap from “can answer” to “can act safely”. Construction of multi-tool multimodal medical agents will enhance planning-execution-self-correction capabilities and interpretability, developing toward safe and usable decision support systems [157,158]. In information retrieval and matching tasks emphasizing both structured and unstructured data, particular emphasis is needed on balancing privacy protection, cost-effectiveness, and process integration [151].

Real-time performance and efficiency optimization are particularly important in time-sensitive clinical scenarios. In critical settings such as emergency care, careful evaluation and iterative improvement are needed to reduce system complexity and response latency to effectively complement clinical decision-making [155]. Establishment of alignment safety and regulatory frameworks is crucial for systems oriented toward “can act,” requiring clear ethical regulatory pathways and design of auditable and traceable guardrail mechanisms and human-machine collaboration models [156]. Transition from benchmark testing to real-world validation will drive evaluation and continuous learning of closed-loop processes such as automated reimbursement decisions and medical necessity justification in real scenarios [22,144].

## 8. Discussion

### 8.1. Performance versus practice: the gap between benchmark and bedside

The consistent achievement of AUC values in the 0.80–0.90 range across Stage 2 studies might, on first reading, suggest that the field is approaching clinical readiness. Yet the findings of this review reveal a more nuanced picture. Of the 83 Stage 2 studies analyzed, 97.6% reported at least one internal validation protocol, while only 26.5% included external validation on an independent dataset—and none reported prospective clinical validation as part of the primary evaluation design. This pattern captures a fundamental tension in medical AI research: statistical performance on held-out test splits does not translate automatically into reliable behavior when a model encounters the heterogeneity of real-world clinical populations.

Several mechanisms underlie this gap. Single-center retrospective datasets, which constitute the majority of the reviewed literature, introduce systematic biases in patient selection, data collection protocols, and outcome definitions that are rarely reproduced across institutions. When a model is deployed in a new center with different electronic health record systems, dialysis machine configurations, or patient demographics, performance frequently degrades substantially. This phenomenon, commonly referred to as data drift, is particularly pronounced in ESRD because treatment protocols, fluid management thresholds, and laboratory reference ranges vary considerably across facilities and healthcare systems. The relatively small sample sizes characteristic of many ESRD studies (Stage 2 median 641 cases) further compound the risk of overfitting, whereby models capture dataset-specific noise rather than generalizable clinical signal. Until multicenter prospective validation becomes a routine expectation rather than an exception, the gap between benchmark performance and bedside utility will persist.

### 8.2. Structural barriers to clinical translation

Even where model performance is robustly validated, translation into routine clinical deployment involves at least three distinct categories of structural barriers.

**Regulatory and compliance barriers.** Medical AI systems in most jurisdictions must obtain formal regulatory clearance before clinical use—a pathway that remains nascent and evolving. Regulatory bodies including the U.S. Food and Drug Administration (FDA), the European Medicines Agency, and China’s National Medical Products Administration (NMPA) are still developing frameworks capable of addressing AI-specific challenges such as continuous learning, post-market performance monitoring, and accountability when AI-assisted decisions lead to adverse outcomes. The opacity of deep learning models creates particular difficulties: regulators accustomed to reviewing fixed-parameter devices face genuine difficulties when the model to be evaluated can, in principle, change its behavior after deployment. These structural uncertainties create a compliance burden that most research groups, as well as many smaller clinical institutions, are not equipped to navigate, effectively limiting deployment to large academic centers with dedicated regulatory and legal infrastructure.

**Technical and integration barriers.** Even technically sound models face substantial implementation challenges in practice. The lack of standardized data schemas across electronic health record systems means that features used to train a model in one environment may not exist, may be named differently, or may be coded using different terminologies in another. Real-time inference in time-sensitive scenarios, such as intradialytic hypotension prediction during an active hemodialysis session, requires low-latency computation that many hospital IT environments cannot currently support without dedicated hardware investment. More fundamentally, the vast majority of reviewed models were developed and validated as standalone algorithmic entities, with no formal integration into clinical workflows, user interfaces, or decision-support architectures. Bridging this gap requires expertise in health informatics, human factors engineering, and clinical implementation science that lies outside the skill set of most AI research teams.

**Clinical acceptance and equity barriers.** Clinician adoption of AI-based decision support depends not only on demonstrated performance but also on transparency, trust, and demonstrated safety. The “black box” characteristics of complex machine learning models remain a significant barrier to physician acceptance, even in cases where post-hoc explanation methods such as SHAP or LIME have been applied. Explanation methods of this type describe the statistical contribution of variables to model outputs rather than causal mechanisms, and experienced clinicians, particularly those with training grounded in mechanistic reasoning, often find these explanations unsatisfying as a basis for high-stakes clinical decisions. Alert fatigue, documented in the context of rule-based systems in Stage 1 and now re-emerging in different forms with Stage 2 monitoring applications [37], further erodes clinical receptiveness. Equity concerns also warrant explicit attention: differences in AI prediction quality across racial and educational groups have already been documented in the ESRD context [20], reflecting training data that systematically underrepresent patients from low- and middle-income countries and minority populations. Models that perform well on average may perform poorly for the patients with the greatest clinical need, potentially introducing harmful allocation biases.

### 8.3. Pathways toward responsible clinical translation

Addressing these barriers requires coordinated efforts that extend beyond algorithm development. Multicenter prospective validation trials should be treated as a necessary step before any model is considered for routine clinical deployment, rather than as aspirational future work. Regulatory engagement should begin early in the development process, with study designs crafted to meet the evidentiary standards required for formal clearance. Human-in-the-loop system architectures, which preserve clinical oversight while reducing unnecessary intervention burden, offer a pragmatic design principle for near-term implementation. Finally, meaningful representation of patient populations from diverse geographic, racial, and socioeconomic backgrounds in training and validation datasets is not merely a methodological refinement but a prerequisite for equitable AI deployment in a field where health disparities are already substantial.

## 9. Limitations

This review has several limitations that should be considered when interpreting its findings.

### 9.1. Language bias

The search was restricted to English-language peer-reviewed literature. Among the 100 included studies, China affiliated the largest number of studies (43 studies; 23.6% of all paper-country pairs), and a substantial volume of clinically relevant AI research in nephrology is published in Chinese-language journals. This criterion therefore likely results in systematic underrepresentation of studies from Chinese institutions. The reported geographic and application-domain distributions should be interpreted with this directional bias in mind.

### 9.2. Grey literature and trial registries not searched

Clinical trial registries (e.g., ClinicalTrials.gov) and grey literature sources were not included in the search. This decision reflects the focus of the review on peer-reviewed studies reporting quantifiable AI performance outcomes; however, it means that negative findings, implementation studies, and early-phase clinical evaluations, most commonly available only as preprints or registry records, are almost certainly underrepresented. Whether this introduces systematic bias in the direction of reported performance estimates cannot be determined from the available evidence.

### 9.3. Limited number of Stage 3 ESRD-specific studies

Only 7 of the 100 included studies employed large language model- or agent-based approaches in ESRD-specific populations. While this proportion accurately reflects the current state of the research frontier, it limits the statistical reliability of Stage 3 comparative analyses. Findings relating to LLM and agent applications in ESRD should be interpreted as preliminary and directional rather than definitive.

### 9.4. Absence of meta-analysis

The substantial heterogeneity across included studies, spanning AI methodology, outcome definitions, patient populations, and performance metrics, precluded formal meta-analysis. Performance statistics reported across studies (e.g., AUC ranges and means) represent narrative summaries rather than pooled estimates, and cross-study comparisons should be interpreted accordingly. Development of standardized reporting frameworks for AI studies in nephrology would facilitate more rigorous quantitative synthesis in future reviews.

### 9.5. Cross-sectional scope

The literature search was conducted up to June 2025, and findings represent a fixed temporal cross-section of a rapidly evolving field. The review captures neither the real-world longitudinal performance of deployed models nor the trajectory of any individual model’s clinical impact over time. Studies published after the search cutoff, including any trials currently underway, are not reflected in this synthesis.

### 9.6 sAbsence of prospective protocol registration

The review protocol was not registered in a public registry (e.g., PROSPERO). Although the eligibility criteria, search strategy, and analytical framework were defined a priori, the absence of a time-stamped public protocol limits independent verification of the prespecified plan.

## 10. Conclusion

This systematic review of 100 ESRD-focused studies characterizes the technological evolution of AI in ESRD care through a three-stage developmental framework, and synthesizes evidence on application domains, model performance, validation maturity, and persistent barriers to clinical translation.

AI applications across the 100 included studies cluster into three primary domains: risk prediction (49.0%), monitoring and management (26.0%), and diagnostic support (25.0%); each exhibits distinct clinical priorities across hemodialysis, peritoneal dialysis, and kidney transplantation. The three-stage framework reveals a coherent developmental arc. Stage 1 rule-based systems (1980s–2010s; n = 10 ESRD studies) established high interpretability through explicit decision logic but were constrained in performance and scalability. Stage 2 data-driven learning systems (2010s–2020s; n = 83) achieved substantial performance gains, with AUC values in the 0.80–0.90 range across application domains, through autonomous pattern recognition, at the cost of interpretability. Stage 3 large language model- and agent-based systems (2020s–present; n = 7 ESRD studies) represent an emerging research direction characterized by broad task versatility and natural language reasoning, though formal validation in ESRD-specific populations remains limited and findings should be interpreted as preliminary.

Across the full corpus, a persistent tension is evident between statistical performance and clinical readiness. Stage 2 models consistently demonstrate clinically relevant AUC values, yet while 97.6% of Stage 2 studies employed at least one formal validation protocol, only 26.5% included external validation, and none reported prospective clinical evaluation as part of the primary design. This gap, well documented in the broader medical AI literature, reflects structural barriers including single-center retrospective data, limited external generalizability, and the absence of regulatory frameworks tailored to continuously learning AI systems. The developmental challenges have shifted across stages: from incomplete knowledge coverage in Stage 1, to data quality and overfitting in Stage 2, to hallucination risk, ethical compliance, and deployment reliability in Stage 3.

Looking ahead, progress in this field will require coordinated effort along several axes: multicenter prospective validation as a standard expectation rather than an aspirational goal; early regulatory engagement in AI system development; and inclusive training datasets that adequately represent the diverse patient populations, spanning geographic, racial, and socioeconomic dimensions, for whom ESRD care is most consequential. Stage 3 systems, while not yet validated at the scale of their Stage 2 counterparts, point toward a future in which AI functions less as a narrow task-specific tool and more as a cognitively flexible assistant capable of supporting complex, longitudinal clinical reasoning. Realizing that potential will depend on advances in model reliability, safety alignment, and human-AI collaboration frameworks designed for high-stakes healthcare environments.

## Supporting information

S1 Text Complete database-specific search strategies. Verbatim search strings for PubMed, IEEE Xplore and Web of Science, with search dates, platforms, field tags and record yields for each database.
(DOCX)

S2 Text Extended bibliometric analysis. Full geographic distribution, international co-authorship network, institutional output and author profile analyses underlying Sections 3.2 and 3.3 and Figs 3–6.
(DOCX)

S1 Table Risk-of-bias and reporting-quality assessment of the 100 included studies. Study-level PROBAST, QUADAS-2 and DECIDE-AI domain ratings, with domain-level inter-rater agreement statistics.
(DOCX)

S1 Data Studies excluded at the full-text eligibility stage. Numbered list of all 952 records excluded after full-text assessment, with title, authors and the primary reason for exclusion for each record.
(XLSX)

S1 File PRISMA 2020 checklist. Completed checklist identifying where each PRISMA 2020 item is reported in the manuscript and its supporting information.
(DOCX)

## Abbreviations

AUC: area under the receiver operating characteristic curve (some primary studies originally reported this quantity as AUROC or ROC; standardized to AUC throughout)
AUPRC: area under the precision–recall curve
ACC: accuracy
SENS: sensitivity
SPEC: specificity
PRE/PRC: precision
RECALL: recall
F1: F1 score
C-index: concordance index
IBS/IBC: integrated Brier score
IoU: intersection over union
Dice: Dice similarity coefficient
RMSE: root mean square error
MSE: mean squared error
MAE: mean absolute error
R^2^: coefficient of determination
ROC: receiver operating characteristic (curve)
CNN: convolutional neural network
RNN: recurrent neural network
LSTM: long short-term memory
MLP: multilayer perceptron
KNN: k-nearest neighbors
XGBoost: extreme gradient boosting
SVM: support vector machine
PCA: principal component analysis
KELM: kernel extreme learning machine (BSWEGWO-KELM and BSCWJAYA-KELM denote metaheuristic-optimized variants of KELM)
GPT: generative pre-trained transformer
BERT: bidirectional encoder representations from transformers
