# Supplementary material for: From assistant to collaborator: A systematic review of the evolution of artificial intelligence in end-stage renal disease care and management
Source: PLOS Digit Health. 2026 Sep 2;5(9):e0001635. doi: 10.1371/journal.pdig.0001635 (PMC13537592; doi:10.1371/journal.pdig.0001635)
Supplement: Supplementary file 1 [file pdig.0001635.s001.docx]

# S1 Text. Complete Database-Specific Search Strategies

*This supplementary file provides the complete, verbatim search strings used to retrieve records from each of the three databases searched in this systematic review, as required by PRISMA 2020 (Item 7; Page et al., 2021). The search was conducted by M.W. and independently verified by C.H. between January and June 2025.*

## S1.1 Overview

Three major academic databases were searched: **PubMed**, **IEEE Xplore**, and **Web of Science**. The search timeframe spanned **January 2005 to June 2025**, encompassing the full principal phases of AI development in healthcare, from rule-based clinical decision support systems through large language model applications. All searches were conducted in English. No language filter was applied at the search stage; however, subsequent full-text screening was limited to English-language publications.

The search strategy combined controlled vocabulary (MeSH headings in PubMed) with free-text terms in title, abstract, or all-metadata fields, as appropriate for each database. Core search concepts encompassed:

- **AI technology terms**: artificial intelligence, machine learning, deep learning, neural network(s), expert system(s), large language model(s), AI agent(s), cognitive agent(s)
- **ESRD-related clinical terms**: end-stage renal disease (ESRD), end-stage kidney disease (ESKD), hemodialysis, haemodialysis, peritoneal dialysis, kidney/renal transplantation, renal replacement therapy, kidney replacement therapy

## S1.2 PubMed Search String

**Search date:** June 2025 **Platform:** NCBI PubMed (https://pubmed.ncbi.nlm.nih.gov/) **Search field:** Combined MeSH terms and Title/Abstract [tiab] free-text

("Artificial Intelligence"[Mesh]
OR "Machine Learning"[Mesh]
OR "Deep Learning"[Mesh]
OR "Neural Networks, Computer"[Mesh]
OR "Expert Systems"[Mesh]
OR "Natural Language Processing"[Mesh]
OR "artificial intelligence"[tiab]
OR "machine learning"[tiab]
OR "deep learning"[tiab]
OR "neural network"[tiab]
OR "neural networks"[tiab]
OR "expert system"[tiab]
OR "expert systems"[tiab]
OR "large language model"[tiab]
OR "large language models"[tiab]
OR "LLM"[tiab]
OR "LLMs"[tiab]
OR "AI agent"[tiab]
OR "AI agents"[tiab]
OR "cognitive agent"[tiab]
OR "cognitive agents"[tiab])
AND
("Renal Dialysis"[Mesh]
OR "Peritoneal Dialysis"[Mesh]
OR "Kidney Transplantation"[Mesh]
OR "Renal Replacement Therapy"[Mesh]
OR "end-stage renal disease"[tiab]
OR "end stage renal disease"[tiab]
OR "ESRD"[tiab]
OR "end-stage kidney disease"[tiab]
OR "end stage kidney disease"[tiab]
OR "ESKD"[tiab]
OR "hemodialysis"[tiab]
OR "haemodialysis"[tiab]
OR "peritoneal dialysis"[tiab]
OR "kidney transplantation"[tiab]
OR "renal transplantation"[tiab]
OR "renal replacement therapy"[tiab]
OR "kidney replacement therapy"[tiab])
AND
(2005/01/01:2025/06/30[dp])
AND
(english[la])

**Initial records retrieved:** 1,323

## S1.3 IEEE Xplore Search String

**Search date:** June 2025 **Platform:** IEEE Xplore Digital Library (https://ieeexplore.ieee.org/) **Search field:** All Metadata

(
 ("All Metadata":"artificial intelligence")
 OR ("All Metadata":"machine learning")
 OR ("All Metadata":"deep learning")
 OR ("All Metadata":"neural network")
 OR ("All Metadata":"neural networks")
 OR ("All Metadata":"expert system")
 OR ("All Metadata":"expert systems")
 OR ("All Metadata":"natural language processing")
 OR ("All Metadata":"large language model")
 OR ("All Metadata":"large language models")
 OR ("All Metadata":"LLM")
 OR ("All Metadata":"LLMs")
 OR ("All Metadata":"AI agent")
 OR ("All Metadata":"AI agents")
 OR ("All Metadata":"cognitive agent")
 OR ("All Metadata":"cognitive agents")
)
AND
(
 ("All Metadata":"renal dialysis")
 OR ("All Metadata":"peritoneal dialysis")
 OR ("All Metadata":"kidney transplantation")
 OR ("All Metadata":"renal transplantation")
 OR ("All Metadata":"renal replacement therapy")
 OR ("All Metadata":"kidney replacement therapy")
 OR ("All Metadata":"end-stage renal disease")
 OR ("All Metadata":"end stage renal disease")
 OR ("All Metadata":"ESRD")
 OR ("All Metadata":"end-stage kidney disease")
 OR ("All Metadata":"end stage kidney disease")
 OR ("All Metadata":"ESKD")
 OR ("All Metadata":"hemodialysis")
 OR ("All Metadata":"haemodialysis")
)

**Date filter applied post-search:** 2005–2025 **Initial records retrieved:** 469

## S1.4 Web of Science Search String

**Search date:** June 2025 **Platform:** Web of Science Core Collection (https://www.webofscience.com/) **Search field:** Topic (TS=), which searches title, abstract, author keywords, and Keywords Plus®

TS=(
 (
 "artificial intelligence"
 OR "machine learning"
 OR "deep learning"
 OR "neural network"
 OR "neural networks"
 OR "expert system"
 OR "expert systems"
 OR "natural language processing"
 OR "large language model"
 OR "large language models"
 OR LLM
 OR LLMs
 OR "AI agent"
 OR "AI agents"
 OR "cognitive agent"
 OR "cognitive agents"
 )
 AND
 (
 "renal dialysis"
 OR "peritoneal dialysis"
 OR "kidney transplantation"
 OR "renal transplantation"
 OR "renal replacement therapy"
 OR "kidney replacement therapy"
 OR "end-stage renal disease"
 OR "end stage renal disease"
 OR ESRD
 OR "end-stage kidney disease"
 OR "end stage kidney disease"
 OR ESKD
 OR hemodialysis
 OR haemodialysis
 )
)
AND DOP=(2005-01-01/2025-06-30)

**Initial records retrieved:** 1,438

## S1.5 Search Results Summary

| Database | Initial Records Retrieved | After Title/Abstract Screening |
| --- | --- | --- |
| PubMed | 1,323 | 445 |
| IEEE Xplore | 469 | 51 |
| Web of Science | 1,438 | 1,041 (after de-duplication within database) |
| **Total** | **3,230** | — |
| After de-duplication (all databases combined) | 2,298 | — |
| After title/abstract screening | — | **1,052 assessed for full-text eligibility** |

*Note: Records were de-duplicated using Zotero reference management software, based on DOI, title, and author matching. The 1,052 records assessed for full-text eligibility constitute the bibliometric corpus used for publication trend and geographic analyses in Section 3 of the main text (see Section 2.6). Of these, 100 met all eligibility criteria and form the systematic review corpus on which Sections 4–7 are based.*

## S1.6 Terms and Rationale

**AI technology terms** were selected to capture the full spectrum of AI methodologies represented across the three developmental stages defined in this review:

- Stage 1 (rule-based): “expert system(s)”, “artificial intelligence” (broad)
- Stage 2 (learning-based): “machine learning”, “deep learning”, “neural network(s)”
- Stage 3 (LLM/agent-based): “large language model(s)”, “LLM/LLMs”, “AI agent(s)”, “cognitive agent(s)”

The term “natural language processing” was included as it encompasses earlier NLP methods predating modern LLMs and appears frequently in the IEEE Xplore corpus. The MeSH term “Natural Language Processing”[Mesh] was included in the PubMed string to capture indexed NLP studies that may not use the verbatim phrase in abstract text.

**ESRD-related terms** were selected to cover all three principal renal replacement therapy modalities — hemodialysis, peritoneal dialysis, and kidney transplantation — as well as umbrella terms for ESRD/ESKD and renal replacement therapy. Both American (“hemodialysis”) and British (“haemodialysis”) spellings were included to minimize retrieval bias.
