# Supplementary material for: From assistant to collaborator: A systematic review of the evolution of artificial intelligence in end-stage renal disease care and management
Source: PLOS Digit Health. 2026 Sep 2;5(9):e0001635. doi: 10.1371/journal.pdig.0001635 (PMC13537592; doi:10.1371/journal.pdig.0001635)
Supplement: Supplementary file 2 [file pdig.0001635.s002.docx]

# S2 Text. Bibliometric Analysis — Geographic, Institutional, and Author Profiles

*This supplementary section provides the complete geographic distribution, collaboration network, institutional output, and author contribution analyses summarised in the main text (Sections 3.2 and 3.3). All analyses are based on the systematic review corpus of 100 ESRD-focused studies (search period: January 2005 – June 2025).*

## S2.1 Geographic Distribution and Collaboration Landscape

### S2.1.1 Counting Methodology

Country affiliation was counted at the paper level: each paper contributed one count to each country represented among its authors’ institutional affiliations, regardless of the number of contributing authors from that country or the number of institutions involved. A paper with authors from three countries therefore contributed one count to each of those three countries. This approach is consistent with standard practice in bibliometric analyses and reflects geographic participation in the published literature rather than individual author-level representation.

### S2.1.2 Country-Level Output

Across the 100-study systematic review corpus, 35 countries contributed a total of 182 paper-country pairs. The top 10 producing countries — China, the United States, the United Kingdom, Australia, the Netherlands, South Korea, Belgium, Japan, France, and Germany — collectively account for approximately 69.2% (126/182) of all paper-country pairs, reflecting a pronounced geographic concentration. China and the United States function as the dual centers of global output, with China affiliated with 43 studies (23.6%) and the United States with 27 studies (14.8%).

**Table S2-1: Top 10 Countries by Study Affiliation Count (Systematic Review Corpus, n = 100 Studies)**

| Country | Paper-Country Pairs | % of Total (182) |
| --- | --- | --- |
| China | 43 | 23.6% |
| United States | 27 | 14.8% |
| United Kingdom | 15 | 8.2% |
| Australia | 13 | 7.1% |
| Netherlands | 9 | 4.9% |
| South Korea | 9 | 4.9% |
| Belgium | 8 | 4.4% |
| Japan | 7 | 3.8% |
| France | 7 | 3.8% |
| Germany | 7 | 3.8% |

### S2.1.3 Regional Specialization by Treatment Modality

Regional specialization is evident across the three ESRD treatment modalities. China dominates hemodialysis (33.96% of modality paper-country pairs, 18/53) and peritoneal dialysis (47.06%, 16/34), whereas the United States leads kidney transplantation (17.89%, 17/95) with strong European participation from Belgium (6.32%, 6/95) and France (5.26%, 5/95).

### S2.1.4 International Collaboration Network

The international collaboration network was constructed from 131 unique country-pair co-authorship edges spanning 33 collaborating countries. The United States is the most connected node (collaboration weight = 56), followed by the United Kingdom (29), Croatia (29), China (29), and Australia (25). The strongest bilateral collaboration links are: Croatia–United States (5 shared papers), United Kingdom–United States (4), Australia–United States (4), Belgium–Germany (4), Germany–United States (4), and China–United Kingdom (4). European nations (Belgium, France, Germany, Spain, the United Kingdom) form a dense intra-regional research network.

These geographic patterns have direct implications for the generalizability of AI models developed in this field. The concentration of research output in China and the United States, and the relative under-representation of low- and middle-income countries, raises questions about the transferability of models trained on data from high-resource healthcare settings to contexts with different patient demographics, treatment protocols, and data infrastructure.

## S2.2 Institutional Analysis

Conducting institutional output and collaboration analysis helps dissect the research structure and dominant forces in artificial intelligence applications in the end-stage renal disease field. Based on comprehensive institutional statistical analysis of the 100-study systematic review corpus, a total of 372 distinct institution entities participated in research in this field. Notably, 101 institutions (27.2%) have published two or more papers, indicating significant concentration of research activity among leading institutions. The distribution of institution counts and research contributions by institution type is shown in Fig 6a and Fig 6b of the main text, respectively. The breakdown by institution type is as follows: universities, 171 institutions (45.97%), associated with 93 papers (93.0%); hospitals, 136 (36.56%), associated with 67 papers (67.0%); research institutions, 49 (13.17%), associated with 39 papers (39.0%); and companies, 16 (4.30%), associated with 15 papers (15.0%). Note that because a single paper may involve multiple institution types, the number of papers associated with each type sums to more than 100.

Among specific institutional contributors, Icahn School of Medicine at Mount Sinai leads with 6 affiliated studies, reflecting its strength in hemodialysis complication prediction, transplant database modelling, and vascular access assessment. Fresenius Medical Care (United States), KU Leuven, Peking University, Renal Research Institute (United States), Sun Yat-sen University, and The First Affiliated Hospital of Sun Yat-sen University each contributed 5 studies. Institutions affiliated with 4 studies include Assistance Publique–Hôpitaux de Paris, Chang Gung University, Inserm, Johns Hopkins University, Mayo Clinic, the Paris Cardiovascular Research Center, Peking University First Hospital, and Sichuan University. Cross-institutional collaboration is prominent, with the KU Leuven–University Hospitals Leuven partnership and the Inserm Paris Transplant Group–AP-HP consortium together dominating kidney transplantation AI through imaging-pathology multimodal prognostic models of substantial international influence. The top 20 most productive institutions are listed in Table S2-2.

**Table S2-2: Top 20 Institutions by Study Affiliation Count (Systematic Review Corpus, n = 100 Studies)**

| **Institution** | **Count** | **Category** |
| --- | --- | --- |
| Icahn School of Medicine at Mount Sinai | 6 | University |
| Fresenius Medical Care (United States) | 5 | Company |
| KU Leuven | 5 | University |
| Peking University | 5 | University |
| Renal Research Institute (United States) | 5 | Research Institution |
| Sun Yat-sen University | 5 | University |
| The First Affiliated Hospital, Sun Yat-sen Univ. | 5 | Hospital |
| Assistance Publique – Hôpitaux de Paris | 4 | Hospital |
| Chang Gung University | 4 | University |
| Inserm | 4 | Research Institution |
| Johns Hopkins University | 4 | University |
| Mayo Clinic (United States) | 4 | Hospital |
| Paris Cardiovascular Research Center | 4 | Research Institution |
| Peking University First Hospital | 4 | Hospital |
| Sichuan University | 4 | University |
| Army Medical University (China) | 3 | University |
| Centre Hospitalier Universitaire de Tours | 3 | Hospital |
| Coventry University | 3 | University |
| Hôpital Saint-Louis | 3 | Hospital |
| Johns Hopkins Medicine | 3 | Research Institution |

## S2.3 Author Analysis

Author contribution analysis reveals that the AI-in-ESRD field exhibits a characteristic core-periphery collaboration structure. Across the 100-study systematic review corpus, author analysis identifies a small but persistent group of high-frequency contributors who anchor sustained research programs. Authors contributing to three or more studies are listed in Table S2-3. Peter Kotanko (Renal Research Institute, New York; 5 studies) and Maarten Naesens (KU Leuven, Belgium; 5 studies) lead among high-frequency contributors, concentrating respectively on dialysis-related AI prediction models and kidney transplantation outcome modelling. Olivier Aubert and Alexandre Loupy (both Assistance Publique–Hôpitaux de Paris / Inserm / Paris Cardiovascular Research Center, France; 4 studies each) co-authored all four of their respective papers together, forming the “dual core” of French transplant risk prediction research; similarly, Ping Zhang and Jianghua Chen (First Affiliated Hospital, Zhejiang University, China; 4 studies each) maintained full co-authorship across four papers focused on large-cohort transplantation AI. This pattern of close collaboration reinforces research continuity and depth, though it may limit engagement with external contributors.

From a collaboration network perspective, cross-institutional co-authorship is the norm rather than the exception within this corpus. Key structural features include stable “dual-core” dyads within national research groups (e.g., Loupy–Aubert in France; Chen–Zhang in China) and the emergence of three-continent collaboration networks in kidney transplantation (North America–Europe–East Asia). However, representation from the Southern Hemisphere remains limited. Despite strong participation from Australia, contributors from Latin America (Brazil aside), Africa, and other under-represented regions remain largely absent from high-output author groups — an important opportunity for future expansion of international collaboration, data diversity, and health equity.

**Table S2-3: Authors Contributing to Three or More Studies (Systematic Review Corpus, n = 100)**

| **Name** | **Institute** | **Nation** | **Count** |
| --- | --- | --- | --- |
| Peter Kotanko | Icahn School of Medicine at Mount Sinai; Renal Research Institute (United States) | USA | 5 |
| Maarten Naesens | KU Leuven | Belgium | 5 |
| Olivier Aubert | Assistance Publique – Hôpitaux de Paris; Inserm; Paris Cardiovascular Research Center | France | 4 |
| Ping Zhang | First Affiliated Hospital Zhejiang University; Zhejiang University | China | 4 |
| Jianghua Chen | First Affiliated Hospital Zhejiang University; Zhejiang University | China | 4 |
| Alexandre Loupy | Assistance Publique – Hôpitaux de Paris; Inserm; Paris Cardiovascular Research Center | France | 4 |
| Bernard Burke | Coventry University; Health & Life (Taiwan) | UK | 3 |
| Xiao Yang | Sun Yat-sen University; The First Affiliated Hospital, Sun Yat-sen University | China | 3 |
| Nassim Kamar | Inserm; Université Toulouse III – Paul Sabatier | France | 3 |
| Yu Tian | Ministry of Education China; Zhejiang Lab; Zhejiang University | China | 3 |
| Andrew J Bentall | Mayo Clinic (United States) | USA | 3 |
| Mark D Stegall | Mayo Clinic (United States) | USA | 3 |
| Marc Raynaud | Inserm; Paris Cardiovascular Research Center; Université Paris Cité | France | 3 |
| Arun Shroff | Dr. Mohan’s Diabetes Specialities Centre; Flinders Medical Centre; XtendWave (United States) | Australia / India / USA | 3 |
| Babak J. Orandi | New York University; University of Alabama at Birmingham | USA | 3 |
| Sunil Shroff | Dr. Mohan’s Diabetes Specialities Centre; Flinders Medical Centre; XtendWave (United States) | Australia / India / USA | 3 |
| John Larkin | Fresenius Medical Care (United States) | USA | 3 |
| Miklos Z. Molnar | University of Utah | USA | 3 |
| Nithya Krishnan | Coventry University; University Hospitals Coventry and Warwickshire NHS Trust | UK | 3 |
| Len Usvyat | Fresenius Medical Care (United States) | USA | 3 |
| Christophe Legendre | Assistance Publique – Hôpitaux de Paris; Inserm; Université Paris Cité | France | 3 |
| Carmen Lefaucheur | Assistance Publique – Hôpitaux de Paris; Inserm; Paris Cardiovascular Research Center | France | 3 |
| Jingsong Li | Ministry of Education China; Zhejiang Lab; Zhejiang University | China | 3 |
| Zuwen Kuang | Fresenius Medical Care (United States) | USA | 3 |
| Hanjie Zhang | Renal Research Institute (United States) | USA | 3 |
| Hatem Ali | Coventry University; University Hospitals Coventry and Warwickshire NHS Trust | UK | 3 |
| Gillian Divard | Assistance Publique – Hôpitaux de Paris; Inserm; Paris Cardiovascular Research Center | France | 3 |
| Franklin W. Maddux | Fresenius Medical Care (Germany); Fresenius Medical Care (United States) | Germany / USA | 3 |
| David Briggs | Institute of Immunology (Croatia); NHS Blood and Transplant; University of Birmingham | Croatia / UK | 3 |
| Dirk Kuypers | KU Leuven | Belgium | 3 |
