# Supplementary material for: From assistant to collaborator: A systematic review of the evolution of artificial intelligence in end-stage renal disease care and management
Source: PLOS Digit Health. 2026 Sep 2;5(9):e0001635. doi: 10.1371/journal.pdig.0001635 (PMC13537592; doi:10.1371/journal.pdig.0001635)
Supplement: Supplementary file 3 [file pdig.0001635.s003.docx]

# S1 Table. Risk of Bias and Quality Assessment Results for All 100 Included Studies

**Extraction dates:** Data were extracted by two independent reviewers (M.W. and C.H.) between January and June 2025. Discrepancies were resolved through consensus discussion, with arbitration by a third reviewer (Z.H.) where necessary.

**Assessment tools applied:**

- **PROBAST** (Prediction Model Risk of Bias Assessment Tool): applied to prediction model studies. Domains rated as Low / Unclear / High risk of bias.
- **QUADAS-2** (Quality Assessment of Diagnostic Accuracy Studies): applied to studies primarily evaluating a diagnostic test or AI-based classifier. Domains rated as Low / Unclear / High risk of bias.
- **DECIDE-AI** (DEveloping and vAlidating Clinical AI decision support systems): applied to advanced AI-assisted clinical decision support systems. Domains rated as Adequate / Partial / Inadequate.

**Abbreviations:** RoB = Risk of Bias; App = Applicability; Low = Low risk; High = High risk; Unc = Unclear; Adeq = Adequate; Part = Partial; Inad = Inadequate; — = not applicable for this tool.

## Part A: PROBAST Assessments

*n = 74 studies assessed with PROBAST (prediction model studies).*

Domain descriptions: **D1** = Participants; **D2** = Predictors; **D3** = Outcome; **D4** = Analysis.

| # | Study | Treatment Modality | Stage | D1 RoB | D2 RoB | D3 RoB | D4 RoB | Overall RoB |
| --- | --- | --- | --- | --- | --- | --- | --- | --- |
| 1 | A Computer-Based Dialysis Therapy Advisor | Hemodialysis | Stage 1 | High | Low | High | High | High |
| 2 | Building a Normative Decision Support System for Clinical an… | Hemodialysis | Stage 1 | Unclear | Low | Low | High | High |
| 3 | Clinical evaluation of an expert system for arteriovenous fi… | Hemodialysis | Stage 1 | Unclear | Low | Low | High | High |
| 4 | Computerized Decision Support for EPO Dosing in Hemodialysis… | Hemodialysis | Stage 1 | High | High | Low | High | High |
| 5 | Development and exploitation of a clinical decision support … | Hemodialysis | Stage 1 | Low | Low | Low | High | High |
| 6 | The ERA-EDTA cohort study—comparison of methods to predict s… | Hemodialysis | Stage 1 | High | Low | Low | High | High |
| 7 | Fuzzy logic–based clinical decision support system for the e… | Kidney Transplantation | Stage 1 | Unclear | Low | Low | High | High |
| 8 | A novel approach to dry weight adjustments for dialysis pati… | Hemodialysis | Stage 2 | Low | Unclear | High | High | High |
| 9 | An optimized machine learning framework for predicting intra… | Hemodialysis | Stage 2 | Low | Low | Unclear | High | High |
| 10 | Application of deep learning to predict the low serum albumi… | Hemodialysis | Stage 2 | High | Unclear | Low | High | High |
| 11 | Artificial intelligence supported anemia control system (AIS… | Hemodialysis | Stage 2 | High | Low | Unclear | High | High |
| 12 | Artificial-Intelligence-Based Prediction of Clinical Events … | Hemodialysis | Stage 2 | High | High | Low | High | High |
| 13 | Boosted machine learning model for predicting intradialytic … | Hemodialysis | Stage 2 | Unclear | Low | Low | High | High |
| 14 | Deep Learning Model for Real - Time Prediction of Intradialy… | Hemodialysis | Stage 2 | Unclear | Low | Low | High | High |
| 15 | DeepVAQ : an adaptive deep learning for prediction of vascul… | Hemodialysis | Stage 2 | High | High | Unclear | High | High |
| 16 | Machine Learning Analysis of Time - Dependent Features for P… | Hemodialysis | Stage 2 | High | Low | Unclear | High | High |
| 17 | Machine learning approaches for the mortality risk assessmen… | Hemodialysis | Stage 2 | Low | Low | Low | High | High |
| 18 | Machine learning-based prediction of cerebral hemorrhage in … | Hemodialysis | Stage 2 | Unclear | Low | Low | High | High |
| 19 | Machine learning-based risk prediction model for arterioveno… | Hemodialysis | Stage 2 | Unclear | High | Unclear | High | High |
| 20 | Optimization of Dry Weight Assessment in Hemodialysis Patien… | Hemodialysis | Stage 2 | Low | Low | Unclear | High | High |
| 21 | Pattern recognition and prognostic analysis of longitudinal … | Hemodialysis | Stage 2 | High | Unclear | Low | High | High |
| 22 | Personalized prediction of intradialytic hypotension in clin… | Hemodialysis | Stage 2 | Low | High | Low | High | High |
| 23 | Predicting 3‑year all‑cause mortality in patients undergoing… | Hemodialysis | Stage 2 | High | Low | Low | High | High |
| 24 | Predicting dry weight change in Hemodialysis patients using … | Hemodialysis | Stage 2 | Low | Low | Low | High | High |
| 25 | Predicting high-flow arteriovenous fistulas and cardiac outc… | Hemodialysis | Stage 2 | Unclear | Low | Low | High | High |
| 26 | Prediction of dialysis adequacy using data - driven machine … | Hemodialysis | Stage 2 | Low | Low | Low | Low | Low |
| 27 | Prediction of intradialytic hypotension using pre - dialysis… | Hemodialysis | Stage 2 | Low | Low | Low | High | High |
| 28 | Prediction of mortality in hemodialysis patients based on au… | Hemodialysis | Stage 2 | High | Low | Low | High | High |
| 29 | Prediction the prognosis of the poisoned patients undergoing… | Hemodialysis | Stage 2 | High | Low | Low | High | High |
| 30 | Predictors of shorter - and longer - term mortality after CO… | Hemodialysis | Stage 2 | Low | Low | Low | High | High |
| 31 | Prognostic Machine Learning Models for First-Year Mortality … | Hemodialysis | Stage 2 | Low | Low | Low | High | High |
| 32 | Real-time prediction of intradialytic hypotension using mach… | Hemodialysis | Stage 2 | Unclear | Low | Low | Unclear | High |
| 33 | Real-time prediction of intradialytic relative blood volume:… | Hemodialysis | Stage 2 | Unclear | Low | Low | High | High |
| 34 | Time - series deep survival prediction for hemodialysis pati… | Hemodialysis | Stage 2 | Unclear | Low | Low | High | High |
| 35 | Use machine learning to help identify possible sarcopenia ca… | Hemodialysis | Stage 2 | Unclear | Low | Unclear | High | High |
| 36 | Attention-Based Deep Learning Model for Prediction of Major … | Peritoneal Dialysis | Stage 2 | Unclear | Low | Unclear | High | High |
| 37 | Biomarker and clinical data–based predictor tool (MAUXI) for… | Peritoneal Dialysis | Stage 2 | Low | Low | Low | High | High |
| 38 | Construction and validation of a predictive model for the ri… | Peritoneal Dialysis | Stage 2 | Low | Low | Low | Low | Low |
| 39 | Derivation and Validation of Risk Scores to Predict Cerebrov… | Peritoneal Dialysis | Stage 2 | Low | Low | Low | Low | Low |
| 40 | Explainable machine learning algorithm to predict cardiovasc… | Peritoneal Dialysis | Stage 2 | Low | Low | Unclear | High | High |
| 41 | Machine learning algorithms for the prediction of adverse pr… | Peritoneal Dialysis | Stage 2 | High | Low | Low | High | High |
| 42 | Machine learning for identification of short-term all-cause … | Peritoneal Dialysis | Stage 2 | Unclear | Low | Low | High | High |
| 43 | Machine learning model and nomogram to predict the risk of h… | Peritoneal Dialysis | Stage 2 | Low | Low | High | High | High |
| 44 | Mortality Prediction with Adaptive Feature Importance Recali… | Peritoneal Dialysis | Stage 2 | Low | Low | Low | High | High |
| 45 | Optimizing home visits through machine learning for preventi… | Peritoneal Dialysis | Stage 2 | High | Unclear | Unclear | High | High |
| 46 | Predicting Prolonged Length of Hospital Stay for Peritoneal … | Peritoneal Dialysis | Stage 2 | Low | Low | Low | Low | Low |
| 47 | Random forest can accurately predict the technique failure o… | Peritoneal Dialysis | Stage 2 | Low | Low | Low | Low | Low |
| 48 | Risk prediction of cardiovascular events in peritoneal dialy… | Peritoneal Dialysis | Stage 2 | High | Low | Unclear | High | High |
| 49 | Understanding the immune signature fingerprint of peritoneal… | Peritoneal Dialysis | Stage 2 | Unclear | Low | Low | High | High |
| 50 | Unveiling risk factors: a prognostic model of frequent perit… | Peritoneal Dialysis | Stage 2 | High | Low | Low | High | High |
| 51 | Vibrational Spectroscopy of Peritoneal Dialysis Effluent for… | Peritoneal Dialysis | Stage 2 | Unclear | Low | Low | High | High |
| 52 | Visit-to-visit blood pressure variability and clinical outco… | Peritoneal Dialysis | Stage 2 | High | Low | Unclear | High | High |
| 53 | A Machine Learning - Driven Virtual Biopsy System For Kidney… | Kidney Transplantation | Stage 2 | Low | Low | Low | High | High |
| 54 | A Machine Learning Prediction Model for Immediate Graft Func… | Kidney Transplantation | Stage 2 | Low | Low | Low | High | High |
| 55 | A dynamic model for predicting graft function in kidney reci… | Kidney Transplantation | Stage 2 | Unclear | Low | Low | High | High |
| 56 | An integrated machine learning model enhances delayed graft … | Kidney Transplantation | Stage 2 | Low | Low | Low | High | High |
| 57 | Artificial Intelligence—A Tool for Risk Assessment of Delaye… | Kidney Transplantation | Stage 2 | Low | Low | Low | High | High |
| 58 | Artificial intelligence assisted risk prediction in organ tr… | Kidney Transplantation | Stage 2 | Low | Low | Low | High | High |
| 59 | Characterizing hub biomarkers for post-transplant renal fibr… | Kidney Transplantation | Stage 2 | Unclear | High | Unclear | High | High |
| 60 | Data - Driven Chronic Allograft Phenotypes: A Novel and Vali… | Kidney Transplantation | Stage 2 | Low | Low | Low | High | High |
| 61 | Deep learning identified pathological abnormalities predicti… | Kidney Transplantation | Stage 2 | Low | Low | Low | High | High |
| 62 | Dynamic prediction of renal survival among deeply phenotyped… | Kidney Transplantation | Stage 2 | Low | Low | Low | Low | Low |
| 63 | Forecasting of Patient - Specific Kidney Transplant Function… | Kidney Transplantation | Stage 2 | Unclear | Low | Low | High | High |
| 64 | Improved survival prediction for kidney transplant outcomes … | Kidney Transplantation | Stage 2 | Unclear | Low | Low | High | High |
| 65 | Live-Donor Kidney Transplant Outcome Prediction (L-TOP) usin… | Kidney Transplantation | Stage 2 | High | Low | Low | High | High |
| 66 | Machine Learning Support for Decision - Making in Kidney Tra… | Kidney Transplantation | Stage 2 | Low | Low | Low | High | High |
| 67 | Machine learning does not outperform traditional statistical… | Kidney Transplantation | Stage 2 | Low | Low | Low | Low | Low |
| 68 | Predicting Kidney Graft Survival Using Machine Learning Meth… | Kidney Transplantation | Stage 2 | High | Low | Low | High | High |
| 69 | Predicting Kidney Transplant Survival using Multiple Feature… | Kidney Transplantation | Stage 2 | High | Low | Low | High | High |
| 70 | Predicting graft survival in paediatric kidney transplant re… | Kidney Transplantation | Stage 2 | Low | High | Low | High | High |
| 71 | Predicting prognostic factors in kidney transplantation usin… | Kidney Transplantation | Stage 2 | Low | Low | Low | High | High |
| 72 | The role of artificial intelligence measured preoperative ki… | Kidney Transplantation | Stage 2 | Unclear | Low | Low | High | High |
| 73 | The study of the association between immune monitoring and p… | Kidney Transplantation | Stage 2 | High | Low | Low | High | High |
| 74 | Using machine learning and an ensemble of methods to predict… | Kidney Transplantation | Stage 2 | Low | Unclear | Low | High | High |

## Part B: QUADAS-2 Assessments

*n = 18 studies assessed with QUADAS-2 (diagnostic accuracy studies).*

Domain descriptions: **D1** = Patient Selection; **D2** = Index Test; **D3** = Reference Standard; **D4** = Flow and Timing.

| # | Study | Treatment Modality | Stage | D1 RoB | D2 RoB | D3 RoB | D4 RoB | Overall RoB |
| --- | --- | --- | --- | --- | --- | --- | --- | --- |
| 1 | A Rule-Based Decision-Making Diagnosis System to Evaluate Ar… | Hemodialysis | Stage 1 | Unclear | High | Unclear | Unclear | High |
| 2 | Classification of arteriovenous fistula sounds using a convo… | Hemodialysis | Stage 2 | Unclear | High | Unclear | Unclear | High |
| 3 | Deep learning to classify arteriovenous access aneurysms in … | Hemodialysis | Stage 2 | Low | High | High | Unclear | High |
| 4 | Evaluation of Hemodialysis Arteriovenous Bruit by Deep Learn… | Hemodialysis | Stage 2 | Low | High | High | Unclear | High |
| 5 | Automated lung ultrasound image assessment using artificial … | Peritoneal Dialysis | Stage 2 | Unclear | Unclear | Unclear | Unclear | High |
| 6 | Machine-learning algorithms define pathogen-specific local i… | Peritoneal Dialysis | Stage 2 | Unclear | High | Low | Unclear | High |
| 7 | Recent advances in novel diagnostic testing for peritoneal d… | Peritoneal Dialysis | Stage 2 | High | High | High | High | High |
| 8 | Automated assessment of glomerulosclerosis and tubular atrop… | Kidney Transplantation | Stage 2 | Unclear | Unclear | Low | Unclear | High |
| 9 | Automated evaluation with deep learning of total interstitia… | Kidney Transplantation | Stage 2 | Low | High | Low | Low | High |
| 10 | Computer-Aided Diagnostic System for Early Detection of Acut… | Kidney Transplantation | Stage 2 | Unclear | High | Unclear | Low | High |
| 11 | Convolutional Neural Networks for the Evaluation of Chronic … | Kidney Transplantation | Stage 2 | Low | Low | High | Low | High |
| 12 | Deep Learning Global Glomerulosclerosis in Transplant Kidney… | Kidney Transplantation | Stage 2 | Low | Low | Low | Low | Low |
| 13 | Deep learning - based classification of kidney transplant pa… | Kidney Transplantation | Stage 2 | Unclear | High | Unclear | Low | High |
| 14 | Deep learning-enabled classification of kidney allograft rej… | Kidney Transplantation | Stage 2 | High | Unclear | Low | High | High |
| 15 | Diagnosis and classification of kidney transplant rejection … | Kidney Transplantation | Stage 2 | High | High | Low | Unclear | High |
| 16 | Image Analysis Pipeline for Renal Allograft Evaluation and F… | Kidney Transplantation | Stage 2 | Unclear | High | High | Low | High |
| 17 | RtNet: a deep hybrid neural network for the identification o… | Kidney Transplantation | Stage 2 | High | High | High | High | High |
| 18 | exKidneyBERT: a language model for kidney transplant patholo… | Kidney Transplantation | Stage 3 | Unclear | Low | Low | Unclear | Unclear |

## Part C: DECIDE-AI Assessments

*n = 8 studies assessed with DECIDE-AI (AI decision support systems).*

Domain descriptions: **D1** = Transparency; **D2** = Clinical Representativeness; **D3** = Safety Reporting; **D4** = Human-AI Integration. Ratings: Adequate / Partial / Inadequate.

| # | Study | Treatment Modality | Stage | D1 | D2 | D3 | D4 | Overall Quality |
| --- | --- | --- | --- | --- | --- | --- | --- | --- |
| 1 | Developing Decision Support for Dialysis Treatment of Chroni… | Hemodialysis | Stage 1 | - | - | - | - | - |
| 2 | Translation of evidence into kidney transplant clinical prac… | Kidney Transplantation | Stage 1 | - | - | - | - | - |
| 3 | Application of ChatGPT to Support Nutritional Recommendation… | Hemodialysis | Stage 3 | Partial | Partial | Partial | Inadequate | Inadequate |
| 4 | Enhancing nutritional management in peritoneal dialysis pati… | Peritoneal Dialysis | Stage 3 | Partial | Adequate | Inadequate | Partial | Inadequate |
| 5 | ChatGPT Solving Complex Kidney Transplant Cases: A Comparati… | Kidney Transplantation | Stage 3 | Partial | Partial | Inadequate | Partial | Inadequate |
| 6 | Insights into ChatGPT application in renal transplantation | Kidney Transplantation | Stage 3 | Partial | Partial | Partial | Partial | Partial |
| 7 | Trials and Tribulations: Responses of ChatGPT to Patient Que… | Kidney Transplantation | Stage 3 | Partial | Adequate | Adequate | Partial | Partial |
| 8 | Using ChatGPT for Kidney Transplantation: Perceived Informat… | Kidney Transplantation | Stage 3 | Partial | Adequate | Inadequate | Inadequate | Inadequate |

## Part D: Inter-Rater Agreement Summary (Table S1-D)

*The following table summarises domain-level inter-rater percentage agreement and Cohen’s κ for each assessment tool, based on the 100-study corpus. Per Cochrane Handbook v6.3 Section 8.4.3, weighted kappa with quadratic weights (κ_w) is also reported as an overall summary statistic for each tool given the ordinal nature of the ratings.*

| Tool | Domain | Description | N Studies | Agreement (%) | Cohen’s κ | Interpretation |
| --- | --- | --- | --- | --- | --- | --- |
| PROBAST | D1 | Participants (sampling, representativeness) | 74 | 47.3 | 0.169 | Slight/Poor |
| PROBAST | D2 | Predictors (definition, measurement, blinding) | 74 | 71.6 | 0.158 | Slight/Poor‡ |
| PROBAST | D3 | Outcome (definition, measurement) | 74 | 71.6 | 0.231 | Fair‡ |
| PROBAST | D4 | Analysis (modeling, validation, performance) | 74 | 86.5 | 0.411 | Moderate |
| QUADAS-2 | D1 | Patient Selection (spectrum, consecutive enrollment) | 18 | 27.8 | -0.040 | Slight/Poor† |
| QUADAS-2 | D2 | Index Test (test conduct, interpretation) | 18 | 66.7 | 0.176 | Slight/Poor‡ |
| QUADAS-2 | D3 | Reference Standard (conduct, interpretation) | 18 | 66.7 | 0.500 | Moderate |
| QUADAS-2 | D4 | Flow and Timing (patient flow, time interval) | 18 | 27.8 | -0.009 | Slight/Poor† |
| DECIDE-AI | D1 | Transparency (system description, explainability) | 6 | 100.0 | — | Perfect agreement (κ undefined) |
| DECIDE-AI | D2 | Clinical Representativeness (population, setting) | 6 | 83.3 | 0.714 | Substantial |
| DECIDE-AI | D3 | Safety Reporting (adverse events, monitoring) | 6 | 100.0 | 1.000 | Almost Perfect |
| DECIDE-AI | D4 | Human-AI Integration (workflow, oversight) | 6 | 16.7 | -0.364 | Slight/Poor†‡ |
| **PROBAST** | **Pooled κ_w** | *Quadratic-weighted (296 domain pairs)* | *(74 × 4)* | 69.3 | **0.58** | **Moderate** |
| **QUADAS-2** | **Pooled κ_w** | *Quadratic-weighted (72 domain pairs)* | *(18 × 4)* | 50.0 | **0.56** | **Moderate** |
| **DECIDE-AI** | **Pooled κ_w** | *Quadratic-weighted (24 domain pairs)* | *(6 × 4)* | 75.0 | **0.72** | **Substantial** |

*† Negative κ values indicate observed agreement below chance expectancy; this occurs when one category dominates the rating distribution.* *‡ Kappa Paradox: κ suppressed by distributional skewness (>70% of ratings in one category); percentage agreement is the more informative metric in these cases (Feinstein & Cicchetti, 1990).* *κ = Cohen’s unweighted kappa; κ_w = pooled weighted kappa with quadratic weights (Cohen, 1968; Cochrane Handbook v6.3 §8.4.3).* *Interpretation: Poor <0.20, Fair 0.21–0.40, Moderate 0.41–0.60, Substantial 0.61–0.80, Almost Perfect >0.80 (Landis & Koch, 1977).*
