# Supplementary material for: From assistant to collaborator: A systematic review of the evolution of artificial intelligence in end-stage renal disease care and management
Source: PLOS Digit Health. 2026 Sep 2;5(9):e0001635. doi: 10.1371/journal.pdig.0001635 (PMC13537592; doi:10.1371/journal.pdig.0001635)
Supplement: Supplementary file 4 [file pdig.0001635.s005.docx]

# S1 PRISMA Checklist. PRISMA 2020 Checklist

**Manuscript:** From Assistant to Collaborator: A Systematic Review of the Evolution of Artificial Intelligence in End-Stage Renal Disease Care and Management

*Completed in accordance with Page MJ, McKenzie JE, Bossuyt PM, Boutron I, Hoffmann TC, Mulrow CD, et al. The PRISMA 2020 statement: an updated guideline for reporting systematic reviews. BMJ. 2021;372:n71. Locations are given as manuscript sections and supporting information files rather than page numbers, because pagination changes during typesetting.*

| **Section and Topic** | **Item #** | **Checklist item** | **Location where item is reported** |
| --- | --- | --- | --- |
| **TITLE** |  |  |  |
| Title | 1 | Identify the report as a systematic review. | Title page (title states “A Systematic Review”) |
| **ABSTRACT** |  |  |  |
| Abstract | 2 | See the PRISMA 2020 for Abstracts checklist. | Abstract |
| **INTRODUCTION** |  |  |  |
| Rationale | 3 | Describe the rationale for the review in the context of existing knowledge. | Introduction, paragraphs 1–3 |
| Objectives | 4 | Provide an explicit statement of the objective(s) or question(s) the review addresses. | Introduction, final paragraph; Abstract |
| **METHODS** |  |  |  |
| Eligibility criteria | 5 | Specify the inclusion and exclusion criteria for the review and how studies were grouped for the syntheses. | Methods, Section 2.3; grouping criteria in Section 2.7 |
| Information sources | 6 | Specify all databases, registers, websites, organisations, reference lists and other sources searched or consulted to identify studies. Specify the date when each source was last searched or consulted. | Methods, Section 2.2 (PubMed, IEEE Xplore, Web of Science; January 2005 – June 2025); S1 Text |
| Search strategy | 7 | Present the full search strategies for all databases, registers and websites, including any filters and limits used. | Methods, Section 2.2; complete verbatim search strings in S1 Text |
| Selection process | 8 | Specify the methods used to decide whether a study met the inclusion criteria of the review, including how many reviewers screened each record and each report retrieved, whether they worked independently, and if applicable, details of automation tools used in the process. | Methods, Section 2.4 (two independent reviewers, blinded; third-reviewer adjudication; no automation tools used) |
| Data collection process | 9 | Specify the methods used to collect data from reports, including how many reviewers collected data from each report, whether they worked independently, any processes for obtaining or confirming data from study investigators, and if applicable, details of automation tools used in the process. | Methods, Section 2.4 (duplicate extraction using a standardised form; study investigators were not contacted) |
| Data items — outcomes | 10a | List and define all outcomes for which data were sought. Specify whether all results that were compatible with each outcome domain in each study were sought, and if not, the methods used to decide which results to collect. | Methods, Sections 2.4 and 2.8; reported performance metrics for every included study are listed in Extended Table 1 |
| Data items — other variables | 10b | List and define all other variables for which data were sought. Describe any assumptions made about any missing or unclear information. | Methods, Sections 2.4, 2.6, 2.7 and 2.8 (no assumptions or imputation; unreported items recorded as “not reported”) |
| Study risk of bias assessment | 11 | Specify the methods used to assess risk of bias in the included studies, including details of the tool(s) used, how many reviewers assessed each study and whether they worked independently, and if applicable, details of automation tools used in the process. | Methods, Section 2.5 (PROBAST, QUADAS-2, DECIDE-AI; two independent reviewers; consensus and third-reviewer arbitration); S1 Table |
| Effect measures | 12 | Specify for each outcome the effect measure(s) (e.g. risk ratio, mean difference) used in the synthesis or presentation of results. | Methods, Section 2.8; discrimination and classification metrics as reported by each primary study (AUC, C-index, accuracy, sensitivity, specificity, F1), presented without pooling; Extended Table 1 |
| Synthesis methods — eligibility for synthesis | 13a | Describe the processes used to decide which studies were eligible for each synthesis (e.g. tabulating the study intervention characteristics and comparing against the planned groups for each synthesis). | Methods, Sections 2.6 and 2.7 (studies grouped a priori by developmental stage, application domain and treatment modality) |
| Synthesis methods — data preparation | 13b | Describe any methods required to prepare the data for presentation or synthesis, such as handling of missing summary statistics, or data conversions. | Methods, Section 2.8 (no imputation or conversion; unreported values retained as “not reported” and excluded from computation) |
| Synthesis methods — tabulation and visual display | 13c | Describe any methods used to tabulate or visually display results of individual studies and syntheses. | Methods, Section 2.6; Results and Sections 4–6 (Extended Table 1, Tables 2–7, Figs 8–14) |
| Synthesis methods — synthesis of results | 13d | Describe any methods used to synthesize results and provide a rationale for the choice(s). If meta-analysis was performed, describe the model(s), method(s) to identify the presence and extent of statistical heterogeneity, and software package(s) used. | Methods, Section 2.8 (structured narrative synthesis with ranges and medians; meta-analysis not undertaken, with rationale); Section 9.4 |
| Synthesis methods — heterogeneity | 13e | Describe any methods used to explore possible causes of heterogeneity among study results (e.g. subgroup analysis, meta-regression). | Methods, Sections 2.6–2.8 (pre-specified subgroup description by stage, application domain and treatment modality); no meta-regression was possible |
| Synthesis methods — sensitivity analyses | 13f | Describe any methods used to assess robustness of the synthesized results. | Not applicable — no statistical synthesis was performed (Methods, Section 2.8; Section 9.4) |
| Reporting bias assessment | 14 | Describe any methods used to assess risk of bias due to missing results in a synthesis (arising from reporting biases). | Not assessed — formal small-study or publication-bias testing requires pooled effect estimates, which were not computed; the associated limitation is stated in Sections 9.2 and 9.4 |
| Certainty assessment | 15 | Describe any methods used to assess certainty (or confidence) in the body of evidence for an outcome. | GRADE was not applied; certainty is characterised descriptively through the tool-specific appraisal framework in Methods, Section 2.5 and discussed in Sections 8.1 and 9.4 |
| **RESULTS** |  |  |  |
| Study selection | 16a | Describe the results of the search and selection process, from the number of records identified in the search to the number of studies included in the review, ideally using a flow diagram. | Results, first paragraph; Fig 1 (PRISMA 2020 flow diagram) |
| Study selection — exclusions | 16b | Cite studies that might appear to meet the inclusion criteria, but which were excluded, and explain why they were excluded. | Results, first paragraph (952 full-text exclusions by category); complete numbered listing with reason for each record in S1 Data |
| Study characteristics | 17 | Cite each included study and present its characteristics. | Extended Table 1 (all 100 included studies); Sections 4 and 5; Tables 2 and 3 |
| Risk of bias in studies | 18 | Present assessments of risk of bias for each included study. | Methods, Section 2.5 (summary); study-level domain ratings for all 100 studies in S1 Table |
| Results of individual studies | 19 | For all outcomes, present, for each study: (a) summary statistics for each group (where appropriate) and (b) an effect estimate and its precision (e.g. confidence/credible interval), ideally using structured tables or plots. | Extended Table 1 (per-study best method, performance metrics, data scale and validation approach); Table 3. Precision estimates are reported only where the primary study reported them; their frequent absence is discussed in Methods, Section 2.8 |
| Results of syntheses — contributing studies | 20a | For each synthesis, briefly summarise the characteristics and risk of bias among contributing studies. | Sections 4 and 5 (per domain and per developmental stage); Methods, Section 2.5; S1 Table |
| Results of syntheses — synthesis results | 20b | Present results of all statistical syntheses conducted. If meta-analysis was done, present for each the summary estimate and its precision (e.g. confidence/credible interval) and measures of statistical heterogeneity. If comparing groups, describe the direction of the effect. | Sections 4, 5 and 8.1 (narrative ranges and medians only; no pooled estimates were computed) |
| Results of syntheses — heterogeneity | 20c | Present results of all investigations of possible causes of heterogeneity among study results. | Sections 5 and 8.1 (differences by developmental stage, application domain, treatment modality, sample size and validation strategy); Figs 10–13 |
| Results of syntheses — sensitivity analyses | 20d | Present results of all sensitivity analyses conducted to assess the robustness of the synthesized results. | Not applicable — no statistical synthesis was performed |
| Reporting biases | 21 | Present assessments of risk of bias due to missing results (arising from reporting biases) for each synthesis assessed. | Not assessed; the systematic under-reporting of calibration, prospective validation and fairness metrics across the evidence base is reported in Methods, Section 2.8 and discussed in Section 8.1 |
| Certainty of evidence | 22 | Present assessments of certainty (or confidence) in the body of evidence for each outcome assessed. | Sections 8.1 and 9.4; S1 Table |
| **DISCUSSION** |  |  |  |
| Discussion — interpretation | 23a | Provide a general interpretation of the results in the context of other evidence. | Discussion, Section 8.1 |
| Discussion — limitations of evidence | 23b | Discuss any limitations of the evidence included in the review. | Discussion, Sections 8.1 and 8.2; Limitations, Sections 9.3 and 9.4 |
| Discussion — limitations of review processes | 23c | Discuss any limitations of the review processes used. | Limitations, Sections 9.1, 9.2, 9.5 and 9.6 |
| Discussion — implications | 23d | Discuss implications of the results for practice, policy, and future research. | Discussion, Section 8.3; Section 7; Conclusion |
| **OTHER INFORMATION** |  |  |  |
| Registration and protocol | 24a | Provide registration information for the review, including register name and registration number, or state that the review was not registered. | Methods, Section 2.1 — the review was not registered in a prospective registry such as PROSPERO; also stated in Section 9.6 |
| Registration and protocol — access | 24b | Indicate where the review protocol can be accessed, or state that a protocol was not prepared. | Methods, Section 2.1 — a public protocol was not prepared; eligibility criteria, search strategy, data-extraction items and the three-stage analytical framework were specified a priori before screening began |
| Registration and protocol — amendments | 24c | Describe and explain any amendments to information provided at registration or in the protocol. | Not applicable — no amendments were made to the a priori plan (Methods, Section 2.1) |
| Support | 25 | Describe sources of financial or non-financial support for the review, and the role of the funders or sponsors in the review. | Funding statement, title page |
| Competing interests | 26 | Declare any competing interests of review authors. | Declaration of Interest Statement, title page |
| Availability of data, code and other materials | 27 | Report which of the following are publicly available and where they can be found: template data collection forms; data extracted from included studies; data used for all analyses; analytic code; any other materials used in the review. | Data availability statement, title page. Search strings: S1 Text. Bibliometric analysis methods and outputs: S2 Text. Extracted quality-appraisal data for all included studies: S1 Table. Full-text exclusion dataset: S1 Data. Extracted study-level characteristics and performance data: Extended Table 1 |

*For more information, visit: http://www.prisma-statement.org/*
