# Supplementary material for: From assistant to collaborator: A systematic review of the evolution of artificial intelligence in end-stage renal disease care and management
Source: PLOS Digit Health. 2026 Sep 2;5(9):e0001635. doi: 10.1371/journal.pdig.0001635 (PMC13537592; doi:10.1371/journal.pdig.0001635)
Supplement: Supplementary file 6 [file pdig.0001635.s008.docx]

# Response to Reviewers — Second Revision

**Manuscript ID:** PDIG-D-25-01132R1

**Title:** From Assistant to Collaborator: A Systematic Review of the Evolution of Artificial Intelligence in End-Stage Renal Disease Care and Management

**Journal:** PLOS Digital Health

Dear Editors and Reviewers,

We are grateful for the opportunity to submit a further revision of our manuscript, and we thank the reviewers for their continued careful reading and their constructive follow-up comments. We were pleased to see that both reviewers considered our previous responses satisfactory, and we have now addressed each of the additional points raised in this round. Below we respond to every comment in turn. For each item we indicate what was changed and where; the corresponding before-and-after text is provided in the accompanying “Revised Manuscript with Track Changes.” We believe these changes have improved the accuracy, balance, and transparency of the manuscript.

# Reviewer #1

## Major Point 1

**Comment:** The authors state that all 100 included studies directly involve patients with ESRD receiving hemodialysis, peritoneal dialysis, or kidney transplantation. However, some of the Stage 3 examples cited in the text appear to be general medical AI systems rather than ESRD-specific studies. Please explain and reconcile.

**Response:**

We thank the reviewer for this careful reading, which identified a genuine inconsistency in our presentation of Stage 3. We have re-examined the corpus study by study and can confirm that all 100 included studies—those listed in Extended Table 1—do involve ESRD populations. The source of the apparent contradiction was that Section 5.3 cited several systems from the broader biomedical AI literature (notably ColaCare, MDAgents, Gaber et al.’s retrieval-augmented workflow, and Liu et al.’s MedGen) alongside the corpus studies, without making clear that these systems are contextual citations rather than members of the 100-study corpus. We also found, on re-checking, that the methodological breakdown previously reported in Section 5.3.3 mistakenly attributed two multi-agent studies and one retrieval-augmented study to the ESRD corpus; this was an error, since none of the seven ESRD-specific Stage 3 studies use those architectures.

We have corrected this throughout Section 5.3 and reconciled the two categories of evidence as follows:

1. We added an explicit statement at the start of Section 5.3.1 distinguishing (i) the seven ESRD-specific Stage 3 studies that form part of the corpus (Extended Table 1; refs 64, 65, 67, 68, 95, 140, 141), all of which apply general-purpose or domain-adapted large language models to ESRD patients, from (ii) multi-agent and retrieval-augmented systems drawn from the broader biomedical AI literature (e.g., ColaCare, MDAgents, MMedAgent, MedGen), which were not developed in ESRD populations, are not counted among the 100 studies, and are cited only to contextualize the methodological trajectory.
2. We corrected Section 5.3.3 to state accurately that all seven ESRD-specific Stage 3 studies are large language model applications (six general-purpose conversational/multimodal models and one domain-adapted biomedical language model), and that no ESRD-specific study implemented a multi-agent or retrieval-augmented architecture.
3. We revised Sections 5.3.2, 5.3.4, and 5.4.1 so that every non-ESRD system (ColaCare on MIMIC-IV, MDAgents, Gaber, MedGen) is now explicitly flagged as broader-literature context rather than ESRD-specific evidence. We added a footnote to Table 5 making the same distinction, and we similarly clarified in Section 4.3.3 that the Claude 3.5-Sonnet discharge-summary study (ref 69), whose population was patients with “renal insufficiency” rather than strictly ESRD, is cited as contextual evidence and is not part of the corpus.

These changes preserve the integrity of the claim that the 100-study corpus is entirely ESRD-specific, while making transparent where we draw on the wider literature for context. We are grateful to the reviewer for prompting this correction.

## Major Point 2

**Comment:** DECIDE-AI is primarily a reporting guideline/framework for the early-stage evaluation of AI decision-support systems, not a risk-of-bias tool in the traditional sense. Please avoid describing it as a risk-of-bias (ROB) tool.

**Response:**

We fully agree and apologize for the imprecise characterization. DECIDE-AI is indeed a reporting and evaluation guideline for the early-stage clinical evaluation of AI-driven decision-support systems, not a conventional risk-of-bias instrument. We have corrected this in every location where the framework is described:

- Section 2.5 now states explicitly that PROBAST and QUADAS-2 were used for formal risk-of-bias assessment, whereas DECIDE-AI was applied as a structured framework to appraise reporting completeness (transparency, clinical representativeness, safety reporting, and human–AI integration), with ratings labeled as Adequate, Partial, or Inadequate reporting rather than risk-of-bias categories. We have also added a brief rationale clarifying that DECIDE-AI was chosen for this subset because the AI-assisted decision-support applications concerned are neither prediction-model nor diagnostic-accuracy studies and therefore fall outside the intended scope of PROBAST and QUADAS-2.
- The Data Availability statement and Inclusion Criteria item 5 have been reworded so that DECIDE-AI is described as a reporting-quality evaluation rather than a risk-of-bias tool.

These edits ensure the manuscript characterizes each instrument according to its intended purpose.

## Major Point 3

**Comment:** Thank you for providing inter-rater agreement. The tool-selection agreement was κ = 0.87, and the authors then state that stage-assignment agreement was quantified as part of the tool-selection kappa. However, tool selection and stage assignment are not the same thing.

**Response:**

The reviewer is correct, and we have separated the two. Tool selection is driven by study design type (prediction model, diagnostic accuracy study, or decision-support system), whereas stage assignment is driven by the nature of the primary AI methodology; these are conceptually distinct classification steps, and conflating their agreement statistics was inappropriate. We have now computed inter-rater agreement for stage assignment independently. Across the 99 study pairs with valid data from both reviewers, the two reviewers agreed on the developmental stage for 96 of 99 studies, corresponding to Cohen’s κ = 0.90 (almost perfect agreement). Section 2.7 now reports this statistic separately and explains clearly that it must not be conflated with the tool-selection agreement (κ = 0.87) reported in Section 2.5. The Figure 9 legend has been updated accordingly to cite the stage-assignment value (κ = 0.90, Section 2.7).

## Major Point 4

**Comment:** The manuscript reports an observed agreement range of 28%–67% for QUADAS-2, which indicates disagreement in some domains. Could the authors discuss the implications for the credibility of the assessment, and how final consensus was reached?

**Response:**

We thank the reviewer for raising this important point and have added a dedicated discussion to Section 2.5. We make several clarifications. First, the QUADAS-2 estimate (observed agreement 27.8%–66.7%) is based on a small subset of only 18 diagnostic accuracy studies (72 domain-level observation pairs), which makes domain-level agreement statistics inherently unstable and sensitive to a few discordant ratings. Second, several QUADAS-2 domains—particularly patient selection and flow and timing—require finer judgments about study conduct that were frequently reported incompletely in the primary studies, producing greater initial divergence at the independent-rating stage. Third, these figures describe the difficulty of independent first-pass appraisal, not the reliability of the final assessments. Regarding how consensus was reached: for every domain in which the two reviewers initially diverged (in both QUADAS-2 and PROBAST), they jointly re-examined the primary article against the relevant signaling questions, discussed the basis for each judgment, and revised their ratings to a single agreed classification; where agreement could not be reached, a third reviewer (Z.H.) adjudicated the final rating before it entered the synthesis. The ratings reported in Table S1 and used throughout the manuscript therefore reflect adjudicated consensus judgments rather than single-reviewer scores, so the residual inter-rater divergence does not propagate into the reported results. Finally, and importantly for the credibility question, study quality was not used as an eligibility criterion and the quality-appraisal results were not used to weight, rank, or exclude studies in any analysis (Section 2.3); the appraisal is reported descriptively to characterize the quality of the evidence base, so any residual uncertainty in individual domain ratings does not affect the substantive findings or conclusions of the review. We have retained the observed-agreement figures in the interest of transparency, so that readers can appraise the subjectivity inherent in applying these instruments to a heterogeneous AI literature.

## Major Point 5

**Comment:** Please soften the wording in the manuscript. Avoid terms such as “breakthrough” and “excellent,” which appear relatively frequently, and use neutral language.

**Response:**

We agree and have systematically revised the manuscript to remove promotional language and adopt a neutral, measured tone throughout. We screened the full text for the flagged terms and their close synonyms and replaced each rhetorical use as follows: “breakthrough” to “notable advance” or “important/notable progress”; “excellent/excellently/outstanding/excelled” to “strong,” “good,” “well,” or “high” (for example, “excellent performance” to “strong performance,” and “outstanding sensitivity” to “high sensitivity”); “remarkable/tremendous” to “notable/considerable”; “superior” to “high,” “greater,” or “improved” where it was used rhetorically; “revolutionary” to “promising” or “significant”; and “unprecedented” to “notable.” We also softened rhetorical uses of “significantly” that did not refer to a statistical test (for example, “significantly enhances the accuracy” to “can enhance the accuracy,” and “significantly improving training efficiency” to “improving training efficiency”). Section headings were revised accordingly (for example, “Adaptive Excellence” became “Adaptive Performance,” and “Breakthrough Performance through Data-Driven Learning” became “Improved Performance through Data-Driven Learning”). The specific sentence-level changes are listed in the Track Changes document and in the accompanying modification record. The substance of the findings is unchanged; the revised text reports them in measured, neutral terms.

## Major Point 6

**Comment:** The authors now state that the review followed PRISMA 2020. Was the review registered with a protocol?

**Response:**

We thank the reviewer for this question and have addressed it directly. The review was not registered in a prospective registry such as PROSPERO. We have added a transparent statement to Section 2.1 noting this, and clarifying that, to preserve methodological rigor in the absence of registration, the eligibility criteria, search strategy, data-extraction items, and three-stage analytical framework were all specified a priori—before title-and-abstract screening—and applied without subsequent modification. We have also added the absence of prospective registration as an explicit limitation (Section 9.6).

## Major Point 7

**Comment:** Please avoid statements such as “AI can significantly improve medical efficiency” unless the claim is clearly supported by the included studies.

**Response:**

We agree. This sentence in the Introduction has been rewritten to remove the unsupported causal claim. The revised text presents improved efficiency and easing of resource constraints as a potential benefit that motivates ongoing investigation but that requires rigorous clinical evaluation before it can be considered established, rather than as a demonstrated outcome. We also neutralized related phrasing elsewhere (for example, “significantly improving patients’ nutritional status” and “significantly improving patients’ health literacy” were softened to indicate potential rather than established effects).

## Minor Point 1

**Comment:** Please standardize terminology; “AI in ESRD,” “ESRD care,” and “ESRD management” should not be used interchangeably unless the authors intend a distinction and explain it.

**Response:**

We have added a clarifying statement to the Introduction immediately after the definition of “AI in ESRD.” It specifies that “ESRD care,” “ESRD management,” “ESRD care and management,” and “ESRD diagnosis and treatment management” are used interchangeably as an inclusive umbrella for the full clinical continuum (risk prediction, diagnosis, treatment, and longitudinal monitoring) and are not intended to denote distinct sub-domains, and that wherever a specific clinical activity is meant (for example, diagnostic support, or monitoring and management), it is named explicitly. This makes our usage deliberate and transparent.

## Minor Point 2

**Comment:** AUC-ROC and AUROC should be defined consistently. Please avoid switching between the two terms.

**Response:**

We have standardized the main text to use “AUC” (area under the receiver operating characteristic curve) consistently; the instance previously written as “AUC-ROC” in Section 4.4 and the instance written as “AUROC” in Section 5.3.4 have both been changed to “AUC.” We added a brief note in Section 4.4 stating that the metric is referred to as AUC throughout the text, and that where individual primary studies reported the value as AUROC, that original notation is retained in Extended Table 1 and Table 3, since AUC and AUROC denote the same quantity. This avoids switching between terms in the narrative while preserving fidelity to the original studies’ reporting.

## Minor Point 3

**Comment:** Please define all abbreviations in the table footnotes.

**Response:**

We have added comprehensive abbreviation footnotes to Table 3 and Extended Table 1, defining every metric and method abbreviation that appears in each table (for Table 3: AI, AUC, ACC, SENS, SPEC, PRE, RECALL, F1, Dice, RCT, CNN, RNN, XGBoost, GPT; for Extended Table 1, additionally AUPRC, C-index, IBS/IBC, IoU, RMSE, MSE, MAE, R², LSTM, MLP, KNN, SVM, PCA, KELM and its metaheuristic-optimized variants, and BERT, among others). We also reviewed Tables 2, 4, 5, 6, and 7 and added footnotes defining the abbreviations that appear in each (e.g., AI, ESRD, HD, PD, KT, LLM, RAG, CoT, EHR, HLA).

## Minor Point 4

**Comment:** What does N/A mean in Table 3? Not applicable? Not evaluated? Not reported?

**Response:**

Thank you for flagging this ambiguity. N/A denotes “not applicable.” We have added an explicit definition to the Table 3 footnotes: N/A indicates that the study did not report a conventional classification performance metric (such as AUC) because its primary outcome was a prospective clinical endpoint rather than an algorithmic classification metric (the relevant cell is Jin et al. 2024, a prospective randomized controlled trial whose primary outcome was serum prealbumin improvement). For completeness, we also clarified in the footnote that a dash (–) denotes a value not reported in the original study, distinguishing the two notations.

# Reviewer #4

**Comment:** I recommend that the authors further clarify the inclusion criteria, because not all dialysis patients are ESRD patients—conditions such as acute kidney injury may also require dialysis. Please specify whether eligible studies included only ESRD patients receiving dialysis, and explain how non-ESRD dialysis populations were excluded.

**Response:**

We thank the reviewer for this important clarification, which we fully agree is essential for the validity of an ESRD-focused review. We have revised the eligibility criteria and added an explanatory statement to Section 2.3:

1. Inclusion criterion 1 now specifies that eligible studies were those whose population clearly and predominantly comprised patients with established ESRD—chronic, irreversible kidney failure requiring long-term renal replacement therapy—and that studies were eligible only where renal replacement therapy was administered for ESRD rather than for a potentially reversible condition. Where a study reported a mixed dialysis population, it was included only if ESRD patients constituted the analyzed cohort or a clearly separable subgroup.
2. Exclusion criterion 1 now explicitly excludes studies in which dialysis was provided for acute kidney injury (AKI) or other potentially reversible indications, as well as studies in which ESRD patients could not be distinguished from non-ESRD dialysis populations (e.g., undifferentiated AKI cohorts or pooled acute/chronic cohorts).

These additions make the ESRD-specific scope of the review explicit and document the procedure used to exclude non-ESRD dialysis populations.

# Editorial and Journal Items

We note the editor’s reminders regarding image preparation (NAAS tool), ORCID verification, and optional protocol deposition. All figures will be processed through the PLOS NAAS tool and uploaded in the required formats, and we will confirm ORCID verification for all co-authors before final acceptance. As this is a systematic review of previously published literature, there is no wet-laboratory protocol to deposit on protocols.io; however, the complete search strings (Supplementary S1), the full list of 952 excluded studies with reasons (Supplementary S2), the per-study quality appraisal results (Table S1), and the bibliometric analysis scripts and outputs (Supplementary S3) are provided to support full reproducibility.

We thank the editors and reviewers once again for their thorough and constructive evaluation. We believe the revised manuscript resolves each of the points raised and is substantially clearer and better calibrated as a result. We remain happy to provide any further clarification.

Sincerely,

Mohan Wang, Caogen Hong, Zhengxing Huang, Yue Gu, and Fengmin Shao
