# Supplementary material for: From assistant to collaborator: A systematic review of the evolution of artificial intelligence in end-stage renal disease care and management
Source: PLOS Digit Health. 2026 Sep 2;5(9):e0001635. doi: 10.1371/journal.pdig.0001635 (PMC13537592; doi:10.1371/journal.pdig.0001635)
Supplement: Supplementary file 7 [file pdig.0001635.s007.docx]

# Response to Reviewers — Consolidated Response Letter

**Title:** From Assistant to Collaborator: A Systematic Review of the Evolution of Artificial Intelligence in End-Stage Renal Disease Care and Management **Journal:** PLOS Digital Health

Dear Editor and Reviewers,

We sincerely thank the editor and all reviewers for their thorough and constructive feedback. We have carefully addressed every comment and have substantially revised the manuscript accordingly. Below we provide point-by-point responses organized by reviewer.

# Reviewer #1

## R1-1

**Original Comment:**

The manuscript states that 124 studies were included after screening, yet some sections analyze larger number of studies (ie 1028 publications in keyword analysis). Can the authors comment and explicitly define/separate datasets used for a) systematic review synthesis vs 2) bibliometric analysis. If bibliometric analysis was conducted, this must be prespecified in the methods section.

**Response:**

We thank the reviewer for this observation. The original manuscript did not clearly distinguish between the two analytical scopes, which we agree was confusing.

A new subsection, Section 2.6 (“Analytical Datasets”), has been added to the Methods. It explicitly defines two datasets:

1. **Systematic review corpus** (100 ESRD-focused studies): used for all stage-level, domain-level, modality-level, and performance analyses (Sections 4–5), as well as the quality and bias assessment (Section 2.5).
2. **Bibliometric corpus** (1,052 full-text-assessed records): used for annual publication trends, geographic and institutional distribution, and keyword trajectory analyses (Section 3).

Each section header in the revised manuscript now specifies which dataset is being analyzed. We believe this resolves the ambiguity the reviewer identified.

## R1-2

**Original Comment:**

Inclusion criteria require studies to involve ESRD patients receiving dialysis or transplantation (lines 160–163). However, several included studies appear to involve CKD screening, population-level risk detection, or non-ESRD cohorts, yet are classified under “Kidney Transplantation” or “ESRD care” in Extended Table 1. Can the authors explain this? Is this a misclassification?

**Response:**

We thank the reviewer for this careful observation. On systematic re-examination of the included corpus, we excluded all studies involving CKD screening, population-level risk detection, or non-ESRD cohorts that did not meet the strict ESRD patient criterion. In addition, we identified 20 general medical AI studies that had been incorrectly retained in the original version; as these studies do not involve ESRD-specific patient populations, they do not satisfy the inclusion criteria and should not have been placed in the systematic review corpus. These studies have been removed at the full-text screening stage in the revised PRISMA flow. The corpus has been refined to 100 studies, all of which directly involve ESRD patients undergoing hemodialysis (n = 40), peritoneal dialysis (n = 21), or kidney transplantation (n = 39). All PRISMA flow numbers, statistical analyses, and narrative descriptions throughout the manuscript have been updated accordingly.

## R1-3

**Original Comment:**

While databases and general keyword domains are listed, full search strings are not provided for any database. Recommend adding full-database specific search strategy as a supplement.

**Response:**

We agree that the full search strings are essential for reproducibility. The complete, database-specific search strategies for PubMed, IEEE Xplore, and Web of Science have been compiled into a new supplementary document (Supplementary S1). Section 2.2 of the Methods now includes a cross-reference to this supplement.

## R1-4

**Original Comment:**

In the 2-stage screening process, how many of the authors reviewed the study? Was this done independently? How were disagreements resolved? This needs to be explicitly stated.

**Response:**

Section 2.4 has been revised to explicitly state: “Both stages were performed independently by two reviewers (M.W. and C.H.), each blinded to the other’s decisions. Disagreements at each stage were resolved through structured discussion, with unresolved cases adjudicated by a third reviewer (Z.H.). Data extraction was conducted in duplicate using a standardized extraction form.”

## R1-5

**Original Comment:**

Similarly, please provide inter-rate agreement.

**Response:**

We have formally assessed inter-rater agreement at two levels across all 100 included studies. First, agreement on tool selection yielded Cohen’s kappa κ = 0.87 (almost perfect agreement; Landis and Koch, 1977). Second, domain-level agreement was calculated separately for each tool: PROBAST 47.3%–86.5% (74 studies, 296 domain-level pairs); QUADAS-2 27.8%–66.7% (18 studies, 72 pairs); DECIDE-AI pooled weighted kappa κ_w = 0.72 (substantial agreement; 8 studies). Full details are reported in Section 2.5 and Table S1.

## R1-6

**Original Comment:**

The review repeatedly comments on lack of validation/interpretability, yet no structured risk-of-bias or quality appraisal tool is described. Recommend adding formal assessment tool.

**Response:**

We fully agree. A new Section 2.5 (Quality and Bias Assessment) has been added, describing a tool-specific quality and risk-of-bias assessment framework: PROBAST for prediction model studies (74 studies), QUADAS-2 for diagnostic accuracy studies (18 studies), and DECIDE-AI for clinical decision support systems (8 studies). Full per-study, per-domain results are provided in Table S1.

## R1-7

**Original Comment:**

What do the authors mean by “high quality” studies? Please define this terminology.

**Response:**

We thank the reviewer for flagging this ambiguity. In the revised manuscript, Inclusion Criteria item 5 now clarifies that “high quality” was not applied as an independent eligibility criterion. All peer-reviewed original research articles meeting criteria 1–4 were assessed for inclusion. Methodological rigor was instead evaluated post-hoc through the structured quality and bias assessment framework described in Section 2.5 (PROBAST, QUADAS-2, or DECIDE-AI).

## R1-8

**Original Comment:**

The results reported ~24% of studies performed external validation. Can the authors also define what qualifies as external (ie temporal split? public dataset)?

**Response:**

Section 5.2.5 now includes a three-part operational definition: external validation is defined as evaluation of model performance on data that are structurally independent of the training source — specifically: (a) an independent cohort from a different institution or geographic region; (b) a publicly available benchmark dataset; or (c) a prospective or temporal split collected at a different site. Same-source temporal holdout splits are not counted as external validation.

## R1-9

**Original Comment:**

The three-stage evolutionary framework is central to the manuscript but lacks explicit criteria for assigning studies to stages. What was the rubric/criteria? Please justify.

**Response:**

A new Section 2.7 (Stage Classification Criteria) has been added. The criteria are defined operationally based on the primary AI component’s relationship to data: Stage 1 requires fully human-authored, data-independent decision logic; Stage 2 requires model parameters learned from empirical data; Stage 3 requires a large-scale pre-trained language model as the primary reasoning component. Rules for mixed-methodology studies and inter-rater agreement (κ = 0.87) are also specified.

## R1-10

**Original Comment:**

Statements linking publication surges to COVID-19 or shifts in clinical priorities are speculative and not supported by formal analyses. Please temper and rephrase descriptive observations.

**Response:**

We agree. All speculative attributions linking publication surges to specific external events have been removed from Section 3.1. The revised text is restricted to observations directly supported by the data (e.g., annual counts, phase boundaries) without inferring causation.

## R1-11

**Original Comment:**

Terms such as “AI-ESRD,” “AI in ESRD,” and “renal AI systems” are used interchangeably without definition. Would suggest to standardize to lessen any confusion.

**Response:**

All occurrences of “AI-ESRD” and “renal AI systems” have been replaced with the standardized form “AI in ESRD.” An explicit defining statement has been added in the Introduction: “Throughout this review, the term ‘AI in ESRD’ is used as a collective designation for all artificial intelligence applications in the context of ESRD care and management, encompassing rule-based, machine learning, deep learning, and large language model approaches applied across hemodialysis, peritoneal dialysis, and kidney transplantation.”

## R1-12

**Original Comment:**

Authors reported “441 country affiliations” and top-10 country dominance. This is very difficult to interpret. Were affiliations counted per author? paper? or per institution?

**Response:**

Section 3.2 now explicitly states the counting method: “country affiliation was counted at the paper level: each paper contributed one count to each country represented among its authors’ institutional affiliations, regardless of the number of contributing authors from that country. A paper with authors from three countries therefore contributed one count to each of those three countries.” The updated analysis (100-study corpus) reports 35 countries contributing 182 paper-country pairs.

## R1-13

**Original Comment:**

The authors restricted to english peer-reviewed literature. What was the basis? Please provide justification.

**Response:**

Section 2.3, item 4 now provides the justification: “The restriction to English-language literature reflects the review team’s capacity for systematic and rigorous linguistic appraisal. While this criterion may exclude relevant studies published in other languages, it ensures consistency of evaluation across included studies.” The limitation is explicitly acknowledged in the Limitations section (Section 9.1).

## R1-14

**Original Comment:**

The abstract highlights critical need to bridge gap between modelling and clinical utility. This is reasonable. But, elsewhere in the manuscript, it seems to imply an inevitable progression toward cognitive collaborators w/o even acknowledging that much of stage 3 remains unvalidated. Please tighten what your evidence supports.

**Response:**

Three converging changes have been made. First, the Section 5.3 heading now reads “Large Language Model- and Agent-Based Systems — An Emerging Research Direction.” Second, Section 5.3.1 opens with evidential caveats: only 7 of 100 ESRD studies employed Stage 3 approaches, and findings are described as “preliminary observations on an active research frontier, not settled conclusions.” Third, Section 5.3.3 has been corrected to report proportions based on the 7 ESRD-specific studies (LLMs: 4/7, 57.1%; multi-agent: 2/7, 28.6%; RAG: 1/7, 14.3%).

## R1-M-1

**Original Comment:**

Consider adding raw counts alongside % throughout the results for clarity.

**Response:**

We have revised the relevant passages in Sections 3.2, 3.3, 5.2.6, and 5.4.7 to include raw counts in the form (n/total) alongside each percentage. Percentages representing individual study performance metrics have been left unchanged, as adding a fraction would misrepresent the nature of those figures.

## R1-M-2

**Original Comment:**

Figure legends should explicitly state which dataset is displayed. Figure titles should also be stand-alone descriptions for the data presented.

**Response:**

All figure legends have been updated to include an explicit statement of the underlying dataset and sufficient contextual information to be read independently of the main text. Revised legends for Figures 9 and 12 are provided with full stage-level study counts, interpretive caveats for Stage 3, and original-work attribution.

## R1-M-3

**Original Comment:**

Always define acronyms at first mention.

**Response:**

A full pass was conducted to verify all acronyms are defined at first mention. Expansions were added or corrected for 17+ acronyms including PRISMA, PROBAST, AUC, SVM, CNN, RNN, LLM, GPU, EHR, SHAP, LIME, KNN, MLP, AUROC, KDPI, ERA-EDTA, FDA, and NMPA.

## R1-M-4

**Original Comment:**

Initial retrieval was 5,000+ records, not 1072. Please check and explain.

**Response:**

In the original submission, the initial database retrieval count was incompletely reported. Following a complete PRISMA 2020 screening process, the correct flow is: PubMed (n = 1,323) + IEEE Xplore (n = 469) + Web of Science (n = 1,438) = 3,230 total records → 932 duplicates removed → 2,298 screened → 1,246 excluded → 1,052 full-text assessed → 952 excluded → 100 included. This is now reported correctly in the revised Results section and Figure 1.

# Reviewer #2

## R2-1

**Original Comment:**

In my perspective, the manuscript presents several methodological and structural issues that need to be addressed to ensure the rigor and transparency expected of a systematic review. […] while keywords are provided, the complete search equations used across all databases are missing […] the authors must explicitly state how many reviewers performed the screening and data extraction processes. Critically, there is no assessment of the risk of bias or quality appraisal of the included studies […] Overall, the research questions appear to be more suited to a scoping review than a systematic review.

**Response:**

We thank the reviewer for this substantive critique. All four specific concerns have been addressed:

(a) **Complete search strings**: Supplementary S1 now provides the full database-specific search strategies.
(b) **English-only justification**: Section 2.3, item 4 provides the rationale.
(c) **Reviewer numbers**: Section 2.4 specifies two independent reviewers (M.W. and C.H.) with third-reviewer adjudication (Z.H.).
(d) **Bias assessment**: Section 2.5 introduces PROBAST, QUADAS-2, and DECIDE-AI as a three-tool quality assessment framework.

Regarding whether this work constitutes a systematic review or scoping review: we respectfully note that the study adheres to PRISMA 2020, includes a PICO framework, predefined inclusion/exclusion criteria, dual-reviewer screening, and now formal bias appraisal — elements that collectively satisfy the methodological requirements of a systematic review.

## R2-2

**Original Comment:**

While the framework is innovative, its presentation could benefit from a more explicit definition of the boundaries and transitions between stages, particularly regarding the criteria for moving from one stage to the next.

**Response:**

Section 2.7 (Stage Classification Criteria) defines boundaries by a single underlying criterion — the nature of the primary AI component’s relationship to data — rather than by time periods or performance thresholds. This makes boundaries reproducible and allows studies from any era to be classified without recourse to publication date.

## R2-3

**Original Comment:**

The manuscript should expand on the limitations of current models in real-world settings, including issues related to overfitting, data drift, and the need for multicenter validation. It would be beneficial to elaborate on practical strategies for translating AI research into routine clinical practice […] Also, a more thorough discussion of regulatory frameworks, patient privacy, and equity in AI deployment would strengthen the manuscript.

**Response:**

A new Section 8 (Discussion) has been added with three subsections: 8.1 Performance versus Practice (examining the 97.6% internal / 26.5% external / 0% prospective validation landscape); 8.2 Structural Barriers (regulatory — FDA/EMA/NMPA; technical — EHR heterogeneity, real-time inference; equity — documented racial disparities in AI prediction quality); and 8.3 Translational Pathways (multicenter prospective validation, early regulatory engagement, human-in-the-loop design, equitable training data).

## R2-4

**Original Comment:**

Finally, the document is quite lengthy on some parts and would benefit significantly from being synthesized and streamlined to improve clarity, and the conclusion section assumes an unusual structure that should be revised.

**Response:**

**On length**: Section 3 has been condensed by approximately 60%, with institutional and author analyses relocated to Supplementary S3. The Introduction has been streamlined per R4-1.

**On conclusion structure**: The original RQ1–RQ6 sub-headed structure has been replaced with four integrated narrative paragraphs organized around scope/summary, three-stage empirical findings, the performance–practice gap, and forward-looking implications.

## R2-M-1

**Original Comment:**

British vs American English is mixed (e.g., modelling vs modeling)

**Response:**

A systematic search identified and replaced all British English spellings with American English equivalents (11 distinct terms including haemorrhage → hemorrhage, characterised → characterized, modelling → modeling, etc.). Reference 154 retains “modelling” in its published title to avoid bibliographic inaccuracy.

# Reviewer #3

## R3-1

**Original Comment:**

The abstract states 23.8% of Stage 2 models lack external validation, but Section 5.2.5 reports 78.8% employed formal validation with 23.8% having external validation. This appears contradictory and needs clarification.

**Response:**

We thank the reviewer for identifying this inconsistency. The contradictory phrasing has been corrected. The abstract now reads: models “achieve clinically relevant metrics (e.g., AUC 0.80–0.90) but often lack external validation,” avoiding the ambiguous percentage. The Discussion (Section 8.1) provides the updated validation landscape: 97.6% of Stage 2 studies relied on internal validation, only 26.5% included external validation, and none reported prospective clinical validation.

## R3-2

**Original Comment:**

Only 12 studies represent Stage 3, yet the framework positions this as a major evolutionary stage comparable to the others. Half of these studies lack formal validation […] consider whether it should be presented as an emerging direction rather than an established stage.

**Response:**

We accept this critique. Three changes have been made: (1) Section 5.3 heading now reads “An Emerging Research Direction”; (2) Section 5.3.1 foregrounds evidential limitations (7/100 ESRD studies, not 12 as in the reviewer’s note which reflected an earlier version); (3) Section 5.3.3 reports proportions exclusively on the 7 ESRD-specific studies. The three-stage framework is retained as it describes a trajectory well-documented in broader AI literature, but the ESRD-specific evidence is no longer overstated.

## R3-3

**Original Comment:**

Multiple performance metrics are reported (AUC, accuracy, sensitivity, specificity, F1, C-index) without clear rationale for metric selection […] Create a standardized performance summary table organized by application domain and treatment modality.

**Response:**

A new Section 4.4 and Table 3 have been added. The section opens with a paragraph explaining the methodological basis for metric diversity (binary classification → AUC; survival analysis → C-index; segmentation → Dice/IoU; LLM tasks → clinical endpoints). Table 3 presents one representative best-performing study for each of nine application domain × treatment modality cells, using AUC as the primary comparator where available and annotating exceptions.

## R3-4

**Original Comment:**

The gap between model performance and clinical implementation is acknowledged but not deeply explored. What specific barriers prevent translation? More discussion is needed on why high-performing models (AUC 0.80-0.90) aren’t being adopted in clinical practice.

**Response:**

Section 8.1 now directly addresses this. It examines single-center retrospective dataset biases, data drift in ESRD (varying treatment protocols, fluid management thresholds across institutions), overfitting risk (Stage 2 median 641 cases), and the systemic conflation of benchmark performance with clinical readiness. The subsection concludes that multicenter prospective validation must become a routine expectation.

## R3-5

**Original Comment:**

The search strategy appears appropriate but there’s no mention of searching clinical trial registries or grey literature. Were conference proceedings included? The exclusion of non-English studies may introduce language bias that should be acknowledged.

## R3-6

**Original Comment:**

The methods section does not specify whether data extractions were done in duplicate or how disagreements were resolved. Was study quality formally assessed using a validated tool?

**Response:**

Both concerns have been addressed. Section 2.4 states data extraction was conducted in duplicate; Section 2.5 introduces the three-tool quality assessment framework. Full domain-level results for all 100 studies are in Table S1.

## R3-7

**Original Comment:**

The results section is very long, and sections 3.1-3.7 (bibliometric analysis) feel disconnected from the core three-stage framework. Consider moving some bibliometric details to supplementary materials.

**Response:**

Section 3 has been substantially restructured to focus directly on evidence supporting the three-stage framework. Section 3.1 (Annual Publication Trends, bibliometric corpus n=1,052) documents the three developmental phases and their alignment with the Stage 1–3 trajectory. Section 3.2 (Geographic and Collaboration Landscape, systematic review corpus n=100) has been condensed to a single paragraph reporting geographic concentration, regional specialization by modality, and collaboration patterns, with implications for model generalizability; full data are in Supplementary S3. Section 3.3 (Institutional and Author Profiles) has been reduced to a brief summary sentence; the complete analysis has been relocated to Supplementary S3. Section 3.4 (Keyword Analysis, bibliometric corpus n=1,052) provides convergent bibliometric corroboration of the three-stage framework. The revised Section 3 is approximately 55% shorter than the original.

## R3-8

**Original Comment:**

Figure 9 is conceptually strong but visually dense, and Figure 12 tries to convey too much information simultaneously. Several figures have small text that may be difficult to read in print.

**Response:**

Figure 9 has been redesigned as a clean 3-column layout with four concise bullet points per stage and a horizontal timeline arrow. Figure 12 has been restructured as a two-panel figure: Panel A shows performance/interpretability trajectories; Panel B shows human-machine relationship evolution. Both have been redrawn at high resolution with legible font sizes.

# Reviewer #4

## R4-1

**Original Comment:**

Introduction: Readable but structurally repetitive with unnecessary prolongation of justifications across the paragraphs (ESRD complexity, AI advantages, gaps in prior review).

**Response:**

Paragraphs 3–8 of the original Introduction have been condensed into two focused paragraphs. The first identifies the two substantive gaps in existing literature; the second states the review’s response through the three-stage framework and six research questions (absorbed into a summary statement rather than enumerated individually). Repetitive “breaking through traditional frameworks” language has been removed.

## R4-2

**Original Comment:**

The authors made an unverified claim about the AI development timeline […] should cite references […] provide the criteria used to assign studies to the 3 stages […] clarify what qualifies as high quality studies.

**Response:**

(a) **Timeline references**: Section 2.2 now includes four concrete milestones: Krizhevsky et al. (2012) for deep learning in image recognition [Ref 167], ML adoption in clinical research [Ref 168], GPT-3 (2020) [Ref 169], and GPT-4 (2023) [Ref 170].
(b) **Stage criteria**: New Section 2.7 with operational definitions (see R1-9).
(c) **“High quality”**: Clarified per R1-7 response.

## R4-3

**Original Comment:**

(a) “Although growth rate has somewhat slowed, the output scale has reached new heights” — must cite or remove. (b) Too much focus on institution and author analyses. (c) Metrics inconsistent across domains. (d) Need comparative table of best-performing models.

**Response:**

(a) The unsupported growth rate claim has been removed.
(b) Institutional and author analyses have been relocated to Supplementary S3, with only a concise summary in the main text (Section 3.3).
(c) Section 4.4 explains the metric diversity as an inherent property of the underlying literature (different task types require different metrics).
(d) Table 3 has been added with consistent selection rules across nine domain × modality cells.

## R4-4

**Original Comment:**

There is no dedicated discussion section.

**Response:**

A full Discussion section (Section 8) has been added with three subsections addressing the performance–practice gap, structural barriers to translation (regulatory, technical, equity), and translational pathways.

## R4-5

**Original Comment:**

There is no dedicated limitation section.

**Response:**

A dedicated Limitations section (Section 9) has been added with five explicitly labeled limitations: (1) language bias; (2) grey literature not searched; (3) limited Stage 3 ESRD-specific studies (n = 7); (4) absence of meta-analysis; (5) cross-sectional scope.

# Reviewer #5

**Original Comment:**

I did appreciate the review and the figure presentation. The methology is correct and is providing a way to understand the data at a glance.

**Response:**

We sincerely thank Reviewer #5 for this supportive assessment. The revised manuscript preserves the visual and methodological approach while addressing the structural and analytical gaps identified by the other reviewers.

# Journal Requirements

## JR-1

**Requirement:** Provide detailed Financial Disclosure with author initials and funder roles.

**Response:**

The manuscript states: “This work was supported by the Major Science and Technology Project of Henan Province (Grant No. 241100310100) awarded to F.S. The funders had no role in study design, data collection and analysis, decision to publish, or preparation of the manuscript.”

## JR-2

**Requirement:** Ensure funders and grant numbers match between Financial Disclosure and Funding Information tab.

**Response:**

We will verify consistency during the online submission process.

## JR-3

**Requirement:** Update Competing Interests statement.

**Response:**

The manuscript states: “The authors declare that they have no known competing financial interests or personal relationships that could have appeared to influence the work reported in this paper.” The online submission form will use PLOS’s standard wording.

## JR-4

**Requirement:** Provide excluded studies table, extracted data table, bias assessments.

**Response:**

- **Supplementary S2**: Numbered table of 952 excluded studies with exclusion reasons.
- **Extended Table 1**: Full extracted data for all 100 included studies.
- **Table S1**: Per-study, per-domain risk-of-bias assessment results (PROBAST, QUADAS-2, DECIDE-AI).
- Data extraction was conducted by M.W. and C.H. between January and June 2025.

## JR-5

**Requirement:** Provide separate .tif or .eps figure files under 10MB each.

**Response:**

All figures will be converted to the required format and verified for size limits before submission.

## JR-6

**Requirement:** Confirm Figures 8, 9, 12 are hand-drawn; provide Figure 3 map base layer source.

**Response:**

**Figures 8, 9, and 12:** All icons, diagrams, and graphical elements were drawn entirely by hand by the corresponding author, Mohan Wang. No third-party material was used. These are wholly original works compatible with CC BY 4.0.

**Figure 3:** The geographic base map was obtained from Natural Earth (http://www.naturalearthdata.com), which releases all data into the public domain. Attribution has been added to the Figure 3 legend: “Map base layer sourced from Natural Earth (http://www.naturalearthdata.com; public domain).”

## JR-7

**Requirement:** Evaluate any recommended citations for relevance.

**Response:**

No specific citations were recommended by the reviewers. We have verified that all references in the revised manuscript are current and relevant.

We thank the editor and reviewers once again for their thorough and constructive review. We believe the revised manuscript addresses all concerns raised and represents a substantially stronger contribution. We remain available to provide any further clarification.

Sincerely,

Mohan Wang, Caogen Hong, Zhengxing Huang, Yue Gu, Fengmin Shao
