# Supplementary material for: From assistant to collaborator: A systematic review of the evolution of artificial intelligence in end-stage renal disease care and management
Source: PLOS Digit Health. 2026 Sep 2;5(9):e0001635. doi: 10.1371/journal.pdig.0001635 (PMC13537592; doi:10.1371/journal.pdig.0001635)
Supplement: Peer review history

# Peer review: Decision Letter 0

**DOI:** 10.1371/journal.pdig.0001635.r001

**Authors:** Sandra Barteit, Kezhi Li

17 Feb 2026

Response to Reviewers Revised Manuscript with Track Changes Manuscript **Journal Requirements:**

1. Please provide a detailed online Financial Disclosure statement. This is published with the article. It must therefore be completed in full sentences and contain the exact wording you wish to be published.

a) State the initials, alongside each funding source, of each author to receive each grant. For example: “This work was supported by the National Institutes of Health (####### to AM; ###### to CJ) and the National Science Foundation (###### to AM).”

b) State what role the funders took in the study. If the funders had no role in your study, please state: “The funders had no role in study design, data collection and analysis, decision to publish, or preparation of the manuscript.”

For more information, please go to our submission guidelines:

https://journals.plos.org/digitalhealth/s/submission-guidelines#loc-financial-disclosure-statement

2. Please ensure that the funders and grant numbers match between the Financial Disclosure field and the Funding Information tab in your submission form. Note that the funders must be provided in the same order in both places as well.

3. Please update your online Competing Interests statement. If you have no competing interests to declare, please state: "The authors have declared that no competing interests exist."

4. As required by our policy on Data Availability, please ensure your manuscript or supplementary information includes the following:

A numbered table of all studies identified in the literature search, including those that were excluded from the analyses.

For every excluded study, the table should list the reason(s) for exclusion.

If any of the included studies are unpublished, include a link (URL) to the primary source or detailed information about how the content can be accessed.

A table of all data extracted from the primary research sources for the systematic review and/or meta-analysis. The table must include the following information for each study:

Name of data extractors and date of data extraction

Confirmation that the study was eligible to be included in the review.

All data extracted from each study for the reported systematic review and/or meta-analysis that would be needed to replicate your analyses.

If data or supporting information were obtained from another source (e.g. correspondence with the author of the original research article), please provide the source of data and dates on which the data/information were obtained by your research group.

If applicable for your analysis, a table showing the completed risk of bias and quality/certainty assessments for each study or outcome.  Please ensure this is provided for each domain or parameter assessed. For example, if you used the Cochrane risk-of-bias tool for randomized trials, provide answers to each of the signalling questions for each study. If you used GRADE to assess certainty of evidence, provide judgements about each of the quality of evidence factor. This should be provided for each outcome.

An explanation of how missing data were handled.

This information can be included in the main text, supplementary information, or relevant data repository. Please note that providing these underlying data is a requirement for publication in this journal, and if these data are not provided your manuscript might be rejected.

5. Please provide separate main figure files in .tif or .eps format only and ensure that all files are under our size limit of 10MB. You may leave the embedded figures in the manuscript.

For more information about how to convert your figure files please see our guidelines: https://journals.plos.org/digitalhealth/s/figures

6. Some material included in your submission may be copyrighted. According to PLOS’s copyright policy, authors who use figures or other material (e.g., graphics, clipart, maps) from another author or copyright holder must demonstrate or obtain permission to publish this material under the Creative Commons Attribution 4.0 International (CC BY 4.0) License used by PLOS journals. Please closely review the details of PLOS’s copyright requirements here: PLOS Licenses and Copyright. If you need to request permissions from a copyright holder, you may use PLOS's Copyright Content Permission form.

Please respond directly to this email or email the journal office and provide any known details concerning your material's license terms and permissions required for reuse, even if you have not yet obtained copyright permissions or are unsure of your material's copyright compatibility.

Potential Copyright Issues:

Figures 8, 9 and 12: Please confirm whether you drew the images / clip-art within the figure panels by hand. If you did not draw the images, please provide (a) a link to the source of the images or icons and their license / terms of use; or (b) written permission from the copyright holder to publish the images or icons under our CC-BY 4.0 license. Alternatively, you may replace the images with open source alternatives. See these open source resources you may use to replace images / clip-art:

- https://commons.wikimedia.org

- https://openclipart.org/

Figure 3: please (a) provide a direct link to the base layer of the map (i.e., the country or region border shape) and ensure this is also included in the figure legend; and (b) provide a link to the terms of use / license information for the base layer image or shapefile. We cannot publish proprietary or copyrighted maps (e.g. Google Maps, Mapquest) and the terms of use for your map base layer must be compatible with our CC-BY 4.0 license.

Note: if you created the map in a software program like R or ArcGIS, please locate and indicate the source of the basemap shapefile onto which data has been plotted.

If your map was obtained from a copyrighted source please amend the figure so that the base map used is from an openly available source. Alternatively, please provide explicit written permission from the copyright holder granting you the right to publish the material under our CC-BY 4.0 license.

Please note that the following CC BY licenses are compatible with PLOS license: CC BY 4.0, CC BY 2.0 and CC BY 3.0, meanwhile such licenses as CC BY-ND 3.0 and others are not compatible due to additional restrictions.

If you are unsure whether you can use a map or not, please do reach out and we will be able to help you. The following websites are good examples of where you can source open access or public domain maps:

* U.S. Geological Survey (USGS) - All maps are in the public domain. (http://www.usgs.gov)

* PlaniGlobe - All maps are published under a Creative Commons license so please cite “PlaniGlobe, http://www.planiglobe.com, CC BY 2.0” in the image credit after the caption. (http://www.planiglobe.com/?lang=enl)

* Natural Earth - All maps are public domain. (http://www.naturalearthdata.com/about/terms-of-use/)

7. If the reviewer comments include a recommendation to cite specific previously published works, please review and evaluate these publications to determine whether they are relevant and should be cited. There is no requirement to cite these works unless the editor has indicated otherwise.

**Additional Editor Comments (if provided):** **Reviewers' Comments:**

**Comments to the Author**

1. Does this manuscript meet PLOS Digital Health’s publication criteria?

Reviewer #1: Yes

Reviewer #2: No

Reviewer #3: Yes

Reviewer #4: No

Reviewer #5: Yes

**********

2. Has the statistical analysis been performed appropriately and rigorously?

Reviewer #1: N/A

Reviewer #2: N/A

Reviewer #3: Yes

Reviewer #4: Yes

Reviewer #5: Yes

**********

3. Have the authors made all data underlying the findings in their manuscript fully available (please refer to the Data Availability Statement at the start of the manuscript PDF file)?

The PLOS Data policy

Reviewer #1: No

Reviewer #2: Yes

Reviewer #3: Yes

Reviewer #4: Yes

Reviewer #5: Yes

**********

4. Is the manuscript presented in an intelligible fashion and written in standard English?

Reviewer #1: Yes

Reviewer #2: Yes

Reviewer #3: Yes

Reviewer #4: Yes

Reviewer #5: Yes

**********

Reviewer #1: This manuscript presents a systematic review of AI applications in ESRD care and proposes a 3-stage framework. The work is useful from a digital health perspective. There are, however, several comments and/or suggestions that I would like to clarify with the authors.

1. The manuscript states that 124 studies were included after screening, yet some sections analyze larger number of studies (ie 1028 publications in keyword analysis). Can the authors comment and explicitly define/separate datasets used for a) systematic review synthesis vs 2) bibliometric analysis. If bibliometric analysis was conducted, this must be prespecified in the methods section.

2. Inclusion criteria require studies to involve ESRD patients receiving dialysis or transplantation (lines 160–163). However, several included studies appear to involve CKD screening, population-level risk detection, or non-ESRD cohorts, yet are classified under “Kidney Transplantation” or “ESRD care” in Extended Table 1. Can the authors explain this? Is this a misclassification?

3. While databases and general keyword domains are listed, full search strings are not provided for any database. Recommend adding full-database specific search strategy as a supplement.

4. In the 2-stage screening process, how many of the authors reviewed the study? Was this done independently? How were disagreements resolved? This needs to be explicitly stated.

5. Similarly, please provide inter-rate agreement.

6. The review repeatedly comments on lack of validation/interpretability, yet no structured risk-of-bias or quality appraisal tool is described. Recommend adding formal assessment tool.

7. What do the authors mean by "high quality" studies? Please define this terminology.

8. The results reported ~24% of studies performed external validation. Can the authors also define what qualifies as external (ie temporal split? public dataset)?

9. The three-stage evolutionary framework is central to the manuscript but lacks explicit criteria for assigning studies to stages. What was the rubric/criteria? Please justify.

10. Statements linking publication surges to COVID-19 or shifts in clinical priorities are speculative and not supported by formal analyses. Please temper and rephrase descriptive observations.

11. Terms such as “AI-ESRD,” “AI in ESRD,” and “renal AI systems” are used interchangeably without definition. Would suggest to standardize to lessen any confusion.

12. Authors reported "441 country affiliations" and top-10 country dominance. This is very difficult to interpret. Were affiliations counted per author? paper? or per institution?

13. The authors restricted to english peer-reviewed literature. What was the basis? Please provide justification.

14 The abstract highlights critical need to bridge gap between modelling and clinical utility. This is reasonable. But, elsewhere in the manuscript, it seems to imply an inevitable progression toward cognitive collaborators w/o even acknowledging that much of stage 3 remains unvalidated. Please tighten what your evidence supports.

Minor:

1. Consider adding raw counts alongside % throughout the results for clarity.

2. Figure legends should explicitly state which dataset is displayed. Figure titles should also be stand-alone descriptions for the data presented.

3. Always define acronyms at first mention.

4. Initial search is 5,000+ records not 1072. Please reconcile.

Reviewer #2: This systematic review intends to provide a comprehensive analysis of the historical evolution and current state of artificial intelligence (AI) applications in end-stage renal disease (ESRD) care and management, proposing a three-stage developmental framework to map AI’s progression from rule-based tools to cognitive agentes

In my perspective, the manuscript presents several methodological and structural issues that need to be addressed to ensure the rigor and transparency expected of a systematic review. The fundamental principle of a systematic review is reproducibility, and the methods described currently do not permit this. For instance, while keywords are provided, the complete search equations used across all databases are missing; these are crucial for reproducibility and should be included, preferably as supplementary material. Furthermore, the justification for including only English-language literature needs to be clearly explained, and the authors must explicitly state how many reviewers performed the screening and data extraction processes. Critically, there is no assessment of the risk of bias or quality appraisal of the included studies, which is essential for evaluating the reliability of the findings. Overall, the research questions appear to be more suited to a scoping review than a systematic review.

While the framework is innovative, its presentation could benefit from a more explicit definition of the boundaries and transitions between stages, particularly regarding the criteria for moving from one stage to the next.

The manuscript should expand on the limitations of current models in real-world settings, including issues related to overfitting, data drift, and the need for multicenter validation. It would be beneficial to elaborate on practical strategies for translating AI research into routine clinical practice, including usability, alert appropriateness, and clinician acceptance. Also, a more thorough discussion of regulatory frameworks, patient privacy, and equity in AI deployment would strengthen the manuscript.

Finally, the document is quite lengthy on some parts and would benefit significantly from being synthesized and streamlined to improve clarity, and the conclusion section assumes an unusual structure that should be revised.

Minor points:

British vs American English is mixed (e.g., modelling vs modeling)

Reviewer #3: Recommendations:

1. Validation Statistics Need Clarification

The abstract states 23.8% of Stage 2 models lack external validation, but Section 5.2.5 reports 78.8% employed formal validation with 23.8% having external validation. This appears contradictory and needs clarification. Is 23.8% the proportion with or without external validation? The clinical implications of this validation gap deserve more prominent discussion.

2. Stage 3 (LLM/Agent Systems) Analysis is Underdeveloped

Only 12 studies represent Stage 3, yet the framework positions this as a major evolutionary stage comparable to the others. Half of these studies lack formal validation, and the "hallucination" problem is mentioned but not quantified. Given the early stage of this technology, consider whether it should be presented as an emerging direction rather than an established stage.

3. Performance Metrics Reporting Inconsistencies

Multiple performance metrics are reported (AUC, accuracy, sensitivity, specificity, F1, C-index) without clear rationale for metric selection. Some studies report only accuracy while others report only AUC, making cross-comparison difficult. Create a standardized performance summary table organized by application domain and treatment modality.

4. External Validity and Clinical Translation Gap

The gap between model performance and clinical implementation is acknowledged but not deeply explored. What specific barriers prevent translation: regulatory, technical, or clinical acceptance issues? More discussion is needed on why high-performing models (AUC 0.80-0.90) aren't being adopted in clinical practice.

5. Methodological Issues - Search Strategy

The search strategy appears appropriate but there's no mention of searching clinical trial registries or grey literature. Were conference proceedings included? The exclusion of non-English studies may introduce language bias that should be acknowledged.

6. Methodological Issues - Data Extraction and Quality Assessment

The methods section does not specify whether data extractions were done in duplicate or how disagreements were resolved. Was study quality formally assessed using a validated tool? If quality assessment was not performed, this should be acknowledged as a limitation that may affect the strength of conclusions.

7. Results Section Organization

The results section is very long, and sections 3.1-3.7 (bibliometric analysis) feel disconnected from the core three-stage framework. Consider moving some bibliometric details to supplementary materials. The country/institutional analysis, while interesting, may not be essential to the main narrative.

8. Figure Quality and Clarity

Figure 9 is conceptually strong but visually dense, and Figure 12 tries to convey too much information simultaneously. Several figures have small text that may be difficult to read in print. Consider simplifying key figures, particularly Figures 12 and 14.

Reviewer #4: A novel systematic review of the Evolution of Artificial Intelligence in End-Stage Renal Disease Care and Management. This study shows methodological rigor but more clarity, modifications and possible retractions are needed in some sections of the manuscript with respect to some claims made.

Introduction:

- Readable but structurally repetitive with unnecessary prolongation of justifications across the paragraphs (ESRD complexity, AI advantages, gaps in prior review). Just to cite an example:

“traditional technical evaluation frameworks can no longer adequately reflect……next-generation AI systems” (Para 3)

“break through the traditional framework…… to examine evolution from rule-based to agent-based systems” (Para 5)

Methods:

Under search strategy:

“The search timeframe was set from January 2005 to June 2025, covering the critical period of rapid development of artificial intelligence technology in the healthcare domain, particularly the important phases of deep learning emergence and large language model proliferation”

The authors made an unverified claim here(may be historically inaccurate), as some records will suggest that deep learning in healthcare(with measurable impact)started with frameworks like  AlexNet, which had a major impact on the potential for medical imaging analysis.2012-2014 with LLM proliferation post 2022. The authors could have at least cited a reference to back up their claim which needs further clarity and provide the full historical AI timeline application in healthcare.

- The authors should provide more clarity on what qualifies as high quality studies as stated in the inclusion criteria

- The authors should also provide the criteria used to assign studies to the 3 stages mentioned in this study.

Results:

”Although growth rate has somewhat slowed, the output scale has reached new heights,”This is a claim the authors must either cite a reference to back or remove from the manuscript. The authors stated the 2022-2025 period experienced an exponential increase in AI studies exploring ESRD but how come the growth rate slowed down?

- Too much focus on institution and author analyses which is a distraction from measurable clinical impact.

- Reportage of metrics seem inconsistent across domains as seen in diagnostic support,monitoring and management, treatment modality. These metrics should align across board.

- The study will benefit from a table of the comparative analysis of the best performing models.

Discussion:

There is no dedicated discussion section

Limitations:

There is no dedicated limitation section

Overall: A novel study with impressive graphical illustrations and strong methodological input. The authors need to do a major revision to make this study more suitable and fit.

Reviewer #5: I did appreciate the review and the figure presentation. The methology is correct and is providing a way to understand the data at a glance. The reader can than look into the data in more details in the text. The figures illustrates AI dilemas and limitation. It is a great example and could be used for other disciplines such as radiology for example.

**********

what does this mean?). If published, this will include your full peer review and any attached files.

**Do you want your identity to be public for this peer review?**  If you choose “no”, your identity will remain anonymous but your review may still be made public.

For information about this choice, including consent withdrawal, please see our Privacy Policy

Reviewer #1: No

Reviewer #2: **Yes:**  Raquel Simões de Almeida

Reviewer #3: No

Reviewer #4: No

Reviewer #5: **Yes:**  Dr med. V. Grek

**********

**Figure resubmission:**

**Reproducibility:**

To enhance the reproducibility of your results, we recommend that authors of applicable studies deposit laboratory protocols in protocols.io, where a protocol can be assigned its own identifier (DOI) such that it can be cited independently in the future. Additionally, PLOS ONE offers an option to publish peer-reviewed clinical study protocols. Read more information on sharing protocols at https://plos.org/protocols?utm_medium=editorial-email&utm_source=authorletters&utm_campaign=protocols

# Author comment: Author response to Decision Letter 1

**DOI:** 10.1371/journal.pdig.0001635.r002

16 Apr 2026

Attachment Submitted filename: Response to Reviewers.docx

# Peer review: Decision Letter 1

**DOI:** 10.1371/journal.pdig.0001635.r003

**Authors:** Kezhi Li, Hongxing Luo

24 Jun 2026

Response to ReviewersRevised Manuscript with Track ChangesManuscript**Journal Requirements:** **Additional Editor Comments (if provided):** **Reviewers' Comments:**

**Comments to the Author**

Reviewer #1: All comments have been addressed

Reviewer #4: All comments have been addressed

**********

publication criteria?

Reviewer #1: Partly

Reviewer #4: Yes

**********

3. Has the statistical analysis been performed appropriately and rigorously?

Reviewer #1: Yes

Reviewer #4: Yes

**********

4. Have the authors made all data underlying the findings in their manuscript fully available (please refer to the Data Availability Statement at the start of the manuscript PDF file)?

The PLOS Data policy

Reviewer #1: Yes

Reviewer #4: Yes

**********

5. Is the manuscript presented in an intelligible fashion and written in standard English?

Reviewer #1: Yes

Reviewer #4: Yes

**********

Reviewer #1: I commend the authors for taking the time to revise their work, and providing a detailed point-by-point response. The review is now much clearer and more methodologically transparent. That said, I do have some questions//concerns upon reading the revised manuscript.

MAJOR:

1. The authors state that all 100 included studies directly involve ESRD patients undergoing HD, or PD, or kidney transplantation. However, some Stage 3 examples cited appear to be general medical AI systems rather than ESRD-specific studies. Can the authors reconcile this?

2. DECIDE-AI is primarily a reporting guideline/framework for early-stage AI decision-support evaluation rather than a conventional risk-of-bias tool. Avoid describing this as ROB.

3. Thank you for providing the inter-rater agreement. The agreement on tool selection was K=0.87 and the authors later states that stage assignment agreement was quantified as part of the tool-selection kappa. As far as I am concerned, tool selection and stage assignment are not the same.

4. The manuscript reports QUADAS-2 observed agreement ranging from 28%-67%. I suppose there were some disagreements in some domains. Can the authors discuss how this affected confidence in their assessment? How was final consensus achieved?

5. Please remember to temper language used in the manuscript. Avoid words like "breakthrough", "excellent"....which recur quite frequently. Use neutral language.

6. The authors now state that PRISMA 2020 was followed. My question is was a review protocol registered?

7. Similarly, please avoid “AI can significantly improve medical efficiency” unless the statement is specifically supported by included studies.

MINOR:

1. Please standardize terminology: “AI in ESRD,” “ESRD care,” and “ESRD management” should not be used interchangeably...unless intended.

2. AUC-ROC and AUROC should be defined consistently. Also avoid switching between the 2 terms.

3. Please define all abbreviations in the footnote for the tables provided.

4. What does N/A mean in table 3? Not applicable? Not assessed? Not reported?

Reviewer #4: I will suggest the authors provide more clarity on the inclusion criteria as not all dialysis patients have ESRD. Dialysis may be needed for Acute kidney injury and other cases....Please clarify if the eligible studies included ONLY patients with ESRD receiving dialysis treatment and also explain how non ESRD dialysis populations were excluded.

**********

what does this mean?). If published, this will include your full peer review and any attached files.

**Do you want your identity to be public for this peer review?**  If you choose “no”, your identity will remain anonymous but your review may still be made public.

For information about this choice, including consent withdrawal, please see our Privacy Policy

Reviewer #1: No

Reviewer #4: No

**********

**Figure resubmission:**

**Reproducibility:**

To enhance the reproducibility of your results, we recommend that authors of applicable studies deposit laboratory protocols in protocols.io, where a protocol can be assigned its own identifier (DOI) such that it can be cited independently in the future. Additionally, PLOS ONE offers an option to publish peer-reviewed clinical study protocols. Read more information on sharing protocols at https://plos.org/protocols?utm_medium=editorial-email&utm_source=authorletters&utm_campaign=protocols

# Author comment: Author response to Decision Letter 2

**DOI:** 10.1371/journal.pdig.0001635.r004

28 Jun 2026

Attachment Submitted filename: Response_to_Reviewers_auresp_2.docx

# Editor report: Decision Letter 2

**DOI:** 10.1371/journal.pdig.0001635.r005

**Authors:** Hongxing Luo, Xiangbin Meng

20 Jul 2026

From Assistant to Collaborator: A Systematic Review of the Evolution of Artificial Intelligence in End-Stage Renal Disease Care and Management

PDIG-D-25-01132R2

Dear Huang and co-authors,,

We are pleased to inform you that your manuscript 'From Assistant to Collaborator: A Systematic Review of the Evolution of Artificial Intelligence in End-Stage Renal Disease Care and Management' has been provisionally accepted for publication in PLOS Digital Health.

Before your manuscript can be formally accepted you will need to complete some formatting changes, which you will receive in a follow-up email from a member of our team.

Please note that your manuscript will not be scheduled for publication until you have made the required changes, so a swift response is appreciated.

IMPORTANT: The editorial review process is now complete. PLOS will only permit corrections to spelling, formatting or significant scientific errors from this point onwards. Requests for major changes, or any which affect the scientific understanding of your work, will cause delays to the publication date of your manuscript.

If your institution or institutions have a press office, please notify them about your upcoming paper to help maximize its impact. If they'll be preparing press materials, please inform our press team as soon as possible -- no later than 48 hours after receiving the formal acceptance. Your manuscript will remain under strict press embargo until 2 pm Eastern Time on the date of publication. For more information, please contact digitalhealth@plos.org.

Thank you again for supporting Open Access publishing; we are looking forward to publishing your work in PLOS Digital Health.

Best regards,

Xiangbin Meng, M.D., Ph.D.

Academic Editor

Hongxing Luo, M.D., Ph.D.

Section Editor

PLOS Digital Health

***********************************************************

**Additional Editor Comments (if provided):**

Dear Dr. Huang and co-authors,

Thank you for the careful and comprehensive revision of your manuscript “From Assistant to Collaborator: A Systematic Review of the Evolution of Artificial Intelligence in End-Stage Renal Disease Care and Management” (PDIG-D-25-01132R2).

We are pleased to inform you that the revised version has satisfactorily addressed all previous comments. In particular, the clarification of the ESRD-specific corpus versus broader contextual citations, the correct characterization of DECIDE-AI, the independent reporting of stage-assignment inter-rater agreement, the discussion of QUADAS-2 concordance, the neutralization of language, the transparent statement regarding the absence of prospective registration, and the tightened inclusion/exclusion criteria for ESRD versus AKI populations have substantially improved the accuracy, transparency, and balance of the paper.

The three-stage developmental framework is now cleanly supported by the evidence base and constitutes a useful contribution to the literature.

We are therefore delighted to accept the manuscript for publication in PLOS Digital Health, pending final production checks (figure formatting via the PLOS NAAS tool, ORCID verification, and any minor copy-editing).

Congratulations on the work. We look forward to seeing the final published article.

Sincerely,

Xiangbin Meng, M.D., Ph.D.

Academic Editor

PLOS Digital Health

**Reviewer Comments (if any, and for reference):**
